# Characterizing Visual Neurosurgical Expertise in Brain MRI Visualization Using Eye-Tracking and 3D Fractal Dimension Analysis

**DOI:** 10.3390/jemr19030062

**Published:** 2026-06-02

**Authors:** Poonam Kumari, Ghasem Azemi, Carlo Russo, Antonio Di Ieva

**Affiliations:** Computational NeuroSurgery (CNS) Lab, Macquarie Medical School, Faculty of Medicine, Health and Human Sciences, Macquarie University, 75 Talavera Road, Sydney, NSW 2109, Australia; ghasem.azemi@mq.edu.au (G.A.); carlo.russo@mq.edu.au (C.R.)

**Keywords:** scanpath, eye gaze, MRI, neurosurgery, visual stimuli, eye-tracking, fixation duration, visual attention, visual expertise, fractal dimension

## Abstract

Eye-tracking has been utilized to characterize visual behavior in medical image visualization and interpretation, yet neurosurgeons remain underrepresented. Characterizing neurosurgery-specific visual expertise is important for understanding expert search strategies, informing training, and developing computational models. This study examined gaze behavior in naïve observers (*N_p_* = 29), neurosurgery registrars (*N_p_* = 16), and consultant neurosurgeons (*N_p_* = 24), viewing normal (*N_p_* = 20) and pathological (*N_p_* = 19) brain MR images under a free-viewing paradigm. To capture expertise-related characteristics, we analyzed two features at each fixation location: (i) fixation duration, reflecting temporal allocation of visual attention, and (ii) three-dimensional fractal dimension (3DFD) around each fixation location, quantifying local structural complexity. To assess pathological-type effects, we grouped similar pathologies into five stimulus groups. Linear mixed-effects modelling revealed systematic expertise-related differences, with experts exhibiting longer fixation durations in pathological stimulus groups and pathology-type-dependent complexity sampling. Combined fixation duration and 3DFD features captured complementary aspects of visual expertise, improving Random Forest classifier’s accuracy (>93%) compared to individual features, for all five stimulus groups. These findings highlight neurosurgery-specific markers of visual expertise and demonstrate that combining behavioral and image-derived features could underpin computational models and training tools that emulate expert-level strategies in neurosurgical image interpretation. Future work should evaluate its applicability to other medical domains.

## 1. Introduction

Understanding how humans develop and apply visual expertise is a fundamental research question in cognitive neuroscience, psychology, and applied fields such as education, human–computer interaction, and medical training. Visual expertise governs how individuals process complex visual environments, make decisions, and respond to stimuli. Understanding the mechanisms underlying visual expertise is, therefore, critical for advancing theories of perception and designing effective training programs in some emerging applications, such as medical image visualization and interpretation.

To investigate the mechanisms underlying visual expertise, researchers have employed a range of methodologies, including functional magnetic resonance imaging (fMRI) [1], electroencephalography (EEG) [2], magnetoencephalography (MEG) [1], and eye-tracking [3]. Among these methodologies, eye-tracking offers a low-cost, portable and computationally simpler tool to study the visual behavior underlying expertise. In a typical eye-tracking study, participants are presented with stimuli, and their eye gaze patterns are recorded using an eye-tracking device. Here, eye tracking is employed as a sensing modality in which gaze signals are acquired at high temporal resolution and subsequently analyzed to characterize visual behavior during image viewing.

A recent focus has emerged in eye-tracking research where the goal is to objectively characterize physicians’ visual expertise. Prior work demonstrates that medical experts possess distinctive perceptual and cognitive strategies for distinguishing normal from pathological images [4,5,6,7,8,9,10]. Translating this domain-specific expertise into computational models could enable the development of “expert-like” computer systems for multiple applications [11,12]. It could enable an automated identification of abnormal medical images [13,14,15], real-time feedback for trainees during image interpretation, and objective evaluation of training progress in medical education.

Importantly, the “expert-like” computer systems must be tailored to the specific domain of expertise. For example, radiologists develop visual strategies through extensive experience interpreting diverse imaging modalities, whereas neurosurgeons acquire expertise shaped by their neurosurgical training, neuroanatomical familiarity, and case-specific decision-making demands. Because these cognitive and perceptual processes differ across medical specialties, an “expert-like” computer system must be informed by features that accurately capture the visual behavior unique to the target expert group. Consequently, identifying neurosurgery-specific markers of visual expertise requires dedicated data collection from neurosurgeons themselves, along with analytical methods capable of converting their distinct gaze behaviors into reliable, computationally interpretable features.

Previous research has examined eye-tracking data from medical experts from different domains. Many of the medical experts were radiologists [4,5,6,7,8,9,16] presented with stimuli such as chest radiographs [6,7,17], breast mammographs [4,5,8,9], or brain magnetic resonance images (MRIs) [16,18]. Few studies have reported eye-tracking data collected from surgeons. For example, Li et al. [19] compared gaze behaviors of two cardiac surgeons, the medical experts, and 12 novices who were medical residents and pre-residency medical students. The eye-tracking data were collected by performing a simulated surgical procedure. Khan et al. [20] analyzed eye-tracking data from 16 expert surgeons and novices during laparoscopic cholecystectomy operations using both live and video stimuli. Other similar studies include 14 laparoscopic surgeons wearing eye trackers while using a surgical simulator [21]. These studies consistently report significant differences in visual behavior between experts and novices, highlighting the role of visual expertise in guiding search strategies.

Despite growing interest in eye-tracking research, in our review of prior eye-tracking research, we found that there have been very few attempts to characterize visual expertise in neurosurgeons. Dalveren et al. [22] noted that this gap could be due to the difficulty in recruiting neurosurgeons for experimental studies. Consequently, prior work has been limited to small samples, such as 13 neurosurgeons performing computer-based simulated surgical tasks [22] and 9 neurosurgeons performing cutting and suturing tasks under a surgical microscope [23]. Notably, none of these studies have employed free-viewing tasks as stimuli. Our earlier work by Suman et al. in 2021 [10] used free-view tasks as stimuli but included only 4 neurosurgeons among 31 experts. Therefore, the conclusions in the Suman et al. 2021 [10] study were biased towards the visual strategies of radiologists. Since developing a bias-free domain-specific “expert-like” computer system of neurosurgeons would require data collected explicitly from this group, a focused study to identify the features characterizing their visual attention is necessary.

Prior studies have investigated a wide range of eye-tracking features to distinguish naïve observers from medical experts, aiming to capture markers of visual expertise. These features fall broadly into three methodological categories. The first category is the spatial event-level features, such as fixation duration [4,9,18,19,23], dwell time [5,9,18,24], number of returns to a region [5,9,18], and the spatial distribution of gaze [19], which collectively reflect how observers allocate visual attention across an image. The second category comprises time-series features, including saccade duration [9,18,19,23], blink duration [19], shifts of visual focus between experts and novices [19], mean latency and mean peak velocity of saccades [24], the Hurst exponent derived from R/S and DFA analyses [10], and two-dimensional fractal dimension (FD) calculated from the eye-tracking time-series data [10]. All these features characterize the temporal structure and long-range dependencies in gaze behavior. The third category is the temporal sequence-based scanpath features, such as ScanMatch [3,16], MultiMatch [25] and SoftMatch [26], methods that quantify the similarity of fixation sequences. Together, these approaches provide complementary insights into the spatial, temporal, and sequential dynamics that underpin expert visual search strategies.

Numerous studies have established that gaze behaviors reflect underlying cognitive processes such as attention allocation, perceptual load, and information processing. Longer fixation durations typically signal deeper attentional engagement with relevant visual information, whereas higher saccade rates indicate more exploratory search behavior [27]. Reduced blink rate and shorter saccade amplitudes similarly correspond to increased attentional demand. These cognitive interpretations provide important context for examining expertise-related differences in visual behavior, as they clarify how fixation- and saccade-based metrics serve as proxies for the strategies experts use when interpreting complex medical images [28].

Despite recent advances, eye-tracking studies remain comparatively limited in scope and methodological diversity, and important aspects of the visual information guiding gaze behavior are poorly understood. A notable limitation of existing work is that image content is typically treated as an implicit background factor rather than a quantifiable variable. As a result, most studies focus on behavioral metrics such as fixation duration, fixation count, or scanpath patterns, without explicitly characterizing the structural properties of the image regions being fixated. Consequently, it remains unclear how expertise-related gaze differences reflect systematic interactions between visual attention and underlying image complexity.

Fractal dimension (FD) has been used in eye-tracking research as a quantitative descriptor of scanpath complexity, with two-dimensional FD capturing irregularity and self-similarity in gaze behavior during medical image interpretation [10]. However, these studies have focused on the trajectory of visual search rather than the structural geometric complexity of the image itself. Therefore, the research question of how brain MRI spatial complexity interacts with where observers look remains unexplored. Spatial complexity is critical in neurosurgical image visualization and interpretation because pathological entities such as tumors, cerebrovascular malformations, and inflammatory changes often exhibit heterogeneous, irregular structures that challenge perceptual tasks and diagnostic reasoning [29,30,31].

Despite growing interest in expert–novice differences, no previous work has jointly modelled fixation behavior and local image complexity of the underlying image regions, a gap that remains largely unexplored in visual expertise characterization. Addressing this gap could reveal whether experts allocate gaze to diagnostically informative regions rather than being driven by surface-level complexity, offering insights into mechanisms of visual expertise. Such insights could, in turn, support developing computational models for training and decision support in many applications, including neurosurgery-specific models.

The main goal of this work is to design a focused study to characterize the visual expertise of neurosurgeons without any bias from the data of other medical experts. Participants from three groups of naïve observers, neurosurgery registrars, and consultant neurosurgeons viewed normal and pathological brain MR images under a free-viewing paradigm. To capture expertise-driven gaze behavior, we jointly modelled two features at each fixation location: (i) fixation duration, which reflects temporal allocation of visual attention, and (ii) three-dimensional fractal dimension (3DFD) of the underlying image region, which quantifies local structural complexity. Fixation duration reflects extended, task-relevant attentional engagement, while 3DFD at fixation locations captures the local structural heterogeneity of sampled regions. Together, these features denote complementary indicators of the mechanisms hypothesized to underline expert viewing in neurosurgical MRI. This integrated approach enables us to examine how experts distribute attention relative to image complexity, providing neurosurgery-specific markers of visual expertise that can inform future “expert-like” computational models. Note that our selected features fall within the first eye-tracking features category, i.e., the spatial event-level features category.

To explicitly test how fixation behavior and local image structural complexity contribute to neurosurgery-specific visual expertise, we adopted an integrated analytical framework combining inferential and predictive modelling. Using linear mixed-effects modelling, group-level expertise effects were assessed while accounting for repeated observations within participants and stimuli, thereby enabling inference about systematic expertise-related differences. In contrast, machine-learning classifiers provide a means of evaluating the discriminative value of fixation duration and 3DFD features by learning non-linear decision boundaries in multivariate feature spaces. Combining these approaches allows both inferential (linear mixed-effects) and predictive (machine-learning) aspects of neurosurgical visual expertise to be examined within an integrated analytical framework.

Three hypotheses were tested in this study using an integrated analytical framework.

- **H1**: Fixation duration will be longer for neurosurgery experts than for naïve observers when viewing pathological brain MRIs, indicating selective, task-relevant temporal allocation of visual attention.
- **H2**: The structural complexity of image regions fixated by observers, quantified using 3DFD, will be lower for neurosurgery experts than for naïve observers when viewing pathological brain MRIs, but in a pathology-dependent manner, reflecting prioritization of diagnostically informative structure over surface-level complexity.
- **H3**: Joint modelling of fixation duration and 3DFD, reflecting complementary local temporal and structural sampling, will improve discrimination between naïve observers and neurosurgery experts compared with either feature alone, when evaluated using non-linear machine-learning classifiers.

By testing these hypotheses across multiple brain MRI stimulus groups, this study examines how temporal fixation behavior and spatial image complexity jointly encode neurosurgery-specific visual expertise. To our knowledge, no prior study has jointly modelled fixation behavior and local image-derived structural complexity at fixation locations in neurosurgeons. This is the first systematic study to apply 3DFD to neurosurgical eye-tracking data and to integrate 3DFD with fixation-duration metrics for characterizing visual expertise in neurosurgical image visualization. By explicitly contrasting linear statistical inference with non-linear classification performance, this work establishes a computational foundation for future neurosurgery-specific expert-like systems.

## 2. Materials and Methods

### 2.1. Participants

The participants for this study were recruited via email announcement and word-of-mouth. Each participant was contacted individually to explain the study, experiment setup, and inclusion/exclusion criteria.

**Expert participants**. The inclusion criteria for neurosurgery expert participants in our study were based on whether the participant held a board certification or other post-graduate neurosurgery qualification. Accordingly, the expert participants in our study were a combination of consultant neurosurgeons (*n* = 24) and trainee neurosurgery registrars (*n* = 16).

The expert participants in our study were recruited in two sets. The first set of participants (*N_p_* = 27) was recruited from the 77th Annual Scientific Meeting of the Neurosurgical Society of Australasia, which took place in Sydney (Australia) on 14–16 September 2022. The second set of participants (*N_p_* = 13) was recruited from Macquarie University, Sydney, Australia. Overall, there were 11 females, 28 males, and 1 preferred not to say. The expert participants were aged from 26 to 61 years. All participants reported normal or corrected-to-normal vision.

**Naïve participants**. The inclusion criteria for naïve participants in our study were a lack of any prior experience reading medical images. Accordingly, the naïve participants (*N_p_* = 29) in our study were recruited from a population of Macquarie University students and staff. There were 16 female and 13 male participants, aged from 23 to 52 years. All participants reported normal or corrected-to-normal vision.

#### Participant Grouping for Visual Expertise Analysis

**Groups**: The expert group in our study was split into two groups. The neurosurgery consultants (*N_p_* = 24) were grouped into the expert consultant group (EC), and the neurosurgery registrars (*N_p_* = 16) into the expert registrar group (ER). A naïve group (N) of participants (*N_p_* = 29) was formed as the third group in our study. The resulting participant groups and demographic characteristics are presented in Table 1.

### 2.2. Stimuli

Each participant in this study was presented with a set of 39 de-identified T1-weighted brain MRIs as stimuli. The stimuli were two-dimensional MRI images stored as PNG files. The stimulus images were standardized to a size of 1920 × 1080. The MRI stimuli set presented to participants was a mixture of images taken in different views (axial, sagittal, and coronal). Twenty brain MRIs did not contain any pathology, and the other 19 included some pathologies (e.g., brain or skull base tumors and aneurysms). The normal and pathological images were intermixed, and a fixed sequence of the intermixed images was created. This sequence of images was then presented to each participant during their eye-tracking data collection session.

#### Stimulus Grouping for Visual Expertise Analysis

**Figure A1:**
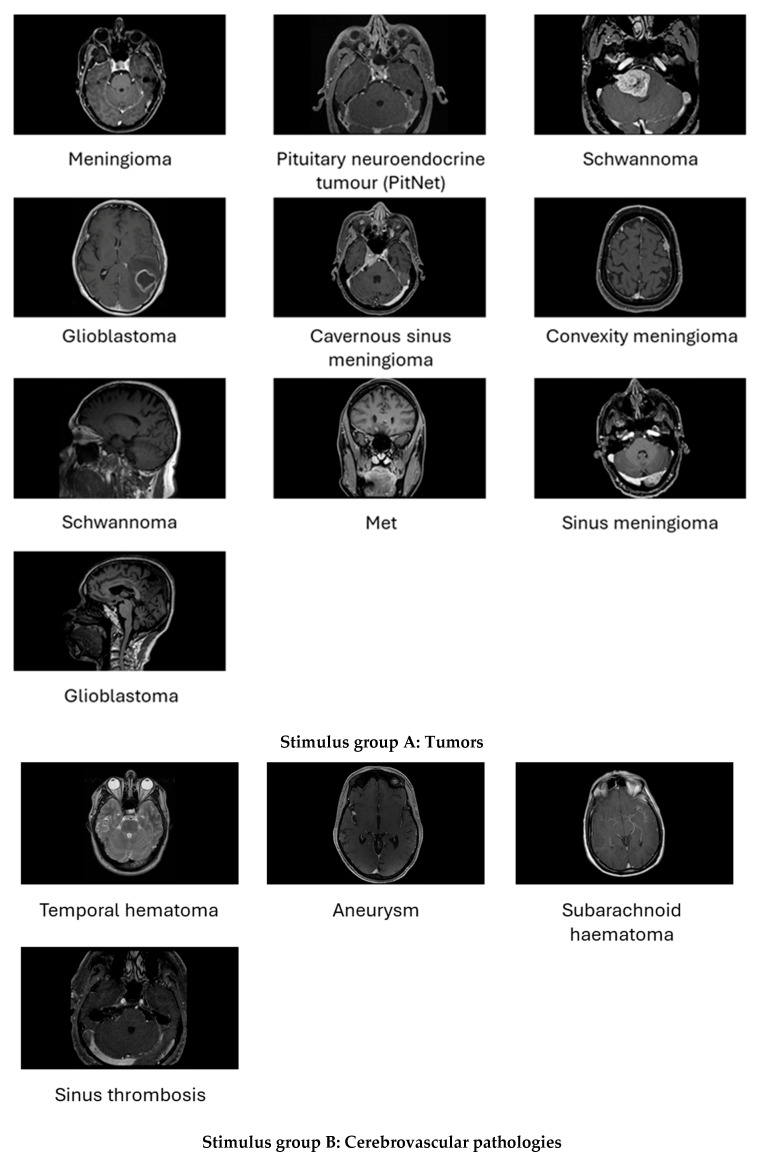

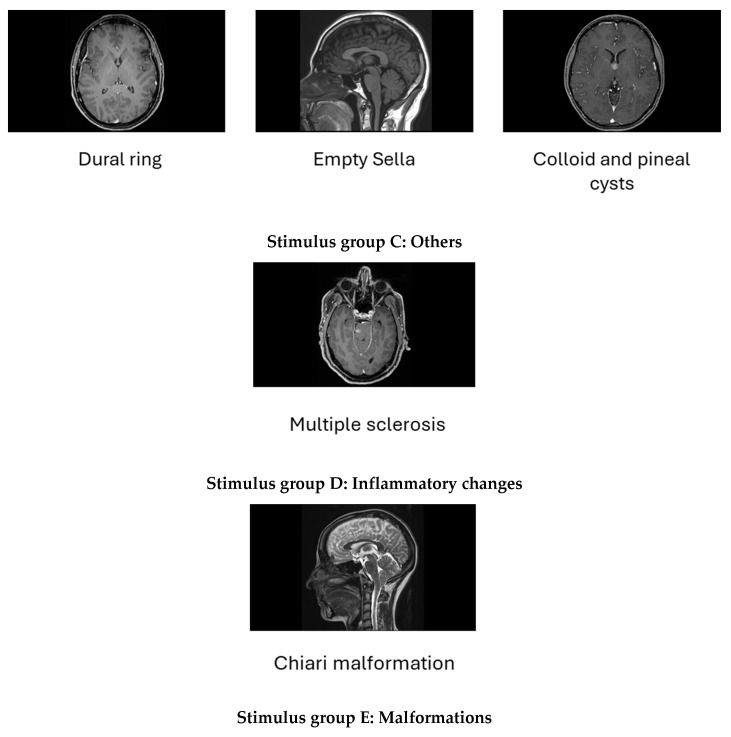
MR images used as pathological stimuli in this study. Stimulus groups A–E correspond to the stimulus groups with pathology types of tumors, cerebrovascular pathologies, others, inflammatory changes, and malformations. The name of the pathology is shown for each of the stimulus images.

Seven stimulus groups (A–E, StimP, StimN) were constructed from the 39 stimulus images for visual expertise analysis. Six groups (A–E, StimP) contained a unique collection of brain pathological MR images with various types of pathology. These groups represented a diverse set of MR images commonly seen by neurosurgeons in their neurosurgery practice. One group (StimN) contained normal brain MR images. The full taxonomy of stimulus groups, including image type, pathological status, and image counts, is provided in Table 2. The pathological images used in our study are provided in Appendix A Figure A1.

### 2.3. Apparatus

**Figure 1:**
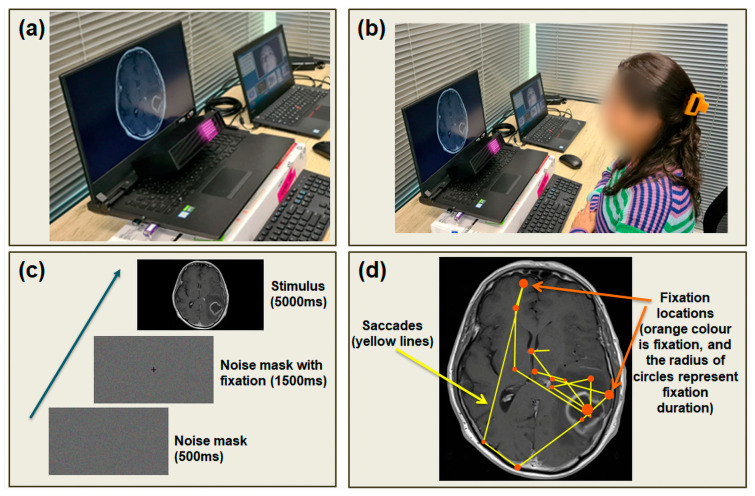
Eye-tracking experiment. (**a**) Eye tracker; (**b**) data collection setup showing participant’s position with respect to Eye Tracker; (**c**) stimulus presentation sequence; (**d**) eye-tracking data (scan path) overlayed on a stimulus.

A portable eye tracker (EyeLink Portable Duo 1000) from SR Research Ltd. (Ottawa, ON, Canada) was used in this study. Stimuli were presented on a 15-inch laptop screen with a 1920 × 1080 display resolution operating at a 60 Hz refresh rate. The eye-tracking system operates based on infrared video-oculography, using high-speed optical sensors to continuously capture pupil and corneal reflection signals. Ocular movements were measured using the eye-tracker at a sampling rate of 1000 Hz. Figure 1 shows an illustration of our experimental setup with this eye-tracker.

### 2.4. Ethics Approval and Consent

All procedures were approved by the Macquarie University Medicine and Health Sciences Subcommittee (Ref: 52021919128177; approved on 18 May 2021). As per ethics approval, this study meets the requirements set out in the National Statement on Ethical Conduct in Human Research 2007 (updated July 2018), which embodies the ethical principles of the Declaration of Helsinki 1964. As per the ethics approval, this study falls within the category of “Clinical research that is not a clinical trial—observational study”.

Written consent was obtained from all participants prior to data collection.

### 2.5. Free-View Task Study Design

A free-view task study was designed where participants were only required to view the stimulus images and did not engage in any additional tasks. Since no specific diagnostic task was imposed, the fixation strategies were expected to reflect spontaneous expertise-driven search patterns rather than task-directed instructions.

### 2.6. Experimental Procedure

#### 2.6.1. Instructions to Participants Before Data Collection Session

Each participant was asked to complete a consent form and demographic questionnaire. This questionnaire included their age, neurosurgery expertise in reading brain MRIs (if any), and the average number of radiological cases reviewed per week. Following the completion of forms, the stimulus presentation sequence was explained to the participant with the aid of an illustration printed on paper.

A stimulus presentation sequence is demonstrated in Figure 1c. Participants were informed that they can view anywhere on the *noise mask* but must fixate centrally at the cross on the *noise mask with fixation*. Participants were also informed that following the two noise mask images, they would view a series of stimulus images on the laptop screen but were free to view anywhere on those stimulus images.

#### 2.6.2. Eye-Tracker Setup and Calibration Before Data Collection Session

The operational mode of the eye-tracker was chosen as *head free-to-move Remote mode* to allow data collection without any head support. Participants were asked to sit on a chair as illustrated in Figure 1b. A target sticker was placed on their forehead to aid the eye-tracker’s software in finding the target-to-camera distance between 480 and 520 mm. The eye-tracker was then calibrated using a 13-point calibration procedure. During calibration, participants were asked to keep their heads still. The laptop display was subtended to a maximum visual angle of 32° horizontally and 25° vertically. It was ensured that the calibration for both eyes was good in the eye-tracking software, with a calibration error of <1°.

#### 2.6.3. Data Collection Session

Short rest breaks were provided between blocks for the participants experiencing fatigue during their recording sessions. Throughout the data collection session, participants were asked to keep their heads still. The sequence of images (trial) consisted of a noise mask presented for 500 ms, a noise mask with cross fixation presented for 1500 ms, and then a stimulus image presented for 5000 ms. Each trial started with a 1-point calibration to ensure that participants were properly calibrated throughout the experiment. The scanpath (eye gaze) data were collected for each of the 39 trials at a sampling rate of 1 KHz. Therefore, there were 5000 samples in the scanpath data per trial.

### 2.7. Eye-Tracking Raw Data Processing and Report Generation

The raw data acquired from the eye-tracker comes in a form called EyeLink Data Files (EDF). The EDF files contain time-stamped gaze signals captured by the optical sensor in the eye-tracker, including eye position samples recorded at the device sampling rate. During this preprocessing stage, the raw gaze signals were parsed into discrete fixation and saccade events using the eye-tracker’s “EyeLink host online parser” algorithm, which is a velocity- and acceleration-threshold-based algorithm executed during data recording. In our experiments, one EDF file was generated for each participant. These EDF files were subsequently processed offline using EyeLink Data Viewer software (version 4.2.1) to generate reports for each participant [32]. Only the right eye’s data were utilized in this study for analysis.

### 2.8. Feature Extraction

All features analyzed in this study were derived from fixation events detected from the eye-tracking sensor’s gaze signals, linking the sensor-level measurements to quantitative descriptors of visual behavior.

#### 2.8.1. Fixation Duration

For each participant, a report was generated using EyeLink Data Viewer software (version 4.2.1) containing the coordinates of the fixation locations for each stimulus viewed and the duration of the fixation at that location. A template for this report is provided in Appendix A Table A1. Let n represent the index of the n-th fixation location, and $x\left(n\right)$ and $y\left(n\right)$ represent the horizontal and vertical coordinates of the fixation location at n, respectively. Then, the eye’s fixation position at n can be represented with a point in 2D space as:

(1) $$Pos\left(n\right)=\left(x\left(n\right),y\left(n\right)\right)$$

Let the eye’s fixation duration at $Pos\left(n\right)$ be represented as $FixDur\left(n\right)$. Let us denote the participant by P and the stimulus by s. From each participant’s report, we extracted all fixation durations for each stimulus s and arranged them in an array as:

(2) $${FixDur}_{P}^{s}=\left\{{FixDur}_{P}^{s}\left(1\right),{FixDur}_{P}^{s}\left(2\right),\cdots ,{FixDur}_{P}^{s}\left(N_{P}^{s}\right)\right\}$$

Here, $N_{P}^{s}$ denotes the number of fixations for stimulus *s* and participant P. We then sorted the fixation durations for the stimulus s in descending order as:

(3) $${FixDur_{decend}}_{P}^{s}=decending\left\{{FixDur}_{P}^{s}\left(1\right),{FixDur}_{P}^{s}\left(2\right),\cdots ,{FixDur}_{P}^{s}\left(N_{P}^{s}\right)\right\}$$

Let the first $M_{P}^{s}$ maximum fixation durations of this sorted fixation duration array be represented as:

(4) $${FixDur_{decend_{firstMmax}}}_{P}^{s}=\left\{{FixDur_{decend}}_{P}^{s}\left(1\right),{FixDur_{decend}}_{P}^{s}\left(2\right),\cdots ,{FixDur_{decend}}_{P}^{s}\left(M_{P}^{s}\right)\right\}$$

In this study, we empirically choose the $M_{P}^{s}$ = 10, which corresponds to the first 10 maximum fixations durations for each stimulus for each participant.

1. Heat map of fixation duration for visualization of fixations

The heat map of fixation durations was calculated for each stimulus and for each participant using the EyeLink Data Viewer software (version 4.2.1) provided by EyeLink [32]. In this software, the heat map is generated by applying a 2D Gaussian filter to each of the fixations. The center of the Gaussian function is chosen as the location of fixation. The width of the Gaussian function is determined by an adjustable standard deviation value, i.e., sigma, which represents the visual angle in degrees. A larger sigma value results in a greater area being affected by the fixation. We set sigma as 1.0 degrees, which is the default setting in the EyeLink Data Viewer software (version 4.2.1). In the software, the height of the Gaussian function is weighted by the duration of the fixation.

#### 2.8.2. Three-Dimensional Fractal Dimension (3DFD)

Fractal dimension (FD) is a metric that quantifies local geometric complexity, which may arise from spatial irregularity or heterogeneity within an image. In this study, the computation of FD for each pixel of the stimulus image is referred to as the 3DFD map. The 3DFD map represents the complexity of the structures present in the stimulus image. For each fixation point $Pos\left(n\right)$, the corresponding fractal dimension value was extracted from the 3DFD map. Let the 3DFD value at $Pos\left(n\right)$ be represented as $FD3D\left(n\right)$. Thus, for every fixation location, $FD3D\left(n\right)$ denotes the local structural complexity of the image region that attracted the observer’s gaze. We collected the 3DFD values for each participant P and stimulus s as:

(5) $${FD3D}_{P}^{s}=\left\{{FD3D}_{P}^{s}\left(1\right),{FD3D}_{P}^{s}\left(2\right),\cdots ,{FD3D}_{P}^{s}\left(N_{P}^{s}\right)\right\}$$

Let ${FD3D_{decend}}_{P}^{s}$ represent the sorted values of ${FD3D}_{P}^{s}$ arranged in descending order of fixation durations. The ${FD3D_{decend}}_{P}^{s}$ values corresponding to the first $M_{P}^{s}$ maximum fixation durations will then be represented as:

(6) $${FD3D_{decend_{firstMmax}}}_{P}^{s}=\left\{{FD3D_{decend}}_{P}^{s}\left(1\right),{FD3D_{decend}}_{P}^{s}\left(2\right),\cdots ,{FD3D_{decend}}_{P}^{s}\left(M_{P}^{s}\right)\right\}$$

1. Generation of 3DFD map from stimulus image

The 3DFD maps were calculated for each of the 39 stimulus images by implementing the box counting method [33]. The MRI stimuli were analyzed as two-dimensional grayscale images rather than as reconstructed volumetric MRI datasets. For 3DFD computation, each grayscale MRI image was represented as a three-dimensional intensity surface, where the x- and y-axes corresponded to image coordinates, and the *z*-axis corresponded to grayscale intensity. Therefore, the term 3DFD refers to the fractal dimension of the grayscale intensity surface derived from each 2D MRI image, rather than to the fractal dimension of a volumetric brain reconstruction from multiple 2D MRI slices. 

There were two settings in our implementation of the box counting method: *shift* and *patchsize*. For each point within the stimulus image, a moving window of *patchsize* × *patchsize* = 100 × 100 was created around the point, and the grayscale pixel values included in this patch were used to calculate the 3DFD value at that point. A *shift* × *shift* = 10 × 10 was used to move the patch through the image from left-to-right and from top-to-bottom. We implemented the box counting method for the calculation of 3DFD maps in the Python 3.10 programming language. Our implementation generated the 3DFD values of each stimulus in a 3-column csv format. A MATLAB code was written to convert the 3-column CSV file into an image of the same size as the stimuli image.

2. Foveal vision-driven processing of 3DFD maps to calculate $FD3D\left(n\right)$

For each stimulus *s*, all the fixation points $\left\{F\left(n\right)\;|\;n\in \left[1,N_{P}^{s}\right]\right\}$ were read from their eye-tracking data, and circular regions of interest (ROIs) were created by taking these points as centres. Foveal vision is defined as the central 1.5–2 degrees of the visual field. Vision within the fovea is generally called central vision. Considering the foveal vision, the radius of the ROI was taken as 1.5 degrees because this approximates the central foveal field used for detailed visual inspection at fixation. This corresponded to a radius of 30 pixels in our stimulus image. Mean values of the pixels within the ROIs were taken from the 3DFD maps and used to calculate $FD3D\left(n\right)$.

After calculating $FD3D\left(n\right)$ for all fixation locations, the values corresponding to the $M_{P}^{s}=10$ longest fixation-duration locations were selected for the main analysis. Thus, the $M_{P}^{s}=10$ points used in the analysis refer to the ten longest fixation-duration locations for each participant–stimulus pair, not to MRI slices or anatomical sections. At each of these fixation locations, the local 3DFD value was extracted from the precomputed 3DFD map described in item 1 of Section 2.8.2.

### 2.9. Linear Mixed-Effects Modelling and Statistical Analysis

#### 2.9.1. Model Equations

Fixation duration model:

(7) $$FixationDuration\sim 1+Group\times ImageType+\left(1|Participant\right)+\left(1|Stimuli\right)$$

3DFD model:

(8) $$FD3D\;\sim \;1+Group\times ImageType\;+\left(1\;|\;Participant\right)+\;\left(1\;|\;Stimuli\right)\;$$

These base models were fitted separately for each of the stimulus groups (A–E, StimP) by combining the data from these pathology stimulus groups with the normal stimuli group (StimN).

In both models, $Group=\left\{N,EC,ER\right\}$ and $ImageType=\left\{Normal,Pathology\right\}$ were treated as fixed effects using naïve observers (*N*) and Normal image-type as the reference categories, respectively. An interaction term Group×ImageType was included in the model to test if the type of pathology (tumor, cerebrovascular pathologies etc.) affects the differences across $\left\{N,EC,ER\right\}$ groups. In both models, the random intercepts for *Participant* captured the differences across the participants within a group, and the random intercepts for *Stimuli* accounted for the stimuli-to-stimuli variability within the combined stimulus group (A–E, StimP) and normal group (StimN).

A total of 12 models were created, 6 for each of the fixation duration and 3DFD. All models were fitted using MATLAB’s *fitlme* function with maximum likelihood as the method for estimating the model parameters.

Appendix Mathematical Form of Linear Mixed-Effects Modelling Equations provides details of the mathematical equations corresponding to the R-notation Equations (7) and (8).

#### 2.9.2. Effect Size Computation

For each fixed-effect contrast (N vs. EC, N vs. ER), Cohen’s d was computed as:

(9) $$d=\frac{\beta }{\sigma_{e}}$$

where β is the model-estimated fixed-effect coefficient and $\sigma_{e}$ is the residual standard deviation obtained from the mixed-effects model. This definition reflects the magnitude of the effect relative to within-subject variability and is appropriate for linear mixed-effects modelling. Effect sizes were interpreted according to established benchmarks: small ($\left|d\right|$ ≈ 0.1–0.3), medium (≈0.3–0.5), and large (>0.5).

#### 2.9.3. False Discovery Rate (FDR) Correction

False discovery rate (FDR) correction was applied to control multiple comparisons within each family of statistical tests. In this context, a family of statistical tests refers to all hypothesis tests conducted for the same outcome variable (e.g., fixation duration or 3DFD) across all stimulus groups (A–E, StimP) combined with group (StimN). No additional multiple-comparison correction beyond FDR correction was applied.

#### 2.9.4. Sample Size of Data

Across all participants and stimuli, the dataset comprises 69 participants, 39 stimuli and 10 fixation locations per participant–stimulus pair. Therefore, the maximum number of fixation-level observations available for the primary analysis was: 69×39×10=26,910 observations. Because separate LMMs were fitted for each pathology stimulus group by comparing that group with StimN, the number of observations entering each model varied by stimulus group, with the StimP model using the maximum observation count and the pathology-specific models using fewer observations. Because these observations were repeated within participants and stimuli, they were not treated as statistically independent observations. Instead, participant and stimulus were included as random intercepts in the mixed-effects models. This modelling framework allowed the repeated-measures structure of the dataset to be used while accounting for within-participant and within-stimulus dependency. It should be noted that the fixation locations where 3DFD values were ≤1.7 were not included in our LMM analysis.

#### 2.9.5. Software for Statistical Data Processing

Appendix A.4.1 provides details of the software used.

### 2.10. Non-Linear Modelling Using Supervised Machine Learning Methods

To test hypothesis H3, we examined whether joint non-linear modelling of geometric complexity (3DFD) and fixation duration improves discrimination between naïve and expertise groups compared with models based on either feature alone. Supervised machine-learning classifiers were therefore trained using three feature configurations: fixation duration only, 3DFD only, and a combined feature set incorporating both measures.

#### 2.10.1. Feature Engineering for Machine Learning Classifiers

The features were created in three main steps. Firstly, all fixation durations for a given image–participant pair were sorted in descending order, and the 10 longest fixation durations were selected. The 10 longest durations generally represented most of the temporal engagement of participants with a stimulus, so it was empirically chosen and fixed across all the stimuli. For each of these 10 fixations, their fixation coordinates were taken, and the corresponding 3DFD values were retrieved from the precomputed 3DFD map at those coordinates. Three sets of features were then created as shown in Table 3.

The choice of 10 features is consistent with the number of data points taken from each of the participant–stimulus data points in mixed-effects modelling ($M_{P}^{s}$ = 10).

#### 2.10.2. Machine Learning Classifiers

Two supervised machine-learning classifiers were trained to classify the visual expertise of participants into naïve (N) or expert consultant (EC), naïve (N) or expert registrar (ER), expert consultant (EC) or expert registrar (ER). For each of these binary classifiers, two classifiers were developed using Random Forest and Support Vector Machine.

1. Random Forest (RF)

A Random Forest classifier was implemented using the *TreeBagger* algorithm with 200 trees (*TreeBagger* in MATLAB 2025b). Each tree was grown on a bootstrap-resampled subset of the training data. At each split, a random subset of predictors was sampled automatically by MATLAB’s internal heuristic for classification forests. Trees were grown to full depth without pruning to maximize ensemble diversity, and class predictions were obtained through majority voting across trees. This configuration has been widely used by other researchers in biomedical pattern-recognition tasks due to its merits, such as its effectiveness in handling non-linear decision boundaries, robustness to noise and reduction in the risk of overfitting by averaging across diverse trees [34,35].

2. Support Vector Machine (SVM)

A Support Vector Machine classifier with a radial basis function (RBF) kernel (*fitcsvm* in MATLAB 2025b) was trained to model non-linear decision boundaries in the feature space. The features were standardized before training to improve optimization and margin estimation. MATLAB’s internal automatic hyperparameter selection was used for the RBF kernel scale. This kernel-based SVM is well-suited for non-linear classification problems and is consistent with other biomedical modelling studies when dimensionality is moderate [36]. For completeness, we also evaluated a linear-kernel SVM, in addition to the RBF SVM reported in the main analysis.

#### 2.10.3. Software for Machine Learning Classifiers

Appendix A.4.2 provides details of the software used.

#### 2.10.4. 10-Fold Cross-Validation and Performance Evaluation

We used 10-fold cross-validation as the primary method for reporting all performance metrics, including accuracy, sensitivity, specificity, precision, F1 score, and AUC. For this 10-fold cross-validation, the classifier-level dataset was partitioned into 10 folds, with 9 folds used for training and the remaining fold for testing. This process was repeated until each fold had served once as the test set, and the performance metrics from these 10 runs were averaged to obtain a single performance estimate.

All cross-validation partitions were performed at the participant level, ensuring that all participant–stimulus samples from a given participant were assigned exclusively to either the training or testing set within a fold, thereby preventing subject-level data leakage. Because the classification analyses were performed as binary group comparisons, the actual classifier-level sample size varied according to the two groups being compared and the number of stimuli in each stimulus group. The details of the total size of the input dataset for 10-fold subject-wise cross-validation are provided in Appendix D, Table A11 and Table A12. The exact fold size could vary slightly because fold assignment was performed at the participant level. We additionally conducted a sensitivity analysis by repeating the same participant-level cross-validation with *K* ∈ {2, 3, …, 9}, examining the stability of the performance metrics across different choices of fold sizes.

## 3. Results

We report inferential (linear mixed-effects) and predictive (machine learning) findings addressing H1–H3 across all stimulus sets. Throughout this section, we adopt the notation A–E to denote pathology-based stimulus groups: A (tumors); B (cerebrovascular pathologies); C (other pathologies); D (inflammatory changes); E (malformations), as presented in Table 2.

### 3.1. H1: Expertise-Related Differences in Fixation-Duration Behavior

#### 3.1.1. Qualitative Comparison of Fixation-Duration Distributions

**Figure 2:**
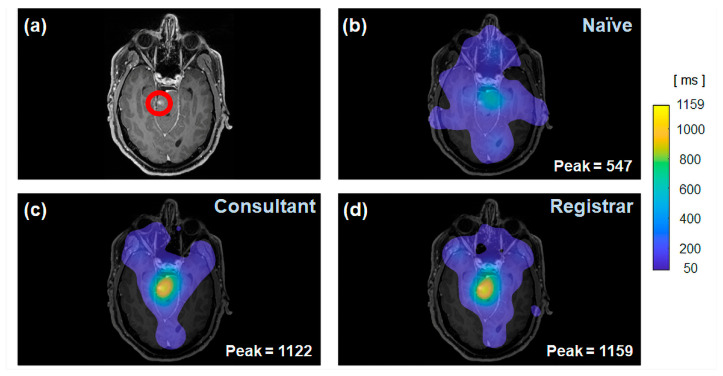
Qualitative comparison of the heatmaps of fixation duration for naïve, consultant and registrar groups. (**a**) One of the MRI structural images taken from stimulus group D—Inflammatory changes, and used as stimuli with pathology (Multiple sclerosis) highlighted in the red circle. (**b**–**d**) Heat maps of fixation duration averaged over all the participants in a group and superimposed on the structural MRI for (**b**) naïve, (**c**) consultant, and (**d**) registrar groups. The fixation duration values below 50 ms are not displayed in (**b**–**d**). Note that the red circle shown in (**a**) is not present in the stimulus when it is viewed by the participants during their eye-tracking data collection session.

**Figure 3:**
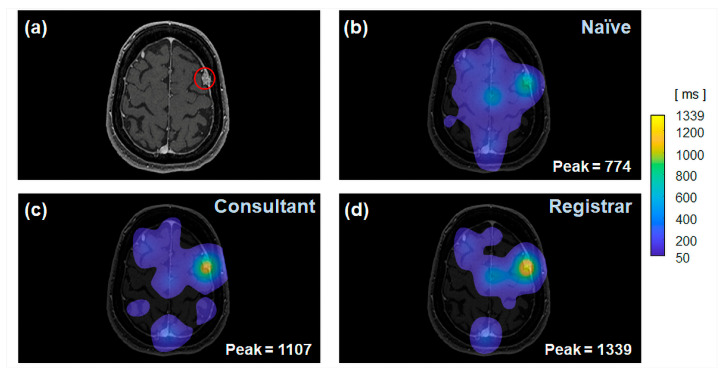
Qualitative comparison of the heatmaps of fixation duration for naïve, consultant and registrar groups. (**a**) One of the MRI structural images taken from stimulus group A—Tumors, and used as stimuli with pathology (Convexity meningioma) highlighted in the red circle. (**b**–**d**) Heat maps of fixation duration averaged over all the participants in a group and superimposed on the structural MRI for (**b**) naïve, (**c**) consultant, and (**d**) registrar groups. The fixation duration values below 50 ms are not displayed in (**b**–**d**). Note that the red circle shown in (**a**) is not present in the stimulus when it is viewed by the participants during their eye-tracking data collection session.

A comparison of the averaged fixation duration based heatmaps (Figure 2 and Figure 3) for naïve, consultant and registrar groups indicates that there are differences in their gaze pattern. The experts (consultant and registrar groups) appear to focus on a pathology for a longer duration than the Naïve participants. The Naïve participants do not appear to fixate on a region for a longer duration; instead, their fixations are dispersed over a wider area on the stimulus (Figure 2b and Figure 3b). The focus of naïve participants appears to be dispersed not only for a smaller-sized pathology in Figure 2b, but also for a relatively larger-sized pathology in Figure 3b.

**Figure 4:**
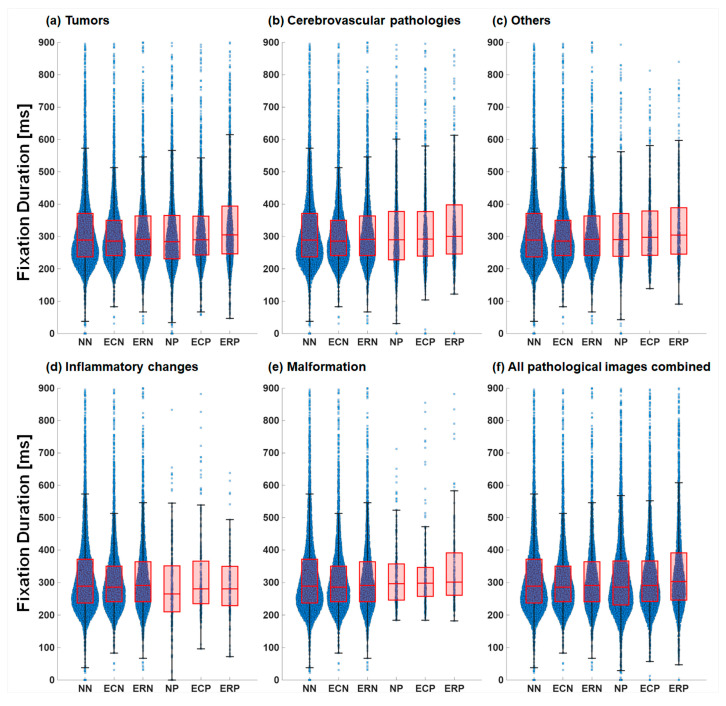
Fixation duration distributions across participant groups for different pathological stimulus categories. Only $M_{P}^{s}$ = 10 maximum fixation durations are taken. Subpanels (**a**–**e**) show tumors, cerebrovascular pathologies, others, inflammatory changes, and malformations, respectively; (**f**) shows all pathological stimuli combined. Boxplots (shown in red color) compare naïve participants viewing normal images (NN), expert consultants viewing normal images (ECN), expert registrars viewing normal images (ERN), and the corresponding groups viewing pathological images (NP, ECP, ERP). Boxes indicate the interquartile range with median values; whiskers denote the non-outlier range. Individual fixation events are overlaid as swarm plots (shown in blue color), where horizontal dispersion reflects the local density of observations along the fixation-duration axis. The *y*-axis is constrained to a range of [0–900] ms, which covers more than the 99th percentile of the *y*-axis values. Note that in each subpanel (**a**–**e**), normal images (StimN) contribute 20 stimuli, whereas the paired pathology category contributes A = 10, B = 4, C = 3, D = 1, E = 1 stimuli (Table 2). As each stimulus contributes its top 10 fixations per participant, the total number of plotted fixation events differs between normal and pathology within a subpanel and influences the density and visual appearance of the plotted observations.

Figure 4 shows boxplots comparing naïve participants viewing normal images (NN), expert consultants viewing normal images (ECN), expert registrars viewing normal images (ERN), and the corresponding groups viewing pathological images (NP, ECP, ERP). Across all stimulus groups in Figure 4, the swarm overlays for naïve participants (NN, NP) show wider horizontal spread, forming broader “violin-like” point clouds. This pattern indicates a greater dispersion of fixation durations relative to the slightly tighter distributions observed in expert groups (ECN, ERN, ECP, ERP). Note that the fixation durations in these boxplots and violin plots were taken from the eye tracking reports (see Appendix A Table A1 for report template), where only data corresponding to $M_{P}^{s}$ = 10 maximum fixation durations were used.

Importantly, within each subpanel (a–e) in Figure 4, the total number of plotted fixation-level observations differs between normal and pathology because the number of stimuli per condition is not the same: the normal (StimN) set contributes 20 images, whereas the paired pathology sets contribute A = 10, B = 4, C = 3, D = 1, and E = 1 images (Table 2). As each stimulus contributes its top 10 fixations per participant, aggregating across unequal numbers of stimuli yields different totals of plotted events within a subpanel. In contrast, panel (f) with “All pathological images combined” pools 20 normal and 19 pathological images, so the total number of plotted events, and thus the overall appearance of the violins, is very similar between conditions.

#### 3.1.2. Quantitative Assessment of Fixation Duration Using Mixed-Effects Modelling

Using normal images and the naïve group (N) as reference categories, the mixed-effects models showed no significant ImageType main effects across stimulus groups A–E or in the combined analysis (all *p_FDR* > 0.05). In contrast, expertise-related effects emerged primarily through Group × ImageType interactions. For expert consultants (EC), EC × ImageType effects were FDR-significant in stimulus groups A (*β* = 19.091, *p_FDR* = 0.004), B (*β* = 25.271, *p_FDR* = 0.006), and StimP (β = 20.464, *p_FDR* <0.001), but not in C, D, or E. For expert registrars (ER), ER × ImageType effects were FDR-significant in stimulus groups A (*β* = 45.649, *p_FDR* < 0.001), B (*β* = 42.304, *p_FDR* < 0.001), C (*β* = 26.567, *p_FDR* = 0.033), E (*β* = 42.393, *p_FDR* = 0.042), and StimP (*β* = 39.733, *p_FDR* < 0.001), but not in D. Cohen’s *d* values indicated small effect sizes for EC interactions (*d* ≈ 0.11–0.15) and consistently larger, but still small effect sizes for ER interactions (*d* ≈ 0.14–0.26), supporting greater reliability of registrar-related effects.

The $M_{P}^{s}$ = 5 maximum fixation-duration results reported in Appendix B Table A2 showed a highly convergent qualitative pattern with the $M_{P}^{s}$ = 10 findings reported in Table 4. As with $M_{P}^{s}$ = 10, ImageType main effects relative to the naïve–normal reference were absent, and fixation-duration differences were primarily expressed through Group × ImageType interactions. The same stimulus groups that showed reliable effects at $M_{P}^{s}$ = 10 (notably A, B, and the aggregated StimP analysis) also exhibited interaction effects at $M_{P}^{s}$ = 5, with additional ER-specific effects observed in C and E, while stimulus group D remained non-significant across both maximum thresholds. Effect size estimates at $M_{P}^{s}$ = 5 mirrored those at $M_{P}^{s}$ = 10, with EC interactions remaining small and ER interactions consistently in the small-to-moderate range, indicating that the fixation-duration findings are stable and reliable across fixation-count thresholds.

### 3.2. H2: Expertise-Related Differences in Structural Complexity (3DFD) at Fixation Locations

#### 3.2.1. Qualitative Comparison of 3DFD Distributions at Fixation Locations

**Figure 5:**
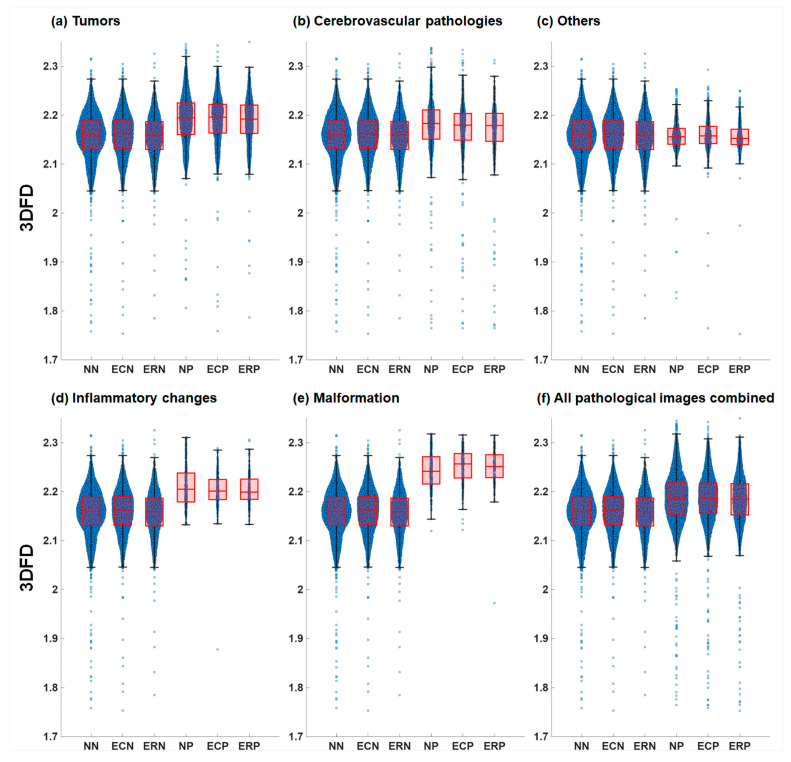
Distributions of local geometric complexity, quantified by the 3DFD, across participant groups for different pathological stimulus categories. The 3DFD values are taken only for the top 10 maximum fixation duration locations. Subpanels (**a**–**e**) correspond to tumors, cerebrovascular pathologies, others, inflammatory changes, and malformations, respectively; (**f**) shows all pathological stimuli combined. Boxplots (shown in red color) compare naïve participants viewing normal images (NN), expert consultants viewing normal images (ECN), expert registrars viewing normal images (ERN), and the corresponding groups viewing pathological images (NP, ECP, ERP). Boxes represent the interquartile range with median values, and whiskers denote the non-outlier range. Individual 3DFD values at fixation locations are overlaid as swarm plots (shown in blue color), where horizontal dispersion reflects the local density of observations along the 3DFD axis. The *y*-axis is constrained to a range of [1.7–2.3], which covers more than 99 percentiles of the *y*-axis values. Note that in each subpanel (**a**–**e**), normal images (StimN) contribute 20 stimuli, whereas the paired pathology category contributes A = 10, B = 4, C = 3, D = 1, E = 1 stimuli (Table 2). As each stimulus contributes its top 10 fixations per participant, the total number of plotted fixation events differs between normal and pathology within a subpanel and influences the density and visual appearance of the plotted observations.

A qualitative comparison of 3DFD distributions is presented in Figure 5. Distributions are shown for naïve participants (NN), expert consultants (ECN) and expert registrars (ERP) when they are viewing normal images and pathological images (NP, ECP, ERP). For the interpretation of violin “fullness,” the aggregation logic and stimulus-count differences described in Section 3.1.1 (regarding Figure 4) also apply to Figure 5. Like Figure 4, the panel (f) in Figure 5 also has normal and pathology stimuli of 20 and 19, respectively.

#### 3.2.2. Quantitative Assessment of 3DFD Using Mixed-Effects Modelling

Using normal images and the naïve group (N) as reference categories, the $M_{P}^{s}$ = 10 mixed-effects models showed no FDR-significant ImageType main effects on 3DFD across stimulus groups A, B, C, D, or in the aggregated analysis (StimP) (all p_FDR ≥ 0.051). A clear exception was observed for stimulus group E (malformations), where a strong positive ImageType main effect was present (*β* = 0.103, *p_FDR* < 0.001, *d* = 0.857), indicating substantially higher 3DFD for pathological relative to normal images within the naïve group. In contrast to fixation-duration results, expertise-related effects on 3DFD were expressed selectively and in a stimulus-dependent manner. For stimulus group B, both EC × ImageType (*β* = −0.031, *p_FDR* < 0.001, *d* = −0.244) and ER × ImageType (*β* = −0.026, *p_FDR* < 0.001, *d* = −0.205) interaction effects were FDR-significant, indicating reduced 3DFD for experts relative to the naïve–normal reference. Conversely, for stimulus group D, strong positive interaction effects were observed for both EC (*β* = 0.078, *p_FDR* < 0.001, *d* = 0.582) and ER (*β* = 0.082, *p_FDR* < 0.001, *d* = 0.612), indicating markedly higher 3DFD values for experts on pathological images relative to naïve participants. No FDR-significant Group × ImageType interactions were observed in stimulus groups A, C, or E, nor in the StimP analysis. Cohen’s *d* values indicate large ImageType effects for E, moderate-to-large interaction effects for stimulus group D, small effects for stimulus group B, and negligible effects elsewhere, highlighting that 3DFD-based expertise effects at $M_{P}^{s}$ = 10 are strongly stimulus-specific.

The $M_{P}^{s}$ = 5 (Appendix B Table A3) 3DFD results reported in Appendix B showed a slightly convergent qualitative pattern with the $M_{P}^{s}$ = 10 findings reported in Table 5. As with $M_{P}^{s}$ = 10, the Group × ImageType interactions remained restricted to the same stimulus categories, with negative expert interactions in stimulus group B and positive expert interactions in stimulus group D, and no reliable interaction effects in A, C, E, or StimP. Cohen’s *d* values at $M_{P}^{s}$ = 5 mirrored those at $M_{P}^{s}$ = 10, with large ImageType effects in E, moderate-to-large interaction effects in D, smaller interaction effects in B, and negligible effects elsewhere, largely supporting the conclusion that the 3DFD findings are robust to the choice of maximum number of fixations and reflect stable, stimulus-dependent differences.

### 3.3. H3: Expertise-Related Differences in Joint Non-Linear Modelling of 3DFD and Fixation Duration

Table 6, Table 7 and Table 8 report 10-fold cross-validation results for the Random Forest classifier. The results are reported as mean ± standard deviation across cross-validation folds. Accuracy is expressed as a percentage, whereas sensitivity, specificity, precision, F1 score, and area under the receiver operating characteristic curve (AUC) are reported on the 0–1 scale. Accuracy and specificity values for the combined feature set are highlighted to summarize the improved overall discrimination and reduced false-positive rates achieved through joint modelling of 3DFD and fixation duration. Sensitivity, specificity, precision, F1, and AUC are defined with respect to the expert group as the positive class.

Across all three binary classification tasks (N vs. EC, N vs. ER, and EC vs. ER), the Random Forest classifiers trained on the combined feature set consistently demonstrated superior overall performance, particularly in terms of accuracy, and generally for specificity as well, across stimulus sets. The fixation-duration-only classifiers generally showed lower discriminative performance, while classifiers trained on 3DFD alone often performed strongly but with greater variability across stimuli. The overall pattern indicates that although geometric complexity provides a substantial discriminative capacity on its own, fixation duration contributes complementary temporal information that enhances classification when jointly modelled. The advantage of the combined feature representation was observed consistently across all stimulus groups (A–E, StimP, StimN). The only exception to this trend was the stimulus group D for N vs. ER and EC vs. ER classifiers, where the 3DFD-only classifier performed slightly better than the combined-feature-set-based classifier.

The SVM classifiers showed a consistent pattern across all three binary tasks. Fixation-duration-only SVM models generally yielded the weakest performance, whereas 3DFD-only models often performed better but with greater variability across stimulus groups. Unlike the Random Forest classifiers, SVMs did not show benefit from combining fixation duration and 3DFD, with combined-feature models often performing poorer than single-feature models. Full numerical results for all stimulus groups are provided in Appendix B Table A4, Table A5, Table A6, Table A7, Table A8 and Table A9 for $M_{P}^{s}$ = 10 longest fixation duration locations and *K* = 10 folds.

Multi-*K* robustness analysis results for *K* ∈ {2, 3, …, 10}, as provided in Appendix B Figure A3 (for $M_{P}^{s}$ = 10 longest fixation duration locations) and A4 (for $M_{P}^{s}$ = 5 longest fixation duration locations) for Random Forest and SVM classifiers, show that accuracy remained broadly consistent across cross-validation settings for Random Forest models. However, the SVM accuracies vary with *K* across all pathology groups: A (tumors), B (cerebrovascular pathologies), C (others), D (inflammatory), E (malformations), and StimP (combined stimuli images), for both $M_{\rho }^{s}=5$ and $M_{\rho }^{s}=10$. Across *K* ∈ {2, 3, …, 10} and stimulus groups, linear-SVM (Appendix B Figure A5) and RBF-SVM (in Appendix B Figure A3) achieved similar accuracies, and both were consistently below Random Forest, particularly for the combined (3DFD + fixation duration) features; thus, including a linear kernel did not alter the conclusion that Fandom Forest provides the strongest and most stable performance.

### 3.4. Linking Mixed-Effects and Machine-Learning Analyses

Across all three binary classification tasks (N vs. EC, N vs. ER, and EC vs. ER), classifiers trained on fixation duration alone generally showed lower performance, while 3DFD-only classifiers often performed strongly but with greater variability across stimuli. This pattern mirrors the mixed-effects findings, where fixation duration and 3DFD exhibited independent but non-identical contributions to group differentiation. Moreover, while the mixed-effects models tested group-level associations separately for fixation duration and 3DFD, the classification analysis tested whether these features, alone or in combination, could discriminate expertise status in unseen participants. Thus, the machine-learning analysis addressed the predictive component of H3, which is not addressed by the LMMs.

## 4. Discussion

We demonstrated that the visual expertise of neurosurgeons could be characterized by jointly analyzing fixation durations derived from eye-tracking data and local image structural complexity at the locations of fixations, during free viewing of brain MRIs. Fixation duration and 3DFD captured complementary aspects of gaze behavior, and their combined consideration provided a more informative description of expertise-related variation than either feature in isolation.

### 4.1. Heat Maps of Fixation Duration

**Figure A2:**
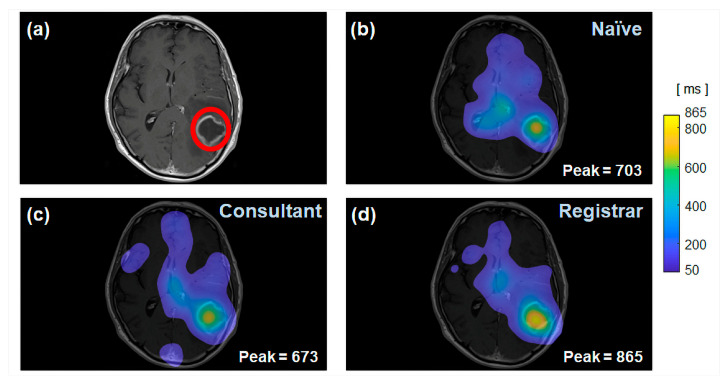
Qualitative comparison of the heatmaps of fixation duration for naïve, consultant and registrar groups. (**a**) One of the MRI structural images taken from stimulus group A – Tumors, and used as stimuli with pathology (Glioblastoma) highlighted in the red circle. (**b**–**d**) Heat maps of fixation duration averaged over all the participants in a group and superimposed on the structural MRI for (**b**) naïve, (**c**) consultant, and (**d**) registrar groups. The fixation duration values below 50 ms are not displayed in (**b**–**d**). Note that the red circle shown in (**a**) is not present in the stimulus when it is viewed by the participants during their eye-tracking data collection session.

The heat maps of fixation durations presented in Figure 2 and Figure 3 and in Appendix B Figure A2 represent the group-level heat maps of fixation durations averaged across all the participants within the group. The qualitative analysis in Figure 2b and Figure 3b indicates that the naïve participants do not focus on a particular region within the stimulus image and instead allocate their attention throughout the image. This is attributed to their lack of knowledge of the domain of stimuli. Experts, on the other hand, are trained in image interpretation, which helps them focus on pathology for a longer duration (Figure 2b and Figure 3b), (Figure 2c and Figure 3c). We emphasize that the gaze pattern is only suggestive of attentional allocation, and we acknowledge that covert attention may not always align fully with gaze position. Notably, this is not general behavior and, in some prominent pathologies, such as the one shown in Appendix B Figure A2b, the allocation of visual attention is less dependent on expertise. In this example, naïve participants could allocate most of their visual attention to pathological regions for similar durations as the experts. Note, however, that the pattern of fixation distributions of naïve participants still shows that they get attracted to the other normal regions of the stimulus image.

### 4.2. Linear Mixed-Effects Modelling

#### 4.2.1. Expertise Effects in Fixation Duration

Consistent with the mixed-effects modelling results (Table 4 and Appendix B Table A2), experts demonstrated longer fixation durations on pathological images relative to naïve participants, with these differences expressed through Group × ImageType interactions rather than uniform main effects, which supports hypothesis H1. When interpreted alongside the qualitative observations reported in Section 4.1, these findings suggest that experts allocate visual attention more selectively, prolonging fixations when clinically relevant abnormalities are present. Rather than reflecting a general tendency toward longer fixations, this pattern indicates a context-dependent modulation of fixation duration, whereby experts engage in sustained visual inspection, specifically when pathology is detected. This targeted prolongation of fixations on pathological images is consistent with established models of visual expertise, which propose that experts are able to suppress irrelevant visual information while dedicating increased attentional resources to diagnostically meaningful regions. In the free-viewing paradigm used in this study, experts appeared to rapidly identify salient pathological features and subsequently maintain fixation on these regions for longer durations, supporting the inference that fixation duration reflects strategic information extraction rather than inefficient search behavior.

Importantly, expertise-related differences in fixation duration were not uniform across all stimulus categories but varied as a function of both image type and observer group (Table 4, Appendix B Table A2). Across pathological stimulus categories, both consultants and registrars exhibited longer fixation durations than naïve participants, irrespective of the number of maximum fixation points considered ($M_{P}^{s}$ = 5 or $M_{P}^{s}$ = 10). This pattern is consistent with the expectation that when trained observers identify pathology, they engage in more sustained scrutiny of abnormal regions even under unconstrained free-viewing conditions.

Notably, fixation-duration effects were often more pronounced in registrars than in consultants, suggesting that prolonged fixation may reflect an intermediate stage of expertise. One plausible explanation is that registrars, while capable of identifying pathological regions, may rely on extended visual confirmation as part of their interpretative process. With increasing experience, consultants may achieve similar diagnostic outcomes with less prolonged fixation, reflecting greater efficiency in visual processing and decision-making. Under this explanation, elevated fixation duration in registrars may represent a transitional marker of developing expertise, rather than a monotonic indicator of expert performance.

#### 4.2.2. Image Complexity and 3DFD Effects

Higher fractal dimension (FD) values correspond to increased geometric complexity in visual stimuli [37]. The 3DFD analysis revealed stimulus-dependent effects of ImageType, with pathological images exhibiting higher 3DFD values than normal images for specific pathology categories, most prominently for malformations (stimulus group E) and, to a lesser extent, in the aggregated analysis. These findings indicate that certain pathological stimuli are characterized by greater structural irregularity and visual complexity, and that 3DFD is sensitive to these differences when they are present. Importantly, the absence of uniform ImageType effects across all stimulus groups suggests that image complexity varies substantially across pathological types, rather than reflecting a global distinction between pathological and normal images. This stimulus-specific pattern is consistent with our hypothesis H2, which predicts that expertise-related 3DFD differences should emerge only for pathology categories where local structural complexity is diagnostically relevant.

Because 3DFD values were computed at fixation locations, these effects reflect the complexity of image regions that observers actively sampled. Importantly, expertise-related differences in 3DFD were not uniform across stimulus groups. Significant Group × ImageType interaction effects were observed selectively, with experts exhibiting significantly lower 3DFD values than naïve participants in stimulus group B, suggesting reduced sampling of highly complex regions in this pathology category of cerebrovascular pathologies. In contrast, experts showed significantly higher 3DFD values in stimulus group D, indicating that expert observers selectively engaged with more structurally complex regions when such complexity was diagnostically relevant in this pathology category of inflammatory changes. No reliable expertise-related modulation of 3DFD was observed in stimulus groups A, C, E, or in the aggregated analysis.

These stimulus-dependent effects indicate that experts are not simply less influenced by visual complexity but rather modulate their sampling of complex image regions based on diagnostic relevance. In some cases, experts deprioritize visually irregular regions that lack diagnostic value, whereas in others they deliberately focus on structurally complex areas that are clinically informative. This pattern aligns with models of perceptual expertise, suggesting that experts rely less on low-level salience and more on semantic knowledge, structured search strategies, and internal templates when interpreting medical images [38,39].

#### 4.2.3. Integration of Complexity and Fixation Behavior

Taken together, the fixation duration and 3DFD results reveal a complementary pattern of attentional control. Naïve participants tend to be attracted to visually complex regions and distribute fixations more broadly, whereas experts exhibit greater selectivity in where they fixate, coupled with longer fixation durations when pathology is identified. Thus, expertise is characterized not by increased sensitivity to visual complexity per se, but by strategic allocation of attention based on diagnostic relevance.

The relatively small effect sizes observed in the 3DFD models, contrasted with larger and more consistent effects in fixation duration, suggest that 3DFD primarily captures image-driven differences, whereas fixation duration more strongly reflects observer-driven behavioral modulation. This distinction is important, as it indicates that 3DFD and fixation duration quantify independent yet complementary dimensions of visual expertise: one reflecting the property of the visual stimulus at sampled locations, and the other reflecting how observers engage with diagnostically meaningful information over time.

#### 4.2.4. Comparison with Prior Studies in Fixation Duration and Geometric Complexity

A prior study by Suman et al. [10] reported expertise-related differences in visual search behavior during interpretation of pathological medical images, broadly consistent with the fixation-duration effects observed in the present study. However, the expert cohort in their study comprised radiologists from a range of subspecialties (e.g., neuroradiology, head and neck, chest, musculoskeletal, and interventional radiology), with only 4 out of 31 experts being neurosurgeons. As a result, their findings reflect general radiological expertise rather than expertise specific to neurosurgical image interpretation. In contrast, the present study explicitly focuses on neurosurgeons, allowing the reported fixation-duration patterns to be interpreted within a domain-specific clinical context.

Importantly, Suman et al. [10] quantified expertise effects using fractal dimension analysis of scanpaths, reporting higher geometric complexity of scanpaths when experts viewed pathological stimuli. While this approach captures differences in eye-movement organization, it does not directly relate scanpath complexity to the geometric complexity of the underlying image content. In contrast, the current study applied a 3D fractal dimension analysis to the stimulus images themselves, evaluated at fixation locations. This approach enables a more direct examination of how observers sample image regions of varying structural complexity, thereby linking fixation behavior to the geometric properties of the visual stimulus. Together, these methodological differences highlight that scanpath-based and image-based fractal analyses capture complementary aspects of visual expertise, with the present findings extending prior work by explicitly relating gaze behavior to the structural complexity of pathological image regions.

### 4.3. Non-Linear Modelling with Supervised Machine Learning

In addition to inferential statistical analysis, machine learning classifiers were employed to evaluate whether the identified features can generalize to the prediction of unseen participants. While statistical models establish group-level differences, predictive modelling provides a complementary assessment of the discriminative utility and practical applicability of these features in real-world scenarios such as developing “expert-like” systems for assessing visual expertise.

It is important to clarify that the role of machine learning in this study is not to replace statistical inference, but to complement it. While linear mixed-effects models establish the presence and nature of expertise-related differences, machine learning evaluates whether these differences are sufficiently robust to enable accurate prediction at the individual participant level. This unified analytical framework provides both explanatory and predictive validation of the proposed features.

#### 4.3.1. Joint Modelling of Complexity Using 3DFD and Fixation Duration

The advantage of the combined feature representation was observed consistently across stimulus sets, including both pathology-specific and aggregated pathological image groups (A–E, StimP) as well as in the normal stimuli group StimN. The higher accuracy and specificity in the combined-feature classifiers achieved more reliable overall correctness while reducing false-positive classifications. This indicates that our joint modelling of the two spatial event-level categories of features, the fixation duration and spatial image complexity, provides a more complete characterization of visual expertise than either feature alone. The machine-learning findings support our hypothesis H3 by demonstrating that joint non-linear modelling of the spatial image complexity and the temporal allocation of visual attention yields the most robust and consistent differentiation between expertise groups.

**Figure A3:**
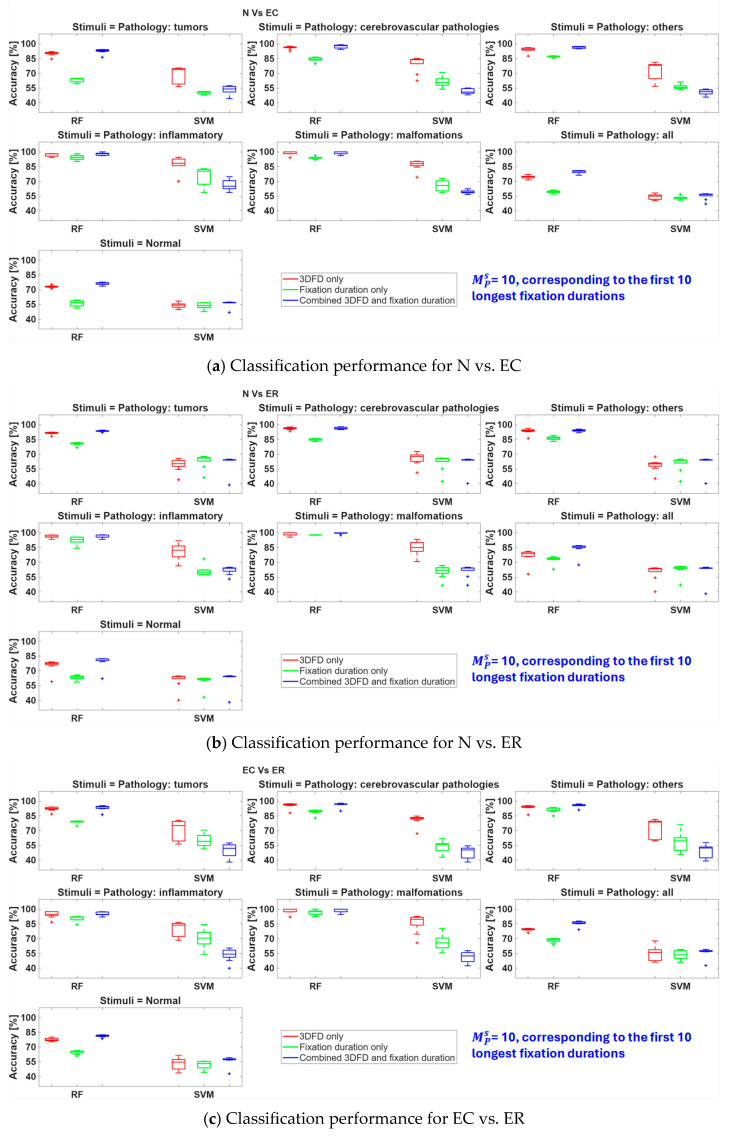
Classification performance for (**a**) N vs. EC, (**b**) N vs. ER, and (**c**) EC vs. ER, using Random Forest (RF) and SVM classifiers across stimulus sets. Panels correspond to stimulus sets A (tumors), B (cerebrovascular pathologies), C (others), D (inflammatory), E (malformations), StimP (all pathologies taken together), and StimN (normal stimuli). For each stimulus set, classifier performance is shown for fixation duration only, 3DFD only, and the combined feature representation. Each box plot in the panels corresponds to the distributions of classifier accuracies for *K* = [2,3,4,5,6,7,8,9,10] in the *K*-fold cross-validation. For these results, we chose the $M_{P}^{s}$ = 10, which corresponds to the first 10 maximum fixation durations for each stimulus for each participant. RBF kernel is used in SVM.

**Figure A4:**
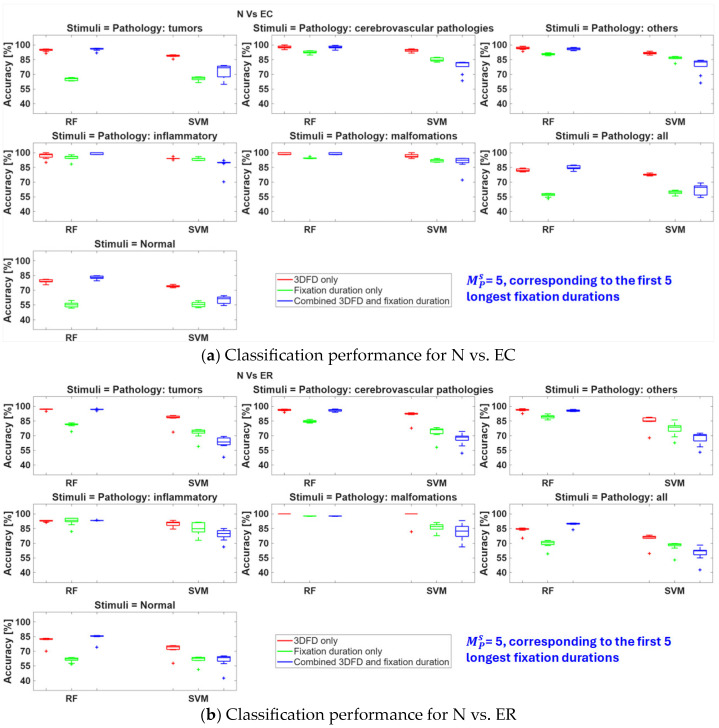

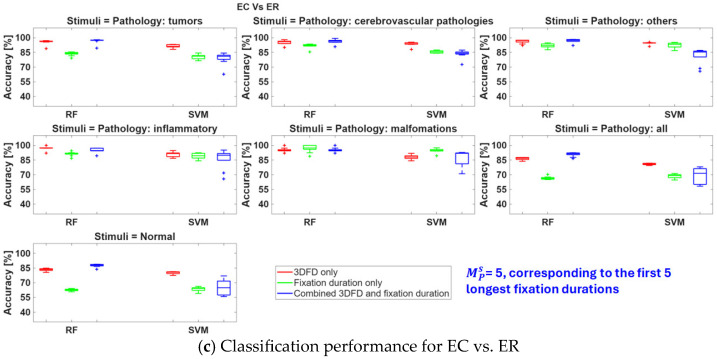
Classification performance for (**a**) N vs. EC, (**b**) N vs. ER, and (**c**) EC vs. ER, using Random Forest (RF) and SVM classifiers across stimulus sets, when the data corresponding to the first 5 longest fixation durations are used. Panels correspond to stimulus sets A(tumors), B (cerebrovascular pathologies), C (others), D (inflammatory), E (malformations), StimP (all pathologies taken together), and StimN (normal stimuli). For each stimulus set, classifier performance is shown for fixation duration only, 3DFD only, and the combined feature representation. Each box plot in the panels corresponds to the distributions of classifier accuracies for *K* = [2,3,4,5,6,7,8,9,10] in the *K*-fold cross-validation. For these results, we chose the $M_{P}^{s}$ = 5, which corresponds to the first 5 maximum fixation durations for each stimulus for each participant. RBF kernel is used in SVM.

**Figure A5:**
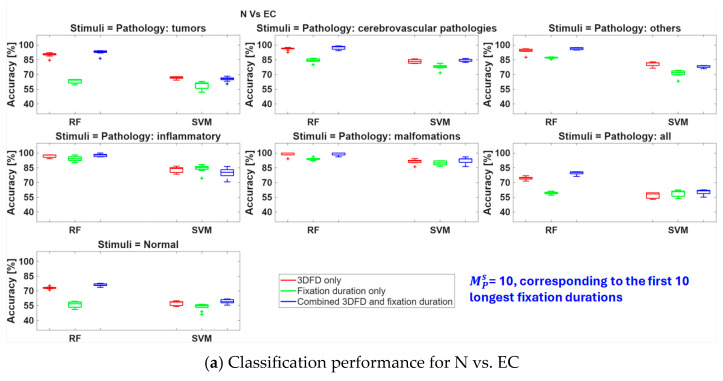

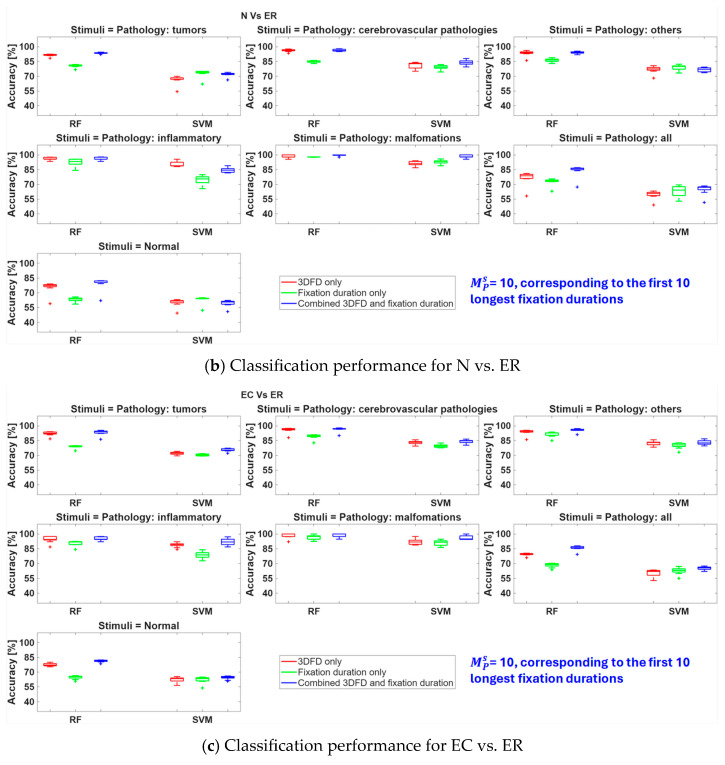
Classification performance for (**a**) N vs. EC, (**b**) N vs. ER, and (**c**) EC vs. ER, using Random Forest (RF) and SVM classifiers across stimulus sets, when the first 10 longest fixation durations are used. Panels correspond to stimulus sets A (tumors), B (cerebrovascular pathologies), C (others), D (inflammatory), E (malformations), StimP (all pathologies taken together), and StimN (normal stimuli). For each stimulus set, classifier performance is shown for fixation duration only, 3DFD only, and the combined feature representation. Each box plot in the panels corresponds to the distributions of classifier accuracies for *K* = [2,3,4,5,6,7,8,9,10] in the *K*-fold cross-validation. For these results, we chose the $M_{P}^{s}$ = 10, which corresponds to the first 10 maximum fixation durations for each stimulus for each participant. A linear kernel is used in SVM.

Although SVM performance gains were less pronounced for the combined feature set (Appendix B Figure A3, Figure A4 and Figure A5, Table A4, Table A5 and Table A6), this does not contradict the combined-feature advantage, as SVMs optimize global decision margins and may not fully exploit conditional feature interactions that are naturally captured by tree-based models such as Random Forest. This is true regardless of whether the SVM kernel is linear or RBF.

We assert that the aim of the machine learning analysis in our study was not to optimize or compare classifier architectures, but to assess whether joint modelling of 3DFD and fixation duration improves group discrimination relative to single-feature representations. Accordingly, conclusions regarding feature complementarity are based on consistent performance gains observed across stimulus sets and supported by robust classifiers (e.g., Random Forest), rather than on the absolute performance of any single algorithm.

#### 4.3.2. 10-Fold Cross-Validation and Performance Evaluation of Classifiers

Our primary evaluation of classifier performance was based on 10-fold cross-validation. Despite this being the standard practice, to ensure that our findings were not inadvertently dependent on our choice of *K* = 10, we additionally conducted a robustness analysis by repeating the same participant-level cross-validation with *K* ∈ {2, 3, …, 9}. The results from this sensitivity check confirmed that performance remained broadly stable across different fold sizes (see Appendix B Figure A3, Figure A4 and Figure A5), supporting the generalizability of the reported results and providing a comprehensive assessment of the discriminative potential of the 3DFD and fixation-duration features.

We adopted a participant-level cross-validation strategy to strengthen the validity of our evaluation. By ensuring that all observations from a single participant were confined to either the training or testing set within each fold, the analysis avoided subject-level data leakage arising from repeated observations per participant. As a result, the reported classification metrics provide a more rigorous assessment of model generalizability, as performance estimates reflect the ability to classify unseen participants rather than repeated samples from the same individual.

#### 4.3.3. Choosing 10 Longest Fixation-Duration Locations

The 10 longest fixation-duration locations were selected to capture fixation events most likely to reflect sustained attentional engagement with the stimulus. This threshold provided a consistent participant–stimulus feature representation for both mixed-effects modelling and machine-learning analysis. To assess whether the findings depended on this threshold, we repeated the main analyses using the top five fixation-duration locations. The broadly similar results obtained for $M_{P}^{s}$ = 10 and $M_{P}^{s}$ = 5 indicate that the principal findings were robust to the selected fixation-count threshold.

### 4.4. Heterogeneity in Brain MR Images and Formation of Stimulus Groups

The decision to divide the stimuli into multiple groups (A–E, StimP, StimN) was driven by the inherent heterogeneity of pathological brain MR images. Different pathology types, such as tumors, cerebrovascular pathologies, inflammatory changes, and malformations, often present with distinct structural characteristics, making it unlikely that a single aggregated pathology group would capture these meaningful differences. Without this grouping, clinically relevant variability could have been obscured, potentially masking how expertise interacts with specific pathology types and limiting our ability to examine how these differences shape expert visual strategies. It is also important to acknowledge that this study did not account for heterogeneity arising from different imaging planes (e.g., axial, sagittal, coronal), anatomical regions, or acquisition conditions within both normal and pathological images. These factors may further influence gaze behavior and complexity sampling but were beyond the scope of the current analysis. Future work that systematically examines these additional sources of heterogeneity may provide deeper insights into how experts adapt their strategies across diverse imaging contexts.

### 4.5. Treating Imbalance in the Three Participant Groups and Stimuli Groups

The participant groups in this study were not perfectly balanced, with fewer expert registrars ER (*N_p_* = 16) than naïve observers N (*N_p_* = 29) or consultant neurosurgeons EC (*N_p_* = 24). This imbalance is not uncommon in medical and surgical eye-tracking studies, where recruitment of specialist clinicians is often constrained. For example, Li et al. [19] studied simulated surgical training using 8 experts and 6 novices; Wilson et al. [21] studied laparoscopic expertise using 8 experienced surgeons and 6 novices; and Dalveren and Cagiltay [22] studied eye movements in surgical residents using 14 novices and 9 intermediates. These studies illustrate the practical recruitment constraints commonly encountered when studying expert medical populations.

Acknowledging the participant imbalance, we addressed it analytically by adopting linear mixed-effects models rather than simple group-wise comparisons for analysis. Adopting this modelling approach explicitly accounted for between-participant and between-stimulus variability. Specifically, our models included random intercepts for Subject and StimuliID, thereby preventing observations from the same participant or stimulus from being treated as statistically independent. This is preferable to conventional ANOVA-based approaches, which are less flexible for unbalanced hierarchical eye-tracking data. Our approach is consistent with a prior study conducted by Suman et al. [10], who used linear mixed-effects models in their eye-tracking study and explicitly noted that these models accommodate unbalanced group sizes. Similarly, other prior eye tracking studies by Chen et al. [40] and Bertram et al. [41] also had imbalanced data, and they all used LMMs.

**Figure A6:**
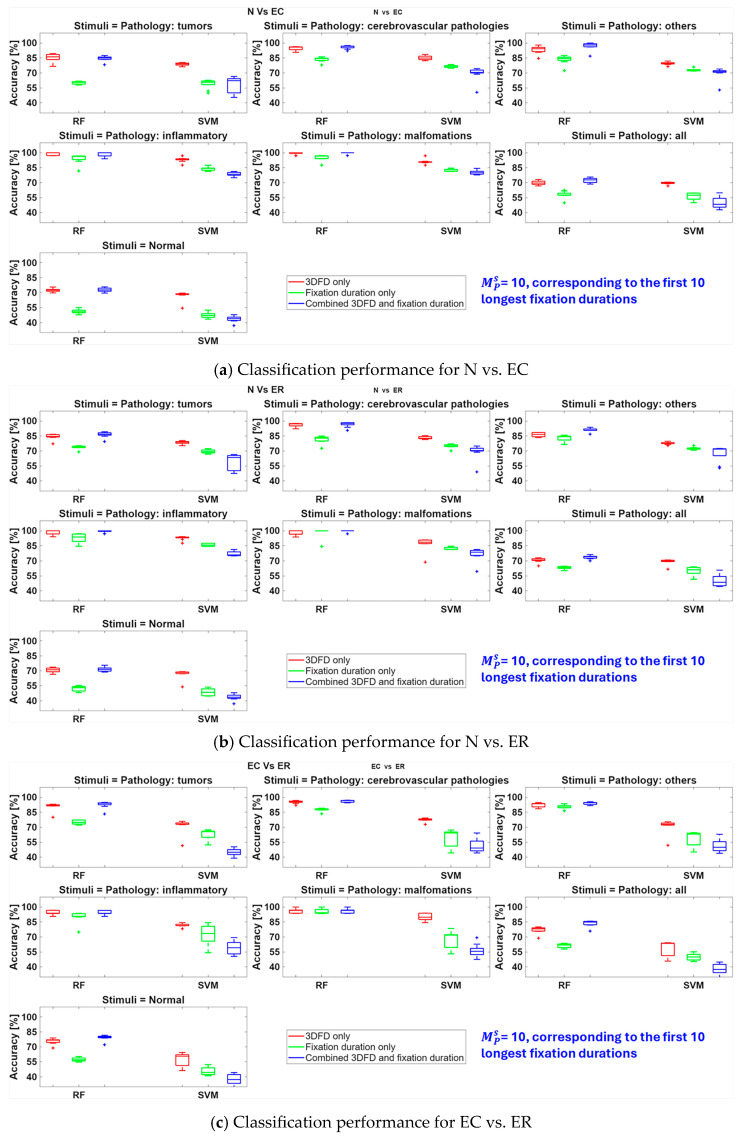
Classification performance for (**a**) N vs. EC, (**b**) N vs. ER, and (**c**) EC vs. ER, using Random Forest (RF) and SVM classifiers across stimulus sets, with an equal number of participants (*N_p_* = 16). Panels correspond to stimulus sets A (tumors), B (cerebrovascular pathologies), C (others), D (inflammatory), E (malformations), StimP (all pathologies taken together), and StimN (normal stimuli). For each stimulus set, classifier performance is shown for fixation duration only, 3DFD only, and the combined feature representation. Each box plot in the panels corresponds to the distributions of classifier accuracies for *K* = [2,3,4,5,6,7,8,9,10] in the *K*-fold cross-validation. For these results, we chose the $M_{P}^{s}$ = 10, which corresponds to the first 10 maximum fixation durations for each stimulus for each participant. RBF kernel is used in SVM.

We also acknowledge that participant-group imbalance can influence machine-learning classifiers because the participant–stimulus-level samples inherit the unequal group sizes. Unlike LMMs, Random Forest and SVM classifiers do not automatically remove class imbalance unless explicit reweighting or resampling is applied. Therefore, we do not claim that the classifiers corrected class imbalance. Instead, we reduced the risk of biased interpretation by using participant-level cross-validation and by reporting multiple class-sensitive performance metrics, including sensitivity, specificity, precision, F1 score, and AUC, in addition to accuracy. These metrics allow classifier performance to be evaluated beyond overall accuracy and help identify whether classification performance is driven disproportionately by the majority class. Noting this, a comparison of our overall results with the results from a subset of our participant data where balancing of groups was maintained (Appendix B Figure A6, Table A7, Table A8 and Table A9) confirmed that the conclusions are still the same, i.e., the performance of the combined feature set is higher or closer to the 3DFD feature set. 

Notably, the number of stimuli also varied across pathology groups (A = 10, B = 4, C = 3, D = 1, E = 1), reflecting the availability of clinically representative pathological brain MRI cases during our study design. It should be noted that separate mixed-effects models were fitted for each pathology stimulus group by comparing that pathology group with the normal stimulus group. Therefore, although the pathology stimulus groups differed in size, each mixed-effects model evaluated one pathology category against the same normal stimulus group, with stimulus-level variability accounted for using random intercepts for StimuliID. Nevertheless, the findings from the pathology categories containing few stimuli, particularly the inflammatory changes (group D) and malformations (group E), should be interpreted with caution and therefore may be treated as stimulus-specific exploratory findings, rather than definitive pathology-category effects.

### 4.6. Selecting Fixation Duration as the Primary Behavioral Feature

Among a range of available eye-tracking metrics, fixation duration was selected as the primary behavioral feature in this study due to its direct interpretability as a proxy for attentional allocation and cognitive processing load [4,9,18,19,23]. Importantly, fixation duration can be co-localized with image-derived features (3DFD) at fixation locations, enabling a direct linkage between gaze behavior and underlying image structure. In contrast, temporal sequence-based metrics such as scanpath similarity or saccade dynamics are less suited for such spatially localized analysis. This careful selection of fixation duration allows for a more interpretable and methodologically coherent integration with local image complexity measures.

### 4.7. Other Methodologies Used for the Characterization of Visual Attention

Alternate methodologies such as fMRI, EEG, and MEG have been employed in prior work to identify the neural substrates of attention and test cognitive theories of attention [1]. However, these approaches require lengthy setup and computationally intensive processing, making them less feasible for studies involving time-constrained clinical experts. Eye-tracking, by contrast, offers a portable, cost-effective, and computationally efficient alternative for studying visual attention. Given the practical challenges of recruiting neurosurgeons with demanding schedules, using eye-tracking cameras was the most suitable choice for our focused study on the neurosurgery experts.

### 4.8. Data Collection and Sampling Rate

A key strength of this study is the use of a high eye-tracking sampling rate, which is critical for accurate fixation detection and feature computation. Sampling rate directly influences the precision of fixation-based measures, as lower rates can introduce temporal discretization errors and compromise the reliability of derived metrics such as the number of fixation points [42]. Different studies have used different sampling rates of eye-trackers for data collection. Suman et al. [10] used a sampling rate of 250 Hz for their eye-tracking data collection. In their eye-tracking study with neuroradiologists, Crowe et al. [16] used Eye-Link 1000 (SR Research, Mississauga, ON, Canada), which had a high-sampling rate of 1000 Hz. Like Crowe et al. [16], our study also adopted a high-sampling rate of 1000 Hz. This choice ensures superior temporal resolution and stability in our fixation-duration-based analyses.

Importantly, our eye-tracker combined this high sampling rate with portability, enabling data collection in diverse environments, including laboratory settings and external venues such as conference desk spaces. This methodological advantage allowed us to maintain data quality without sacrificing logistical flexibility, a critical consideration when working with time-constrained neurosurgical experts. By reducing temporal discretization error in fixation detection, our approach strengthens the robustness and generalizability of the reported findings [10,22,23,42].

### 4.9. Translational Implications for Developing Expert-like Systems Based on Neurosurgical Visual Expertise 

A central motivation of this study was to identify neurosurgery-specific gaze behaviors that could inform the development of computational systems capable of exhibiting “expert-like” visual strategies. To make this link explicit, we synthesize here the most salient empirical findings and outline how each can be operationalized in the design of intelligent models for neurosurgical image interpretation.

First, use expert fixation duration as a behavior-grounded supervisory signal to train attention: models should up-weight features from regions analogous to those attracting experts’ longest fixations when pathology is present, operationalizing the observed Group × ImageType effect where experts dwell longer on abnormal images. Practically, precompute a 3DFD map for each image, extract features at the top-$M_{P}^{s}$ fixation locations (5–10) to capture the most informative scrutiny, and associate these with patch embeddings during training; this retains fidelity to our data pipeline while remaining computationally tractable.

Second, design models to fuse temporal allocation and structural complexity, because these signals are complementary and improve discrimination beyond either alone. Concretely, adopt dual-branch architectures (image branch + fixation/3DFD branch) with a fusion or gating layer that learns non-linear interactions; our Random Forest classifiers showed consistently higher accuracy and specificity with the combined feature set across tasks and stimulus groups, justifying this integration.

Finally, make attention pathology-aware: learn category-specific complexity priors so that 3DFD influences attention only when diagnostically relevant (e.g., higher weighting for inflammatory changes, lower for certain cerebrovascular cases), reflecting the stimulus-dependent expertise effects in our 3DFD analyses. Together, these steps translate our empirical markers of selective fixation prolongation, complexity-dependent sampling, and their synergy into concrete modelling choices for systems that attend and prioritize like neurosurgical experts.

As an alternative expert-like multi-agent (“mixture-of-expertise”) architecture, researchers can train stimulus-specific classifiers aligned with our pathology groupings (A–E, e.g., tumors, cerebrovascular, others, inflammatory, malformations) and then weight or aggregate their outputs into a final expertise score for the observer. This design is directly supported by our results: per-group Random Forest classifiers routinely achieve higher performance than models trained on the combined “StimP” set (e.g., N vs. EC: group A/B/C accuracies ≈ 94–99% vs. 80.9% on StimP; N vs. ER: group A/B/C ≈ 93–96% vs. 87.1% on StimP; EC vs. ER: group A/B/C ≈ 95–97% vs. 87.4% on StimP), indicating that combining heterogeneous stimuli dampens discrimination, whereas category-specific specialization preserves expert-like signals. A multi-agent system can therefore mirror a neurosurgeon’s pathology-dependent strategies by letting each agent specialize on its image type and fusing their calibrated outputs (e.g., via learned weights or Bayesian ensembling) into a single, robust expertise estimate.

As a general note, our framework is designed to classify the observer’s level of visual expertise (e.g., N vs. EC/ER), and not to classify images as normal versus pathological. Any model derived from this work should therefore report expertise level or expert-likeness of viewing behavior, rather than diagnostic labels for the input image. These translational directions could be incorporated into future neurosurgical training simulators by providing objective feedback on whether a trainee’s fixation-duration patterns and fixation-linked complexity sampling resemble those of more experienced neurosurgeons. In this context, the proposed framework may support observer-level assessment of visual expertise rather than image-level diagnostic classification.

### 4.10. Limitations and Future Work

Although the study demonstrates consistent methodological implementation and clear behavioral patterns, several limitations warrant consideration. First, the 3DFD measure captures local image complexity but does not encode higher-level structural or anatomical relevance. Incorporating multi-scale fractal features or complementary descriptors, such as entropy-based measures, may further strengthen the characterization of visual behavior. Moreover, because 3DFD values were computed only at fixation locations rather than across the full image, the reported ImageType and Group × ImageType effects should be interpreted as reflecting differences in the structural complexity of sampled regions, rather than as global measures of image complexity. Second, the expert consultant (EC) and expert registrar (ER) groups differ in experience level, which may give rise to distinct trajectories of search efficiency and attentional control that are not fully captured in the present cross-sectional design. Longitudinal tracking of surgical trainees would enable a more direct examination of how fixation behavior and complexity sampling evolve with increasing expertise.

Future work, including integrating temporal scanpath measures, such as fixation transitions, sequence structure, and gaze entropy, could provide a more comprehensive account of expertise by jointly modelling where observers look, for how long, and in what order. Furthermore, the integrated spatial–temporal framework developed in our study could benefit and extend emerging machine-learning approaches that leverage expert gaze for adaptive or explainable decision-making, such as eye-guided multimodal fusion frameworks [43] that fuse imaging with expert fixation maps to improve interpretability and reliability, and machine-learning systems [44] designed to discriminate radiologists’ experience levels using spatiotemporal eye-tracking encodings.

Future work may also extend this framework to volumetric MRI datasets to examine whether fixation-linked complexity measures derived from full 3D anatomical reconstructions provide additional information beyond the 2D slice-based analysis used here. Such work should also evaluate how the number of MRI slices/segments, slice thickness, inter-slice spacing, and anatomical coverage influence volumetric 3DFD estimation and its usefulness for neurosurgical visualization and training.

Future studies could further extend this framework to immersive virtual-reality environments by visualizing healthy and pathological volumetric MRI data in 3D using head-mounted displays with integrated eye tracking. Such a framework could enable fixation-linked analysis of how neurosurgeons inspect anatomical and pathological structures from multiple viewpoints and may provide additional insight into visual expertise during 3D neurosurgical image interpretation and training.

Also, while the mixed-effects framework accounts for the imbalance in stimuli, findings for smaller stimulus groups, such as groups D (Inflammatory changes) and E (Malformations), should be validated in future studies with larger stimulus datasets.

In addition, future work could incorporate model-explainability analyses such as Gini index, Boruta, or SHAP to quantify the contribution of individual fixation- and complexity-based features to classifier decisions, thereby providing deeper insight into feature-level relevance within expert-like models. Finally, future work should also evaluate the applicability of the translational implications of the integrated spatial–temporal framework developed in this study to other medical imaging domains such as CT, angiography, ultrasound, and other clinical specialties such as ophthalmology, ENT and radiology, where visual expertise may depend on different image structures and task demands.

## 5. Conclusions

The convergence of mixed-effects modelling and machine-learning analyses indicates that fixation duration and geometric complexity (3DFD) capture complementary aspects of gaze behavior associated with visual expertise. While 3DFD reflects the structural properties of image regions sampled during viewing, fixation duration indexes the temporal allocation of attention to diagnostically relevant areas. Importantly, expertise-related differences were most consistently characterized when these spatial and temporal features were considered jointly rather than in isolation. Quantitatively, joint modelling of fixation duration and 3DFD yielded high discriminative performance, with supervised Random Forest classifiers achieving 93–100% accuracy across pathology-specific stimulus groups and approximately 80–87% accuracy for the aggregated stimuli dataset. The agreement between inferential mixed-effects results and predictive classification performance supports an integrated spatial–temporal feature framework as a principled approach for characterizing visual expertise in medical image interpretation.

## Figures and Tables

**Table 1:** Summary of the participant groups, their age, gender and number in each group.

Participant Group Label	Group Name	Number of Participants	Age Range (Years)	Gender (Males)	Gender (Females)	Gender (Prefer not to Say)
N	Naïve	29	23–52	13	16	-
EC	Expert consultants	24	29–61	18	5	1
ER	Expert registrars	16	26–42	10	6	-

**Table 2:** Summary of the stimulus groups, types of MR brain images within each group and their number.

Stimuli Group Label	Image Type	Stimuli Group (Based on Type of Images)	Number of Stimuli
A	Pathological	Tumors	10
B	Pathological	Cerebrovascular pathologies	4
C	Pathological	Others	3
D	Pathological	Inflammatory changes	1
E	Pathological	Malformations	1
StimP	Pathological	All pathological MR images combined	19
StimN	Normal	Normal MR images	20

**Table 3:** Feature sets used in classifiers.

Feature Set	Number of Features
3DFD only	10
Fixation duration only	10
Combined 3DFD and fixation duration	20

**Table 4:** Fixed-Effects Results for Fixation Duration × ImageType across stimulus groups (A–E, and StimP). We chose the $M_{P}^{s}$ = 10, which corresponds to the first 10 maximum fixation durations for each stimulus for each participant.

Stimulus Group	Predictor	β	SE	t	df	*p*	*p_FDR*	Cohen’s d
A	ImageType (tumors)	−8.833	5.675	−1.56	20,094	0.12	0.144	−0.049
A	EC × ImageType (tumors)	19.091	6.213	3.07	20,094	**0.000**	0.004 **	0.106
A	ER × ImageType (tumors)	45.649	6.587	6.93	20,094	**0.000**	0.000 ***	0.244
B	ImageType (cerebrovascular pathologies)	−5.700	8.004	−0.71	16,074	0.476	0.476	−0.033
B	EC × ImageType (cerebrovascular pathologies)	25.271	8.403	3.01	16,074	**0.003**	0.006 **	0.147
B	ER × ImageType (cerebrovascular pathologies)	42.304	9.256	4.57	16,074	**0.000**	0.000 ***	0.247
C	ImageType (others)	0.982	9.542	0.10	15,404	0.918	0.918	0.006
C	EC × ImageType (others)	18.864	9.492	1.99	15,404	**0.047**	0.094	0.110
C	ER × ImageType (others)	26.567	10.455	2.54	15,404	**0.011**	0.033 *	0.155
D	ImageType (inflammatory)	−6.228	14.265	−0.44	14,064	0.662	0.662	−0.035
D	EC × ImageType (inflammatory)	22.526	16.334	1.38	14,064	0.168	0.252	0.126
D	ER × ImageType (inflammatory)	25.540	17.992	1.42	14,064	0.156	0.252	0.143
E	ImageType (malfomations)	−3.294	14.055	−0.23	14,064	0.815	0.815	−0.019
E	EC × ImageType (malfomations)	17.696	15.584	1.14	14,064	0.256	0.384	0.104
E	ER × ImageType (malfomations)	42.393	17.166	2.47	14,064	**0.014**	0.042 *	0.249
StimP	ImageType (all pathologies)	−6.195	5.021	−1.2	26,124	0.22	0.26	−0.034
StimP	EC × ImageType (all pathologies)	20.464	5.160	3.89	26,124	**0.000**	0.000 ***	0.112
StimP	ER × ImageType (all pathologies)	39.733	5.683	6.86	26,124	**0.000**	0.000 ***	0.216

Note. Fixed-effects estimates are from linear mixed-effects models of the form *Fixation Duration ~ Group* × *ImageType* + (1*|Subject*) + (1*|StimuliID*). The naïve group (N) served as the reference category, and normal images served as the reference level for ImageType. *β* = Fixation duration estimate; *d* = Cohen’s d. Significant *p* and *p_FDR* values (*p*/*p_FDR* < 0.05) are shown in bold. Significance levels on FDR-corrected *p*-values (*p_FDR*) are marked as: *p_FDR* < 0.05 (*), *p_FDR* < 0.01 (**), *p_FDR* < 0.001 (***).

**Table 5:** Fixed-Effects Results for 3DFD × ImageType across stimulus groups (A–E, and StimP). We chose the $M_{P}^{s}$ = 10, which corresponds to the first 10 maximum fixation durations for each stimulus for each participant.

Stimulus Group	Predictor	β	SE	t	df	*p*	*p_FDR*	Cohen’s d
A	ImageType (tumors)	0.022	0.011	2.12	20,094	**0.034**	0.102	0.161
A	EC × ImageType (tumors)	0.009	0.005	1.86	20,094	0.062	0.117	0.066
A	ER × ImageType (tumors)	0.009	0.005	1.76	20,094	0.078	0.117	0.066
B	ImageType (cerebrovascular pathologies)	0.032	0.015	2.12	16,074	0.034	0.051	0.252
B	EC × ImageType (cerebrovascular pathologies)	−0.031	0.006	−5.01	16,074	**0.000**	0.000 ***	−0.244
B	ER × ImageType (cerebrovascular pathologies)	−0.026	0.007	−3.81	16,074	**0.000**	0.000 ***	−0.205
C	ImageType (others)	−0.007	0.017	−0.41	15,404	0.683	0.683	−0.056
C	EC × ImageType (others)	0.007	0.007	1.02	15,404	0.308	0.462	0.056
C	ER × ImageType (others)	0.006	0.008	0.82	15,404	0.412	0.494	0.048
D	ImageType (inflammatory)	−0.034	0.028	−1.23	14,064	0.219	0.263	−0.254
D	EC × ImageType (inflammatory)	0.078	0.012	6.37	14,064	**0.000**	0.000 ***	0.582
D	ER × ImageType (inflammatory)	0.082	0.014	6.07	14,064	**0.000**	0.000 ***	0.612
E	ImageType (malfomations)	0.103	0.028	3.69	14,064	**0.000**	0.000 ***	0.857
E	EC × ImageType (malfomations)	−0.011	0.011	−1.02	14,064	0.309	0.371	−0.092
E	ER × ImageType (malfomations)	−0.010	0.012	−0.79	14,064	0.429	0.429	−0.083
StimP	ImageType (all pathologies)	0.019	0.009	2.02	26,124	**0.027**	0.081	0.147
StimP	EC × ImageType (all pathologies)	0.004	0.004	0.66	26,124	0.507	0.507	0.021
StimP	ER × ImageType (all pathologies)	0.005	0.004	0.92	26,124	0.359	0.431	0.028

Note. Fixed-effects estimates are from linear mixed-effects models of the form *Mean3DFD ~ Group × ImageType* + (1*|Subject*) + (1*|StimuliID*). The naïve group (N) served as the reference category, and normal images served as the reference level for ImageType. *β* = 3DFD estimate; *d* = Cohen’s d. Significant *p* and *p_FDR* values (*p/p_FDR* < 0.05) are shown in bold. Significance levels on FDR-corrected *p*-values (*p_FDR*) are marked as: *p_FDR* < 0.001 (***).

**Table 6:** Classification performance for N vs. EC Random Forest classifier using $M_{P}^{s}$ = 10 longest fixation duration locations (10-fold cross-validation).

Stimulus Group	Feature Set	Accuracy	Sensitivity	Specificity	Precision	F1	AUC
A	3DFD	91.27 ± 8.38	0.90 ± 0.08	0.93 ± 0.12	0.91 ± 0.15	0.90 ± 0.08	0.97 ± 0.06
A	Fixation Duration	64.43 ± 11.67	0.41 ± 0.16	0.82 ± 0.11	0.60 ± 0.23	0.59 ± 0.10	0.62 ± 0.12
A	Combined	**94.03 ± 8.56**	0.94 ± 0.08	**0.94 ± 0.11**	0.93 ± 0.13	0.94 ± 0.09	0.97 ± 0.06
B	3DFD	96.50 ± 6.26	0.98 ± 0.04	0.97 ± 0.11	0.97 ± 0.11	0.96 ± 0.06	1.00 ± 0.01
B	Fixation Duration	83.75 ± 5.43	0.74 ± 0.15	0.90 ± 0.11	0.88 ± 0.17	0.81 ± 0.05	0.91 ± 0.06
B	Combined	**98.50 ± 4.74**	1.00 ± 0.00	**0.97 ± 0.08**	0.97 ± 0.09	0.98 ± 0.05	1.00 ± 0.00
C	3DFD	95.33 ± 8.34	0.99 ± 0.04	0.93 ± 0.14	0.93 ± 0.14	0.95 ± 0.08	0.99 ± 0.02
C	Fixation Duration	87.33 ± 9.14	0.83 ± 0.16	0.94 ± 0.09	0.89 ± 0.15	0.86 ± 0.09	0.91 ± 0.11
C	Combined	**97.33 ± 5.62**	1.00 ± 0.00	**0.96 ± 0.09**	0.95 ± 0.11	0.97 ± 0.06	1.00 ± 0.01
D	3DFD	98.00 ± 6.32	0.97 ± 0.08	1.00 ± 0.00	1.00 ± 0.00	0.98 ± 0.08	1.00 ± 0.00
D	Fixation Duration	94.00 ± 13.50	0.95 ± 0.16	0.97 ± 0.11	0.97 ± 0.11	0.94 ± 0.14	1.00 ± 0.00
D	Combined	**100.00 ± 0.00**	1.00 ± 0.00	**1.00 ± 0.00**	1.00 ± 0.00	1.00 ± 0.00	1.00 ± 0.00
E	3DFD	**100.00 ± 0.00**	1.00 ± 0.00	**1.00 ± 0.00**	1.00 ± 0.00	1.00 ± 0.00	1.00 ± 0.00
E	Fixation Duration	94.00 ± 13.50	0.93 ± 0.17	0.97 ± 0.11	0.95 ± 0.16	0.93 ± 0.14	0.98 ± 0.05
E	Combined	**100.00 ± 0.00**	1.00 ± 0.00	**1.00 ± 0.00**	1.00 ± 0.00	1.00 ± 0.00	1.00 ± 0.00
StimP	3DFD	75.14 ± 7.28	0.57 ± 0.09	0.89 ± 0.10	0.79 ± 0.21	0.71 ± 0.06	0.77 ± 0.08
StimP	Fixation Duration	59.84 ± 9.45	0.33 ± 0.06	0.81 ± 0.08	0.55 ± 0.23	0.54 ± 0.06	0.61 ± 0.06
StimP	Combined	**80.86 ± 7.31**	0.66 ± 0.05	**0.92 ± 0.10**	0.85 ± 0.17	0.78 ± 0.06	0.83 ± 0.06
StimN	3DFD	73.38 ± 9.99	0.60 ± 0.11	0.83 ± 0.13	0.74 ± 0.22	0.70 ± 0.10	0.80 ± 0.09
StimN	Fixation Duration	57.75 ± 9.94	0.39 ± 0.10	0.73 ± 0.05	0.52 ± 0.16	0.54 ± 0.08	0.60 ± 0.08
StimN	Combined	**77.67 ± 9.37**	0.64 ± 0.09	**0.88 ± 0.13**	0.80 ± 0.21	0.75 ± 0.09	0.82 ± 0.07

Note. Within each stimulus group, boldface indicates the feature set with the highest values for both Accuracy and Specificity across the three feature sets. In cases of tied highest values, boldface was assigned to the feature set that also showed the highest value for the other metric.

**Table 7:** Classification performance for N vs. ER Random Forest classifier using $M_{P}^{s}$ = 10 longest fixation duration locations (10-fold cross-validation).

Stimulus Set	Feature Set	Accuracy	Sensitivity	Specificity	Precision	F1	AUC
A	3DFD	92.15 ± 9.48	0.80 ± 0.29	0.95 ± 0.11	0.84 ± 0.35	0.88 ± 0.18	0.94 ± 0.08
A	FixDur	79.85 ± 12.08	0.52 ± 0.30	0.92 ± 0.15	0.74 ± 0.37	0.70 ± 0.16	0.84 ± 0.09
A	Combined	**93.70 ± 7.00**	0.84 ± 0.30	**0.96 ± 0.09**	0.86 ± 0.33	0.89 ± 0.17	0.98 ± 0.03
B	3DFD	95.12 ± 8.22	0.86 ± 0.31	0.96 ± 0.09	0.87 ± 0.32	0.91 ± 0.18	1.00 ± 0.00
B	FixDur	83.87 ± 12.96	0.66 ± 0.37	0.90 ± 0.16	0.78 ± 0.34	0.75 ± 0.18	0.91 ± 0.13
B	Combined	**95.62 ± 9.34**	0.90 ± 0.32	**0.95 ± 0.11**	0.86 ± 0.33	0.92 ± 0.19	1.00 ± 0.00
C	3DFD	92.83 ± 9.69	0.87 ± 0.31	0.94 ± 0.10	0.85 ± 0.32	0.89 ± 0.18	0.99 ± 0.02
C	FixDur	86.50 ± 9.98	0.67 ± 0.38	0.94 ± 0.14	0.72 ± 0.42	0.78 ± 0.19	0.90 ± 0.17
C	Combined	**93.67 ± 8.56**	0.84 ± 0.32	**0.95 ± 0.10**	0.86 ± 0.33	0.90 ± 0.18	1.00 ± 0.00
D	3DFD	**97.50 ± 7.91**	0.90 ± 0.32	**0.97 ± 0.08**	0.90 ± 0.32	0.94 ± 0.18	1.00 ± 0.00
D	FixDur	95.50 ± 9.56	0.85 ± 0.34	0.97 ± 0.08	0.90 ± 0.32	0.92 ± 0.19	1.00 ± 0.00
D	Combined	95.50 ± 9.56	0.80 ± 0.42	0.97 ± 0.08	0.80 ± 0.42	0.89 ± 0.24	1.00 ± 0.00
E	3DFD	98.00 ± 6.32	0.85 ± 0.34	1.00 ± 0.00	0.90 ± 0.32	0.93 ± 0.17	1.00 ± 0.00
E	FixDur	97.50 ± 7.91	0.90 ± 0.32	0.97 ± 0.08	0.90 ± 0.32	0.94 ± 0.18	1.00 ± 0.00
E	Combined	**100.00 ± 0.00**	0.90 ± 0.32	**1.00 ± 0.00**	0.90 ± 0.32	0.95 ± 0.16	1.00 ± 0.00
StimP	3DFD	80.08 ± 9.90	0.54 ± 0.22	0.93 ± 0.08	0.77 ± 0.32	0.72 ± 0.11	0.81 ± 0.09
StimP	FixDur	74.32 ± 13.95	0.38 ± 0.25	0.94 ± 0.06	0.68 ± 0.31	0.63 ± 0.12	0.73 ± 0.09
StimP	Combined	**87.05 ± 6.96**	0.67 ± 0.26	**0.96 ± 0.08**	0.85 ± 0.32	0.81 ± 0.14	0.89 ± 0.07
StimN	3DFD	78.33 ± 9.97	0.53 ± 0.20	0.91 ± 0.10	0.74 ± 0.32	0.71 ± 0.12	0.79 ± 0.10
StimN	FixDur	64.58 ± 16.28	0.20 ± 0.16	0.90 ± 0.08	0.51 ± 0.36	0.50 ± 0.11	0.60 ± 0.10
StimN	Combined	**81.97 ± 7.23**	0.57 ± 0.21	**0.93 ± 0.08**	0.78 ± 0.32	0.75 ± 0.11	0.84 ± 0.07

Note. Within each stimulus group, boldface indicates the feature set with the highest values for both Accuracy and Specificity across the three feature sets. In cases of tied highest values, boldface was assigned to the feature set that also showed the highest value for the other metric.

**Table 8:** Classification performance of Random Forest classifier for EC vs. ER using $M_{P}^{s}$ = 10 longest fixation duration locations (10-fold cross-validation).

Stimulus Set	Feature Set	Accuracy	Sensitivity	Specificity	Precision	F1	AUC
A	3DFD	94.00 ± 8.68	0.68 ± 0.47	0.85 ± 0.32	0.66 ± 0.47	0.76 ± 0.25	0.98 ± 0.02
A	FixDur	78.92 ± 10.93	0.47 ± 0.36	0.81 ± 0.30	0.63 ± 0.45	0.63 ± 0.19	0.85 ± 0.07
A	Combined	**94.92 ± 8.14**	0.68 ± 0.47	**0.86 ± 0.31**	0.67 ± 0.47	0.77 ± 0.26	1.00 ± 0.01
B	3DFD	96.25 ± 6.72	0.68 ± 0.47	0.88 ± 0.32	0.70 ± 0.48	0.78 ± 0.26	1.00 ± 0.00
B	FixDur	90.62 ± 9.43	0.56 ± 0.42	0.88 ± 0.32	0.70 ± 0.48	0.72 ± 0.23	0.95 ± 0.06
B	Combined	**96.88 ± 7.93**	0.69 ± 0.48	**0.88 ± 0.32**	0.70 ± 0.48	0.79 ± 0.26	1.00 ± 0.00
C	3DFD	95.00 ± 8.05	0.66 ± 0.46	0.88 ± 0.32	0.70 ± 0.48	0.77 ± 0.26	1.00 ± 0.00
C	FixDur	93.06 ± 10.33	0.62 ± 0.45	0.88 ± 0.32	0.70 ± 0.48	0.74 ± 0.24	0.98 ± 0.05
C	Combined	**96.67 ± 8.05**	0.69 ± 0.48	**0.88 ± 0.32**	0.70 ± 0.48	0.78 ± 0.26	1.00 ± 0.00
D	3DFD	**97.50 ± 7.91**	0.70 ± 0.48	**0.88 ± 0.32**	0.70 ± 0.48	0.79 ± 0.27	1.00 ± 0.00
D	FixDur	92.50 ± 12.08	0.63 ± 0.46	0.88 ± 0.32	0.70 ± 0.48	0.74 ± 0.25	1.00 ± 0.00
D	Combined	95.00 ± 10.54	0.67 ± 0.47	0.88 ± 0.32	0.70 ± 0.48	0.77 ± 0.26	1.00 ± 0.00
E	3DFD	**100.00 ± 0.00**	0.70 ± 0.48	**0.90 ± 0.32**	0.70 ± 0.48	0.80 ± 0.26	1.00 ± 0.00
E	FixDur	97.50 ± 7.91	0.67 ± 0.47	0.90 ± 0.32	0.70 ± 0.48	0.77 ± 0.25	1.00 ± 0.00
E	Combined	**100.00 ± 0.00**	0.70 ± 0.48	**0.90 ± 0.32**	0.70 ± 0.48	0.80 ± 0.26	1.00 ± 0.00
StimP	3DFD	79.21 ± 9.41	0.51 ± 0.36	0.80 ± 0.30	0.62 ± 0.45	0.65 ± 0.19	0.90 ± 0.04
StimP	FixDur	70.31 ± 12.98	0.33 ± 0.25	0.80 ± 0.30	0.58 ± 0.43	0.55 ± 0.13	0.65 ± 0.10
StimP	Combined	**87.41 ± 7.27**	0.59 ± 0.41	**0.83 ± 0.30**	0.64 ± 0.46	0.71 ± 0.22	0.96 ± 0.03
StimN	3DFD	76.79 ± 9.55	0.50 ± 0.36	0.77 ± 0.30	0.62 ± 0.45	0.64 ± 0.21	0.92 ± 0.05
StimN	FixDur	64.62 ± 16.29	0.29 ± 0.21	0.76 ± 0.28	0.54 ± 0.42	0.51 ± 0.14	0.65 ± 0.07
StimN	Combined	**82.42 ± 8.13**	0.55 ± 0.39	**0.80 ± 0.30**	0.64 ± 0.46	0.67 ± 0.21	0.95 ± 0.06

Note. Within each stimulus group, boldface indicates the feature set with the highest values for both Accuracy and Specificity across the three feature sets. In cases of tied highest values, boldface was assigned to the feature set that also showed the highest value for the other metric.

## Data Availability

Data, along with the data processing pipeline codes, are contained within the Supplementary Materials. The original contributions presented in this study are included in the article/Appendix material. Further inquiries can be directed to the corresponding authors.

## Supplementary Materials

The following supporting information can be downloaded at: https://www.mdpi.com/article/10.3390/jemr19030062/s1, Supplementary File contains MATLAB and Python code, data files, and stimuli images used in this study. It includes a Word document describing folder contents and usage instructions.

## Abbreviations

The following abbreviations are used in this manuscript:

FD	Fractal dimension
3DFD	Three-dimensional fractal dimension
MRI	Magnetic resonance imaging
N	Naïve
ER	Expert registrar
EC	Expert consultant
NN	Naïve normal
NP	Naïve pathology
ECN	Expert consultant normal
ECP	Expert consultant pathology
ERN	Expert registrar normal
ERP	Expert registrar pathology
AUC	Area under curve

## Appendix A

### Appendix A.1. Eye-Tracking Report Template

**Table A1:** A template of the report containing the fixation duration values at different fixation locations for participant ID N02. The total number of fixation points in this template report is $N_{P}^{s}$ = $N_{2}^{1}$ by considering the subject number P = 2 and the stimulus number for this participant being s= 1.

Participant ID	Stimulus Name	X Coordinate of Fixation	Y Coordinate of Fixation	Fixation Duration at the (X, Y) Coordinates
N02	BRAIN_1_PATH_Meningioma_ax.png	$x\left(1\right)$	$y\left(1\right)$	$FixDur\left(1\right)$
N02	BRAIN_1_PATH_Meningioma_ax.png	$x\left(2\right)$	$y\left(2\right)$	$FixDur\left(2\right)$
⋮	⋮	⋮	⋮	⋮
N02	BRAIN_1_PATH_Meningioma_ax.png	$x\left(N_{2}^{1}\right)$	$y\left(N_{2}^{1}\right)$	$FixDur\left(N_{2}^{1}\right)$

### Appendix A.3. Eye-Tracking Image Data Processing

#### Masks for Removing Background Pixels in Stimuli Images

A method was implemented in Matlab to create masks by manually clicking on the stimulus image. Using this method, masks were manually created from each of the 39 stimulus images and applied to the heat maps of fixation duration and the 3DFD maps. The masks ensured that the background pixels in the stimulus image were removed from analysis.

### Appendix A.4. Software Programming Languages Used for Data Processing

#### Appendix A.4.1. Linear Mixed-Effects Modelling and Statistical Analysis

All the data processing for statistical analysis, such as data preparation, processing, mixed-effects modeling, and effect size computation, was performed in MATLAB R2025b using the Statistics and Machine Learning Toolbox. The numerical values in the statistical analysis results tables were generated programmatically to ensure reproducibility across all the stimulus groups.

#### Appendix A.4.2. Machine Learning Models

All the data preparation for feeding into the three machine learning models and the development of the three models were performed in MATLAB R2025b. The numerical values in the tables summarizing the machine learning results were generated programmatically to ensure reproducibility across all the stimulus groups and the three binary classifiers.

## Appendix B

### Appendix B.2. Linear Mixed-Effects Modelling and Statistical Analysis

#### Appendix B.2.1. H1: Expertise-Related Differences in Fixation-Duration Behavior

**Table A2:** Fixed-effects results for Fixation Duration × ImageType across stimulus groups (A–E, and StimP). For these results, we chose the $M_{P}^{s}$ = 5, which corresponds to the first 5 maximum fixation durations for each stimulus for each participant.

Stimulus Group	Predictor	β	SE	t	df	*p*	*p_FDR*	Cohen’s d
A	ImageType (tumors)	−8.058	8.898	−0.9	10,044	0.37	0.365	−0.038
A	EC × ImageType (tumors)	29.721	10.49	2.89	10,044	**0.01**	**0.01 ***	0.139
A	ER × ImageType (tumors)	73.456	11.56	6.36	10,044	**0.000**	**0.000 *****	0.343
B	ImageType (cerebrovascular pathologies)	−7.965	12.039	−0.66	8034	0.508	0.508	−0.039
B	EC × ImageType (cerebrovascular pathologies)	52.077	14.047	3.71	8034	**0.000**	**0.000 *****	0.257
B	ER × ImageType (cerebrovascular pathologies)	76.530	15.472	4.95	8034	**0.000**	**0.000 *****	0.377
C	ImageType (others)	1.438	14.337	0.10	7699	0.920	0.920	0.007
C	EC × ImageType (others)	37.062	15.924	2.33	7699	**0.020**	**0.040 ***	0.182
C	ER × ImageType (others)	46.653	17.540	2.66	7699	**0.008**	**0.024 ***	0.229
D	ImageType (inflammatory)	25.427	21.410	1.19	7029	0.235	0.437	0.119
D	EC × ImageType (inflammatory)	17.792	27.723	0.64	7029	0.521	0.521	0.083
D	ER × ImageType (inflammatory)	26.914	30.536	0.88	7029	0.378	0.454	0.126
E	ImageType (malfomations)	−20.539	20.958	−0.98	7029	0.327	0.327	−0.101
E	EC × ImageType (malfomations)	39.430	26.291	1.50	7029	0.134	0.201	0.194
E	ER × ImageType (malfomations)	82.705	28.959	2.86	7029	**0.004**	**0.012 ***	0.408
StimP	ImageType (all pathologies)	−5.436	7.913	−0.7	13,059	0.492	0.492	−0.025
StimP	EC × ImageType (all pathologies)	35.472	8.872	4.00	13,059	**0.000**	**0.000 *****	0.169
StimP	ER × ImageType (all pathologies)	67.911	9.772	6.95	13,059	**0.000**	**0.000 *****	0.318

Note. Fixed-effects estimates are from linear mixed-effects models of the formula *Fixation Duration ~ Group × ImageType* + (1*|Subject*) + (1*|StimuliID*). The naïve group (N) served as the reference category, and normal images served as the reference level for ImageType. *β* = fixation duration estimate; *d* = Cohen’s d. Significant *p* and *p_FDR* values (*p/p_FDR* < 0.05) are shown in bold. Significance levels on FDR-corrected *p*-values (*p_FDR*) are marked as: *p_FDR* < 0.05 (*), *p_FDR* < 0.001 (***).

#### Appendix B.2.2. H2: Expertise-Related Differences in Structural Complexity (3DFD) at Fixation Locations For $M_{P}^{s}$ = 5 Maximum Fxation Durations

**Table A3:** Fixed-Effects Results for 3DFD × ImageType across stimulus groups (A–E, and StimP). For these results, we chose the $M_{P}^{s}$ = 5, which corresponds to the first 5 maximum fixation durations for each stimulus for each participant.

Stimulus Group	Predictor	β	SE	t	df	*p*	*p_FDR*	Cohen’s d
A	ImageType (tumors)	0.031	0.010	2.96	10,044	**0.009**	**0.009 ****	0.386
A	EC × ImageType (tumors)	−0.002	0.004	−0.63	10,044	0.53	0.532	0.025
A	ER × ImageType (tumors)	−0.003	0.004	−0.78	10,044	0.43	0.52	−0.037
B	ImageType (cerebrovascular pathologies)	0.025	0.015	1.68	8034	0.092	0.110	0.355
B	EC × ImageType (cerebrovascular pathologies)	−0.012	0.005	−2.42	8034	**0.016**	**0.026 ***	−0.171
B	ER × ImageType (cerebrovascular pathologies)	−0.018	0.005	−3.28	8034	**0.001**	**0.003 ****	−0.256
C	ImageType (others)	0.004	0.016	0.24	7699	0.808	0.808	0.058
C	EC × ImageType (others)	−0.005	0.005	−0.84	7699	0.398	0.478	−0.072
C	ER × ImageType (others)	−0.006	0.006	−0.96	7699	0.338	0.478	−0.087
D	ImageType (inflammatory)	−0.003	0.028	−0.11	7029	0.909	0.909	−0.034
D	EC × ImageType (inflammatory)	0.041	0.011	3.61	7029	**0.000**	**0.000 *****	0.467
D	ER × ImageType (inflammatory)	0.054	0.013	4.29	7029	**0.000**	**0.000 *****	0.615
E	ImageType (malfomations)	0.089	0.028	3.24	7029	**0.001**	**0.003 ****	1.239
E	EC × ImageType (malfomations)	0.005	0.009	0.52	7029	0.606	0.649	0.070
E	ER × ImageType (malfomations)	0.005	0.010	0.45	7029	0.649	0.649	0.070
StimP	ImageType (all pathologies)	0.027	0.009	2.93	13,059	**0.003**	**0.009 ****	0.327
StimP	EC × ImageType (all pathologies)	−0.002	0.003	−0.62	13,059	0.533	0.533	−0.024
StimP	ER × ImageType (all pathologies)	−0.003	0.004	−0.9	13,059	0.367	0.44	−0.036

Note. Fixed-effects estimates are from linear mixed-effects models of the formula *Mean3DFD ~ Group × ImageType* + (1*|Subject*) + (1*|StimuliID*). The naïve group (N) served as the reference category, and normal images served as the reference level for ImageType. *β* = unstandardized 3DFD estimate; *d* = Cohen’s d. Significant *p* and *p_FDR* values (*p/p_FDR* < 0.05) are shown in bold. Significance levels on FDR-corrected *p*-values (*p_FDR*) are marked as: *p_FDR* < 0.05 (*), *p_FDR* < 0.01 (**), *p_FDR* < 0.001 (***).

### Appendix B.3. Non-Linear Modelling Using Supervised Machine Learning Methods

#### Appendix B.3.1. H3: Unbalanced Participant Groups of N (*N_p_* = 29), EC (*N_p_* = 24), ER (*N_p_* = 16)

Classifiers trained on the combined feature representation generally achieve higher accuracy and specificity across stimulus sets. Performance differences between Random Forest and SVM classifiers are consistent with the N vs. EC and N vs. ER classifiers for the combined feature space. The performance differences between Random Forest and SVM classifiers are consistent with the interaction-driven structure of the combined feature space, with tree-based models more effectively capturing non-linear interactions between geometric complexity and fixation behavior.

Classifiers trained on the combined feature representation generally achieve higher accuracy and specificity across stimulus sets. Performance differences between Random Forest and SVM classifiers are consistent with the N vs. EC and N vs. ER classifiers for the combined feature space. The performance differences between Random Forest and SVM classifiers are consistent with the interaction-driven structure of the combined feature space, with tree-based models more effectively capturing non-linear interactions between geometric complexity and fixation behavior.

Classifiers trained on the combined feature representation generally achieve higher accuracy and specificity across stimulus sets. Performance differences between Random Forest and SVM classifiers are consistent with the N vs. EC and N vs. ER classifiers for the combined feature space. The performance differences between Random Forest and SVM classifiers are consistent with the interaction-driven structure of the combined feature space, with tree-based models more effectively capturing non-linear interactions between geometric complexity and fixation behavior.

**Table A4:** Classification performance of SVM classifier for N vs. EC using $M_{P}^{s}$ = 10 longest fixation duration locations (10-fold cross-validation).

Stimulus Group	Feature Set	Accuracy	Sensitivity	Specificity	Precision	F1	AUC
A	3DFD	**75.53 ± 13.64**	0.97 ± 0.06	0.61 ± 0.26	0.67 ± 0.18	0.73 ± 0.14	0.86 ± 0.10
A	Fixation Duration	50.67 ± 14.82	0.21 ± 0.16	0.81 ± 0.17	0.47 ± 0.23	0.43 ± 0.12	0.59 ± 0.13
A	Combined	56.67 ± 18.66	0.00 ± 0.00	**1.00 ± 0.00**	0.00 ± 0.00	0.35 ± 0.08	0.82 ± 0.13
B	3DFD	**84.58 ± 13.71**	1.00 ± 0.00	0.69 ± 0.24	0.76 ± 0.20	0.82 ± 0.14	0.97 ± 0.04
B	Fixation Duration	70.83 ± 20.26	0.85 ± 0.28	0.66 ± 0.23	0.68 ± 0.18	0.69 ± 0.20	0.84 ± 0.14
B	Combined	54.67 ± 16.87	0.10 ± 0.32	**0.95 ± 0.16**	0.03 ± 0.11	0.37 ± 0.11	0.96 ± 0.06
C	3DFD	**78.78 ± 17.26**	0.99 ± 0.03	0.63 ± 0.31	0.72 ± 0.24	0.77 ± 0.18	0.94 ± 0.08
C	Fixation Duration	58.78 ± 25.47	0.67 ± 0.46	0.68 ± 0.29	0.40 ± 0.32	0.55 ± 0.28	0.77 ± 0.20
C	Combined	54.00 ± 16.76	0.10 ± 0.32	**0.94 ± 0.18**	0.03 ± 0.09	0.36 ± 0.10	0.88 ± 0.15
D	3DFD	**86.00 ± 18.97**	1.00 ± 0.00	**0.76 ± 0.33**	0.80 ± 0.23	0.84 ± 0.22	0.98 ± 0.05
D	Fixation Duration	82.33 ± 14.74	1.00 ± 0.00	0.67 ± 0.32	0.73 ± 0.20	0.79 ± 0.20	0.97 ± 0.11
D	Combined	70.33 ± 23.75	0.90 ± 0.32	0.61 ± 0.34	0.60 ± 0.30	0.67 ± 0.26	0.97 ± 0.11
E	3DFD	**88.00 ± 13.98**	0.93 ± 0.17	**0.85 ± 0.20**	0.86 ± 0.19	0.87 ± 0.15	0.97 ± 0.07
E	Fixation Duration	70.33 ± 23.75	0.90 ± 0.32	0.62 ± 0.29	0.60 ± 0.30	0.69 ± 0.25	0.93 ± 0.12
E	Combined	60.00 ± 24.94	0.60 ± 0.52	0.75 ± 0.33	0.38 ± 0.39	0.54 ± 0.28	1.00 ± 0.00
StimP	3DFD	58.21 ± 18.81	0.55 ± 0.30	0.71 ± 0.19	0.58 ± 0.14	0.56 ± 0.18	0.69 ± 0.10
StimP	Fixation Duration	53.04 ± 13.04	0.26 ± 0.15	0.80 ± 0.11	0.49 ± 0.17	0.47 ± 0.10	0.58 ± 0.07
StimP	Combined	**56.67 ± 18.66**	0.00 ± 0.00	**1.00 ± 0.00**	0.00 ± 0.00	0.35 ± 0.08	0.66 ± 0.10
StimN	3DFD	**58.40 ± 20.01**	0.48 ± 0.31	0.78 ± 0.21	0.65 ± 0.22	0.55 ± 0.20	0.71 ± 0.12
StimN	Fixation Duration	56.67 ± 15.93	0.23 ± 0.15	0.85 ± 0.06	0.52 ± 0.17	0.48 ± 0.13	0.54 ± 0.10
StimN	Combined	56.67 ± 18.66	0.00 ± 0.00	**1.00 ± 0.00**	0.00 ± 0.00	0.35 ± 0.08	0.68 ± 0.10

Note. Within each stimulus group, boldface indicates the feature set with the highest values for both Accuracy and Specificity across the three feature sets. In cases of tied highest values, boldface was assigned to the feature set that also showed the highest value for the other metric.

**Table A5:** Classification performance of SVM classifier for N vs. ER using $M_{P}^{s}$ = 10 longest fixation-duration locations (10-fold cross-validation).

Stimulus Set	Feature Set	Accuracy	Sensitivity	Specificity	Precision	F1	AUC
A	3DFD	64.60 ± 22.30	0.06 ± 0.11	0.98 ± 0.04	0.24 ± 0.42	0.43 ± 0.15	0.87 ± 0.10
A	FixDur	**66.70 ± 20.28**	0.08 ± 0.08	0.98 ± 0.04	0.53 ± 0.50	0.45 ± 0.10	0.80 ± 0.12
A	Combined	65.00 ± 23.21	0.00 ± 0.00	**1.00 ± 0.00**	0.00 ± 0.00	0.38 ± 0.09	0.85 ± 0.11
B	3DFD	**67.62 ± 25.08**	0.53 ± 0.45	0.86 ± 0.21	0.53 ± 0.43	0.60 ± 0.27	0.96 ± 0.03
B	FixDur	66.12 ± 23.04	0.03 ± 0.08	**1.00 ± 0.00**	0.20 ± 0.42	0.41 ± 0.12	0.91 ± 0.11
B	Combined	65.00 ± 23.21	0.00 ± 0.00	**1.00 ± 0.00**	0.00 ± 0.00	0.38 ± 0.09	0.92 ± 0.11
C	3DFD	60.83 ± 19.71	0.00 ± 0.00	0.95 ± 0.10	0.00 ± 0.00	0.37 ± 0.08	0.90 ± 0.14
C	FixDur	**65.00 ± 23.21**	0.00 ± 0.00	**1.00 ± 0.00**	0.00 ± 0.00	0.38 ± 0.09	0.81 ± 0.17
C	Combined	**65.00 ± 23.21**	0.00 ± 0.00	**1.00 ± 0.00**	0.00 ± 0.00	0.38 ± 0.09	0.89 ± 0.11
D	3DFD	**78.00 ± 22.39**	0.60 ± 0.47	0.92 ± 0.14	0.65 ± 0.47	0.71 ± 0.29	0.94 ± 0.17
D	FixDur	60.00 ± 20.00	0.30 ± 0.48	0.85 ± 0.22	0.13 ± 0.22	0.44 ± 0.18	1.00 ± 0.00
D	Combined	65.00 ± 23.21	0.00 ± 0.00	**1.00 ± 0.00**	0.00 ± 0.00	0.38 ± 0.09	1.00 ± 0.00
E	3DFD	**81.50 ± 27.99**	0.70 ± 0.48	0.94 ± 0.12	0.65 ± 0.47	0.76 ± 0.33	1.00 ± 0.00
E	FixDur	65.00 ± 23.21	0.00 ± 0.00	**1.00 ± 0.00**	0.00 ± 0.00	0.38 ± 0.09	0.94 ± 0.13
E	Combined	65.00 ± 23.21	0.00 ± 0.00	**1.00 ± 0.00**	0.00 ± 0.00	0.38 ± 0.09	0.94 ± 0.12
StimP	3DFD	64.50 ± 22.88	0.01 ± 0.03	0.99 ± 0.02	0.10 ± 0.32	0.39 ± 0.10	0.71 ± 0.19
StimP	FixDur	**65.82 ± 20.09**	0.10 ± 0.05	0.97 ± 0.03	0.62 ± 0.33	0.46 ± 0.10	0.67 ± 0.09
StimP	Combined	65.00 ± 23.21	0.00 ± 0.00	**1.00 ± 0.00**	0.00 ± 0.00	0.38 ± 0.09	0.75 ± 0.16
StimN	3DFD	64.75 ± 23.10	0.01 ± 0.02	1.00 ± 0.01	0.10 ± 0.32	0.39 ± 0.10	0.75 ± 0.14
StimN	FixDur	62.42 ± 20.96	0.02 ± 0.02	0.96 ± 0.03	0.25 ± 0.34	0.39 ± 0.09	0.55 ± 0.09
StimN	Combined	**65.00 ± 23.21**	0.00 ± 0.00	**1.00 ± 0.00**	0.00 ± 0.00	0.38 ± 0.09	0.72 ± 0.16

Note. Within each stimulus group, boldface indicates the feature set with the highest values for both Accuracy and Specificity across the three feature sets. In cases of tied highest values, boldface was assigned to the feature set that also showed the highest value for the other metric.

**Table A6:** Classification performance of SVM classifier for EC vs. ER using $M_{P}^{s}$ = 10 longest fixation-duration locations (10-fold cross-validation).

Stimulus Set	Feature Set	Accuracy	Sensitivity	Specificity	Precision	F1	AUC
A	3DFD	**64.67 ± 27.63**	0.57 ± 0.48	0.61 ± 0.32	0.58 ± 0.44	0.59 ± 0.31	0.96 ± 0.06
A	FixDur	51.50 ± 22.35	0.34 ± 0.37	0.61 ± 0.30	0.52 ± 0.42	0.45 ± 0.21	0.82 ± 0.11
A	Combined	43.92 ± 31.07	0.00 ± 0.00	**0.75 ± 0.42**	0.00 ± 0.00	0.28 ± 0.16	0.93 ± 0.09
B	3DFD	**82.71 ± 20.30**	0.70 ± 0.48	**0.70 ± 0.31**	0.62 ± 0.45	0.71 ± 0.29	0.97 ± 0.03
B	FixDur	47.29 ± 23.22	0.20 ± 0.35	0.69 ± 0.34	0.33 ± 0.45	0.38 ± 0.22	0.91 ± 0.12
B	Combined	38.12 ± 22.35	0.00 ± 0.00	0.69 ± 0.41	0.00 ± 0.00	0.26 ± 0.12	0.94 ± 0.07
C	3DFD	**70.83 ± 28.93**	0.61 ± 0.51	0.65 ± 0.31	0.59 ± 0.44	0.64 ± 0.33	0.98 ± 0.04
C	FixDur	50.28 ± 30.83	0.37 ± 0.48	0.62 ± 0.34	0.30 ± 0.41	0.45 ± 0.32	0.98 ± 0.02
C	Combined	39.17 ± 22.24	0.00 ± 0.00	**0.70 ± 0.40**	0.00 ± 0.00	0.26 ± 0.12	0.99 ± 0.02
D	3DFD	**85.00 ± 17.48**	0.67 ± 0.47	**0.74 ± 0.33**	0.62 ± 0.46	0.70 ± 0.25	1.00 ± 0.00
D	FixDur	54.17 ± 30.24	0.35 ± 0.47	0.68 ± 0.33	0.32 ± 0.43	0.47 ± 0.32	0.88 ± 0.21
D	Combined	40.00 ± 22.50	0.10 ± 0.32	0.68 ± 0.38	0.05 ± 0.16	0.30 ± 0.19	1.00 ± 0.00
E	3DFD	**91.67 ± 13.61**	0.70 ± 0.48	**0.81 ± 0.32**	0.65 ± 0.47	0.76 ± 0.27	0.94 ± 0.14
E	FixDur	56.67 ± 30.88	0.40 ± 0.52	0.66 ± 0.31	0.28 ± 0.39	0.48 ± 0.31	0.83 ± 0.26
E	Combined	42.50 ± 22.03	0.10 ± 0.32	0.70 ± 0.36	0.05 ± 0.16	0.31 ± 0.19	0.94 ± 0.14
StimP	3DFD	47.24 ± 27.86	0.32 ± 0.42	0.62 ± 0.33	0.38 ± 0.42	0.43 ± 0.29	0.88 ± 0.11
StimP	FixDur	49.96 ± 21.58	0.14 ± 0.13	0.72 ± 0.32	0.54 ± 0.46	0.40 ± 0.17	0.66 ± 0.09
StimP	Combined	**59.17 ± 35.45**	0.00 ± 0.00	**0.90 ± 0.32**	0.00 ± 0.00	0.34 ± 0.16	0.86 ± 0.12
StimN	3DFD	44.67 ± 24.84	0.26 ± 0.37	0.63 ± 0.34	0.37 ± 0.41	0.39 ± 0.26	0.87 ± 0.11
StimN	FixDur	47.37 ± 22.35	0.05 ± 0.05	0.74 ± 0.31	0.47 ± 0.43	0.34 ± 0.14	0.64 ± 0.12
StimN	Combined	**59.17 ± 35.45**	0.00 ± 0.00	**0.90 ± 0.32**	0.00 ± 0.00	0.34 ± 0.16	0.84 ± 0.11

Note. Within each stimulus group, boldface indicates the feature set with the highest values for both Accuracy and Specificity across the three feature sets. In cases of tied highest values, boldface was assigned to the feature set that also showed the highest value for the other metric.

#### Appendix B.3.2. H3: Balanced Participant Groups of N (*N_p_* = 16), EC (*N_p_* = 16), ER (*N_p_* = 16)

In agreement with the results in Figure A3 for the unbalanced participant groups, for the balanced group (*N_p_* = 16), the classifiers trained on the combined feature representation achieved either higher or very close accuracy and other performance metrics across the stimulus sets for all the Random Forest binary classifiers.

**Table A7:** Classification performance of RBF classifier for N vs. EC using $M_{P}^{s}$ = 10 longest fixation-duration locations (10-fold cross-validation), with balanced data for each class (n = 16).

Stimulus Set	Feature Set	Accuracy	Sensitivity	Specificity	Precision	F1	AUC
A	3DFD	**86.17 ± 18.27**	0.85 ± 0.30	**0.70 ± 0.41**	0.75 ± 0.36	0.75 ± 0.24	0.96 ± 0.05
A	FixDur	59.67 ± 11.28	0.59 ± 0.26	0.52 ± 0.31	0.60 ± 0.33	0.53 ± 0.13	0.70 ± 0.14
A	Combined	86.00 ± 20.57	0.87 ± 0.31	0.67 ± 0.45	0.74 ± 0.37	0.73 ± 0.27	0.96 ± 0.05
B	3DFD	95.83 ± 10.58	0.89 ± 0.31	0.85 ± 0.34	0.85 ± 0.34	0.86 ± 0.22	0.99 ± 0.04
B	FixDur	84.58 ± 18.40	0.76 ± 0.30	0.74 ± 0.42	0.81 ± 0.34	0.75 ± 0.26	0.94 ± 0.13
B	Combined	**96.67 ± 10.54**	0.90 ± 0.32	**0.85 ± 0.34**	0.85 ± 0.34	0.87 ± 0.22	1.00 ± 0.01
C	3DFD	95.56 ± 9.37	0.88 ± 0.31	0.83 ± 0.32	0.84 ± 0.32	0.85 ± 0.21	0.99 ± 0.04
C	FixDur	85.56 ± 10.94	0.71 ± 0.31	0.81 ± 0.33	0.78 ± 0.34	0.75 ± 0.19	0.93 ± 0.13
C	Combined	**98.89 ± 3.51**	0.90 ± 0.32	**0.88 ± 0.31**	0.88 ± 0.32	0.89 ± 0.21	1.00 ± 0.00
D	3DFD	**100.00 ± 0.00**	0.90 ± 0.32	**0.90 ± 0.32**	0.90 ± 0.32	0.90 ± 0.21	1.00 ± 0.00
D	FixDur	96.67 ± 10.54	0.85 ± 0.34	0.90 ± 0.32	0.90 ± 0.32	0.87 ± 0.22	1.00 ± 0.00
D	Combined	96.67 ± 10.54	0.85 ± 0.34	0.90 ± 0.32	0.90 ± 0.32	0.87 ± 0.22	1.00 ± 0.00
E	3DFD	**100.00 ± 0.00**	0.90 ± 0.32	**0.90 ± 0.32**	0.90 ± 0.32	0.90 ± 0.21	1.00 ± 0.00
E	FixDur	96.67 ± 10.54	0.85 ± 0.34	0.90 ± 0.32	0.90 ± 0.32	0.87 ± 0.22	1.00 ± 0.00
E	Combined	**100.00 ± 0.00**	0.90 ± 0.32	**0.90 ± 0.32**	0.90 ± 0.32	0.90 ± 0.21	1.00 ± 0.00
StimP	3DFD	69.65 ± 15.13	0.77 ± 0.28	0.51 ± 0.35	0.63 ± 0.33	0.60 ± 0.18	0.81 ± 0.13
StimP	FixDur	58.60 ± 5.54	0.55 ± 0.22	0.53 ± 0.25	0.59 ± 0.29	0.53 ± 0.11	0.67 ± 0.10
StimP	Combined	**72.94 ± 16.48**	0.79 ± 0.29	**0.55 ± 0.37**	0.67 ± 0.34	0.64 ± 0.21	0.84 ± 0.16
StimN	3DFD	72.46 ± 18.30	0.75 ± 0.28	**0.59 ± 0.36**	0.66 ± 0.35	0.64 ± 0.22	0.82 ± 0.21
StimN	FixDur	50.54 ± 14.48	0.49 ± 0.23	0.47 ± 0.26	0.55 ± 0.31	0.47 ± 0.17	0.56 ± 0.19
StimN	Combined	**74.04 ± 18.85**	0.78 ± 0.28	0.58 ± 0.39	0.67 ± 0.35	0.65 ± 0.24	0.79 ± 0.26

Note. Within each stimulus group, boldface indicates the feature set with the highest values for both Accuracy and Specificity across the three feature sets. In cases of tied highest values, boldface was assigned to the feature set that also showed the highest value for the other metric.

**Table A8:** Classification performance of RBF classifier for N vs. ER using $M_{P}^{s}$ = 10 longest fixation-duration locations (10-fold cross-validation), with balanced data for each class (n = 16).

Stimulus Set	Feature Set	Accuracy	Sensitivity	Specificity	Precision	F1	AUC
A	3DFD	86.75 ± 17.07	0.83 ± 0.30	**0.72 ± 0.38**	0.75 ± 0.36	0.75 ± 0.21	0.93 ± 0.11
A	FixDur	74.67 ± 15.63	0.70 ± 0.29	0.68 ± 0.38	0.72 ± 0.33	0.67 ± 0.21	0.89 ± 0.12
A	Combined	**86.92 ± 17.99**	0.86 ± 0.31	0.70 ± 0.42	0.75 ± 0.36	0.75 ± 0.25	0.98 ± 0.03
B	3DFD	97.50 ± 5.62	0.89 ± 0.31	**0.88 ± 0.32**	0.87 ± 0.32	0.88 ± 0.21	1.00 ± 0.00
B	FixDur	80.21 ± 16.17	0.71 ± 0.34	0.70 ± 0.42	0.73 ± 0.38	0.68 ± 0.24	0.89 ± 0.12
B	Combined	**97.50 ± 7.91**	0.90 ± 0.32	0.86 ± 0.33	0.86 ± 0.33	0.87 ± 0.21	1.00 ± 0.00
C	3DFD	88.61 ± 14.01	0.82 ± 0.31	0.76 ± 0.38	0.78 ± 0.34	0.77 ± 0.20	0.98 ± 0.04
C	FixDur	84.44 ± 12.78	0.77 ± 0.31	**0.78 ± 0.35**	0.79 ± 0.33	0.76 ± 0.20	0.97 ± 0.06
C	Combined	**91.67 ± 11.42**	0.87 ± 0.31	0.77 ± 0.36	0.80 ± 0.33	0.80 ± 0.20	1.00 ± 0.01
D	3DFD	96.67 ± 10.54	0.87 ± 0.32	0.90 ± 0.32	0.90 ± 0.32	0.89 ± 0.23	1.00 ± 0.00
D	FixDur	96.67 ± 10.54	0.85 ± 0.34	0.90 ± 0.32	0.90 ± 0.32	0.87 ± 0.22	1.00 ± 0.00
D	Combined	**100.00 ± 0.00**	0.90 ± 0.32	**0.90 ± 0.32**	0.90 ± 0.32	0.90 ± 0.21	1.00 ± 0.00
E	3DFD	96.67 ± 10.54	0.87 ± 0.32	0.90 ± 0.32	0.90 ± 0.32	0.89 ± 0.23	1.00 ± 0.00
E	FixDur	**100.00 ± 0.00**	0.90 ± 0.32	**0.90 ± 0.32**	0.90 ± 0.32	0.90 ± 0.21	1.00 ± 0.00
E	Combined	**100.00 ± 0.00**	0.90 ± 0.32	**0.90 ± 0.32**	0.90 ± 0.32	0.90 ± 0.21	1.00 ± 0.00
StimP	3DFD	71.01 ± 16.95	0.77 ± 0.28	0.55 ± 0.41	0.66 ± 0.35	0.61 ± 0.22	0.80 ± 0.20
StimP	FixDur	63.20 ± 12.80	0.54 ± 0.25	**0.63 ± 0.30**	0.63 ± 0.32	0.57 ± 0.16	0.73 ± 0.16
StimP	Combined	**74.21 ± 18.18**	0.79 ± 0.29	0.59 ± 0.42	0.70 ± 0.36	0.65 ± 0.24	0.86 ± 0.19
StimN	3DFD	**72.54 ± 14.77**	0.76 ± 0.28	**0.58 ± 0.37**	0.67 ± 0.34	0.63 ± 0.18	0.81 ± 0.18
StimN	FixDur	53.71 ± 11.58	0.50 ± 0.23	0.52 ± 0.28	0.58 ± 0.34	0.49 ± 0.14	0.62 ± 0.16
StimN	Combined	72.29 ± 15.99	0.76 ± 0.28	0.58 ± 0.40	0.67 ± 0.35	0.63 ± 0.20	0.80 ± 0.19

Note. Within each stimulus group, boldface indicates the feature set with the highest values for both Accuracy and Specificity across the three feature sets. In cases of tied highest values, boldface was assigned to the feature set that also showed the highest value for the other metric.

**Table A9:** Classification performance of RBF classifier for EC vs. ER using $M_{P}^{s}\;$= 10 longest fixation-duration locations (10-fold cross-validation), with balanced data for each class (n = 16).

Stimulus Set	Feature Set	Accuracy	Sensitivity	Specificity	Precision	F1	AUC
A	3DFD	92.33 ± 12.67	0.86 ± 0.31	0.79 ± 0.36	0.82 ± 0.34	0.82 ± 0.22	0.94 ± 0.12
A	FixDur	77.25 ± 14.18	0.66 ± 0.28	0.79 ± 0.30	0.75 ± 0.32	0.70 ± 0.18	0.87 ± 0.09
A	Combined	**93.08 ± 10.74**	0.86 ± 0.31	**0.82 ± 0.34**	0.84 ± 0.33	0.84 ± 0.22	0.99 ± 0.04
B	3DFD	95.83 ± 10.58	0.89 ± 0.31	0.85 ± 0.34	0.85 ± 0.34	0.86 ± 0.22	0.98 ± 0.06
B	FixDur	88.96 ± 13.75	0.78 ± 0.31	0.85 ± 0.32	0.85 ± 0.34	0.82 ± 0.23	0.91 ± 0.16
B	Combined	**96.67 ± 10.54**	0.90 ± 0.32	**0.85 ± 0.34**	0.85 ± 0.34	0.87 ± 0.22	0.99 ± 0.03
C	3DFD	93.89 ± 11.25	0.86 ± 0.32	0.85 ± 0.34	0.85 ± 0.34	0.85 ± 0.22	0.96 ± 0.12
C	FixDur	91.11 ± 12.61	0.83 ± 0.31	0.85 ± 0.34	0.85 ± 0.34	0.83 ± 0.22	0.96 ± 0.08
C	Combined	**94.44 ± 12.00**	0.88 ± 0.32	**0.85 ± 0.34**	0.85 ± 0.34	0.86 ± 0.23	0.97 ± 0.08
D	3DFD	**96.67 ± 10.54**	0.90 ± 0.32	**0.85 ± 0.34**	0.85 ± 0.34	0.87 ± 0.22	0.94 ± 0.18
D	FixDur	90.83 ± 14.93	0.77 ± 0.42	0.85 ± 0.34	0.75 ± 0.42	0.80 ± 0.27	0.94 ± 0.18
D	Combined	**96.67 ± 10.54**	0.90 ± 0.32	**0.85 ± 0.34**	0.85 ± 0.34	0.87 ± 0.22	0.94 ± 0.18
E	3DFD	**96.67 ± 10.54**	0.90 ± 0.32	**0.85 ± 0.34**	0.85 ± 0.34	0.87 ± 0.22	1.00 ± 0.00
E	FixDur	**96.67 ± 10.54**	0.90 ± 0.32	**0.85 ± 0.34**	0.85 ± 0.34	0.87 ± 0.22	1.00 ± 0.00
E	Combined	**96.67 ± 10.54**	0.90 ± 0.32	**0.85 ± 0.34**	0.85 ± 0.34	0.87 ± 0.22	1.00 ± 0.00
StimP	3DFD	79.12 ± 9.38	0.67 ± 0.24	0.74 ± 0.32	0.74 ± 0.32	0.70 ± 0.15	0.83 ± 0.08
StimP	FixDur	61.97 ± 10.65	0.51 ± 0.24	0.66 ± 0.28	0.64 ± 0.32	0.57 ± 0.14	0.70 ± 0.13
StimP	Combined	**85.88 ± 9.60**	0.76 ± 0.28	**0.78 ± 0.34**	0.79 ± 0.32	0.76 ± 0.18	0.92 ± 0.05
StimN	3DFD	77.08 ± 16.15	0.69 ± 0.26	0.72 ± 0.36	0.72 ± 0.34	0.68 ± 0.19	0.84 ± 0.14
StimN	FixDur	58.54 ± 8.56	0.46 ± 0.19	0.64 ± 0.25	0.62 ± 0.30	0.54 ± 0.11	0.65 ± 0.07
StimN	Combined	**81.67 ± 14.86**	0.72 ± 0.26	**0.76 ± 0.36**	0.76 ± 0.34	0.72 ± 0.19	0.90 ± 0.10

Note. Within each stimulus group, boldface indicates the feature set with the highest values for both Accuracy and Specificity across the three feature sets. In cases of tied highest values, boldface was assigned to the feature set that also showed the highest value for the other metric.

## Appendix C

### Mathematical Form of Linear Mixed-Effects Modelling Equations

Fixation-duration model:

(A1) $$\begin{array}{c} {FixationDuration}_{ij}\\ \;\;\;\;\;\;\;\;\;\;\;\;\;\;\;\;\;\;\;\;\;\;\;\;\;\;=\;\beta_{0}\;+\;\beta_{1}{EC}_{i}\;+\;\beta_{2}{ER}_{i}\;+\;\beta_{3}{Pathology}_{j}\;+\;\beta_{4}({EC}_{i}\;\times \;{Pathology}_{j})\\ \;\;\;\;\;\;\;\;\;\;\;\;\;\;\;\;\;\;\;\;\;\;\;\;\;\;+\;\beta_{5}({ER}_{i}\;\times \;{Pathology}_{j})\;+\;u_{i}\;+\;v_{j}\;+\;\varepsilon_{ij} \end{array}$$

Here, $i\;\in \;\left\{1,2,\cdots ,N_{participants}\right\}$ and $j\;\in \;\left\{1,2,\cdots ,N_{stimuli}\right\}$ denote indices for participant (Subject) and stimulus (StimuliID), respectively. $N_{participants}$ is the total number of participants, and $N_{stimuli}$ is the total number of stimuli. The random intercepts are defined as:

(A2) $$u_{i}\sim N\left(0,\sigma_{Subject}^{2}\right)\;v_{j}\sim N\left(0,\sigma_{StimuliID}^{2}\right)\;\varepsilon_{ij}\sim N\left(0,\sigma^{2}\right)$$

where:

$u_{i}$ represents the participant-specific deviation from the grand intercept.

$v_{j}$ represents the stimulus-specific deviation from the grand intercept.

$\varepsilon_{ij}$ represents the residual error for the observation from participant i viewing stimulus j.

The variance components have the following interpretations:

- $\sigma_{Subject}^{2}$ is the variance of the participant-level random intercepts. It quantifies how much baseline fixation duration varies between participants after accounting for the fixed effects.
- $\sigma_{StimuliID}^{2}$ is the variance of the stimulus-level random intercepts. It quantifies how much baseline fixation duration varies between stimuli after accounting for the fixed effects.
- $\sigma^{2}$ is the residual variance, representing within-participant × stimulus variability not explained by either the fixed effects or the random intercepts.
  All random components are assumed to be mutually independent.

3DFD model:

(A3) $$\begin{array}{cc} {Mean3DFD}_{ij}= & \gamma_{0}+\gamma_{1}{EC}_{i}+\gamma_{2}{ER}_{i}+\gamma_{3}{Pathology}_{j}\\ & +\gamma_{4}({EC}_{i}\times {Pathology}_{j})+\gamma_{5}({ER}_{i}\times {Pathology}_{j})+u_{i}\\ & +v_{j}+\varepsilon_{ij} \end{array}$$

The notation, indexing, and random-effects structure for the 3DFD model are identical to those described for the fixation-duration model (Equations (A1) and (A2)). The coefficients $\gamma_{k}$ correspond to fixed effects for Mean3DFD rather than fixation duration.

## Appendix D

**Table A10:** Dataset size for N vs. EC classification. Participant count: N (*N_p_* = 29), EC (*N_p_* = 24), total = 53 participants.

Stimulus Group	No. of Stimuli	Total Classifier Samples	Approx. Test Fold Size	Approx. Training Size
A	10	530	53	477
B	4	212	21	191
C	3	159	16	143
D	1	53	5	48
E	1	53	5	48
StimP	19	1007	101	906
StimN	20	1060	106	954
Full dataset	39	2067	207	1860

**Table A11:** Dataset size for N vs. ER classification. Participant count: N (*N_p_* = 29), ER (*N_p_* = 16), total = 45 participants.

Stimulus Group	No. of Stimuli	Total Classifier Samples	Approx. Test Fold Size	Approx. Training Size
A	10	450	45	405
B	4	180	18	162
C	3	135	14	121
D	1	45	5	40
E	1	45	5	40
StimP	19	855	86	769
StimN	20	900	90	810
Full dataset	39	1755	176	1579

**Table A12:** Dataset size for EC vs. ER classification. Participant count: EC (*N_p_* = 24), ER (*N_p_* = 16), total = 40 participants.

Stimulus Group	No. of Stimuli	Total Classifier Samples	Approx. Test Fold Size	Approx. Training Size
A	10	400	40	360
B	4	160	16	144
C	3	120	12	108
D	1	40	4	36
E	1	40	4	36
StimP	19	760	76	684
StimN	20	800	80	720
Full dataset	39	1560	156	1404
