# Supplementary material for: Characterizing Visual Neurosurgical Expertise in Brain MRI Visualization Using Eye-Tracking and 3D Fractal Dimension Analysis
Source: J Eye Mov Res. 2026 Jun 2;19(3):62. doi: 10.3390/jemr19030062 (PMC13301434; doi:10.3390/jemr19030062)
Supplement: Supplementary file 1 [file jemr-19-00062-s001.zip › jemr-4253847-supplementary/Supplementary files/SupplementaryMaterial_NSA_Ver20260327.pdf]

Journal of
Eye Movement Research

MDPI

Article

Characterizing visual neurosurgical expertise in brain MRI visualization using eye-tracking and 3D fractal dimension analysis

Poonam Kumari[1,*], Ghasem Azemi[1], Carlo Russo[1], Antonio Di Ieva[1,*]

[1] Computational NeuroSurgery (CNS) Lab, Macquarie Medical School, Faculty of Medicine, Health and Human Sciences, Macquarie University, 75 Talavera Road, Macquarie Park, 2109, NSW, Australia; ghasem.azemi@mq.edu.au; carlo.russo@mq.edu.au
* Correspondence: PK: poonam.kumari@hdr.mq.edu.au; ADI: antonio.diieva@mq.edu.au

S1. Normal and pathological brain MRI stimuli images

Folder name where images are located:

1_StimuliImages\Brain_stimuli

S2. Mixed-effects modelling – MATLAB data processing pipeline

S2.1 Step 1. Matlab code (.m), its input, output and its location*

S2.1.1 Matlab code (*.m) and its location

MATLAB file:
Step1_createDataMatrix_first_N_max.m

Folder name where the MATLAB file is located:
2_MixedEffectsModelling

S2.1.2 Input data to the MATLAB code

S2.1.2.1 Input data (*.mat file) to the MATLAB code

Name of file:
dataMatrix_stimuli3DFD_withinOneROI_And_FixDur.mat

Folder name where the MATLAB data file is located:

2_MixedEffectsModelling

S2.1.2.2 User input to the MATLAB code

first_N_max

In the below excerpt from the MATLAB code, first_N_max = 10.

```
% Choose the number of maximum fixation points as "first_N_max"

first_N_max = 10;
```

S2.1.3. Output from the MATLAB code is a *.mat file

Name of file:
dataMatrix_stimuli3DFD_withinOneROI_And_FixDur_upto_first_10_max.mat

Folder name where the MATLAB data file is located:

2_MixedEffectsModelling

S2.2 Step 2. Matlab code (.m), its input, output and its location*

S2.2.1 Matlab code (*.m) and its location

MATLAB file:
Step2_MixEffectModel_3DFD_FixDur_first_N_max.m

Folder name where the MATLAB file is located:
2_MixedEffectsModelling

S2.2.2 Input data to the MATLAB code

S2.2.2.1 Input data (*.mat file) to the MATLAB code

Name of file:
dataMatrix_stimuli3DFD_withinOneROI_And_FixDur_upto_first_10_max.mat

Folder name where the MATLAB data file is located:

2_MixedEffectsModelling

S2.2.2.2 User input to the MATLAB code

`dirPathToSaveOutputs`

In the excerpt below from the MATLAB code, the directory name to export APA-style results Tables to excel spreadsheet(s) is located in the current local path of your computer.

```
% Directory name to export APA-style Tables to excel spreadsheet(s)
dirPathToSaveOutputs = strcat(pwd,'\Output\First',num2str(first_N_max),'Max\');
```

S2.2.3. Output from the MATLAB code (*.m)

Two files shall be generated for each of the stimulus group A, B, C, D, E and All, one for each of fixation duration and 3DFD.

Example: Name of the excel files generated by the MATLAB code for stimulus group A, for fixation duration and 3DFD:

MixedModelResults_UptoMax10_withinOneROI_FixDur_ImageType_A.xlsx
MixedModelResults_UptoMax10_withinOneROI_3DFD_ImageType_A.xlsx

In addition, the below excel two files shall be generated corresponding to Table 2 and Table 3:

Table2_MixedModelResults_UptoMax10_FixDur_ImageType.xlsx

Table3_MixedModelResults_UptoMax10_3DFD_ImageType.xlsx

Folder name where the spreadsheets are located:

2_MixedEffectsModelling\Output\First10Max

S2.3 Step 3. Matlab code (.m), its input, output and its location*

S2.3.1 Matlab code (*.m) and its location

MATLAB file:

Step3_WhiskerPlots_first_N_max.m

Folder name where the MATLAB file is located:

2_MixedEffectsModelling

S2.3.2 Input data to the MATLAB code

S2.3.2.1 Input data (*.mat file) to the MATLAB code

Name of file:

dataMatrix_stimuli3DFD_withinOneROI_And_FixDur_upto_first_10_max.mat

Folder name where the MATLAB data file is located:

2_MixedEffectsModelling

S2.3.2. Output from the MATLAB code (*.m)

Whisker plots of Figure 4 and Figure 5 of the main draft.

S3. Machine learning – MATLAB data processing pipeline Mixed-effects modelling

S3.1 Step 1. Matlab code (.m), its input, output and its location*

S3.1.1 Matlab code (*.m) and its location

MATLAB file:

Step1_MachineLearningClassifier_GroupKfold.m

Folder name where the MATLAB file is located:

3_MachineLearning

S3.1.2 Input data to the MATLAB code

S3.1.2.1 User input to the MATLAB code

In the below excerpt from the MATLAB code, the directory name "`dir_path_local`" from where the *.mat file is read is the local path of your computer. In the below example, the local path of data is in OneDrive.

```
% Define the local directory path for data files
dir_path_local = 'Define the local directory path for data files';
```

```
% Place the below directory path in your local path
% 'MDPI_Sensors_Journal\Final\DataProcessing_NSA\2_MixedEffectsModel-
ling\'
dir_path = strcat(dir_path_local,'…\2_MixedEffectsModelling\');
```

first_N_max

In the below excerpt from the MATLAB code, first_N_max = 10.

```
% Choose the number of maximum fixation points as "first_N_max"

first_N_max = 10;
```

In the excerpt below from the MATLAB code, the array corresponding to the number of K-folds, the number of features, and the feature name are used as inputs.

```
number_Kfold = 2:10; % An array of k-fold-cross-validation
    noOfFeatures = first_N_max; % Number of features ==> first N maximum chosen for 3DFD
and Fixation Duration
    featureName = '3DFD_FixDur' % Feature name ==> {'3DFD','FixDur','3DFD_FixDur'}
```

NOTE: This MATLAB code should be run 3 times, each time selecting featureName as one of the 3 parameters: `{'3DFD','FixDur','3DFD_FixDur'}.`

S3.1.2.2 Input data (*.mat file) to the MATLAB code

Name of file:
dataMatrix_stimuli3DFD_withinOneROI_And_FixDur.mat

Folder name where the MATLAB data file is located:

2_MixedEffectsModelling

S3.1.3. Output from the MATLAB code (*.m)

Name of files:
pathoSplit_GroupKFold_1st10Max_2Fet_3DFD_NewClassMetrics.mat
pathoSplit_GroupKFold_1st10Max_2Fet_FixDur_NewClassMetrics.mat
pathoSplit_GroupKFold_1st10Max_4Fet_3DFD_FixDur_NewClassMetrics.mat

Folder name where the MATLAB data files are located:

3_MachineLearning

S3.2 Step 2. Matlab code (.m), its input, output and its location*

S3.2.1 Matlab code (*.m) and its location

MATLAB file:
Step2_createMLresultsTable_forJournalPaper.m

Folder name where the MATLAB file is located :
3_MachineLearning

S3.2.2 Input data to the MATLAB code

S3.2.2.1 Input data (*.mat file) to the MATLAB code

Name of file:
dataMatrix_stimuli3DFD_withinOneROI_And_FixDur.mat

Folder name where the MATLAB data file is located:

2_MixedEffectsModelling

Name of files:
pathoSplit_GroupKFold_1st10Max_2Fet_3DFD_NewClassMetrics.mat
pathoSplit_GroupKFold_1st10Max_2Fet_FixDur_NewClassMetrics.mat
pathoSplit_GroupKFold_1st10Max_4Fet_3DFD_FixDur_NewClassMetrics.mat

Folder name where the MATLAB data files are located:

3_MachineLearning

S3.2.2.2 User input to the MATLAB code

first_N_max

In the below excerpt from the MATLAB code, first_N_max = 10.

```
% Choose the number of maximum fixation points as "first_N_max"

first_N_max = 10;
```

number_Kfold

In the screenshot below, number_Kfold = 2:10;

```
% number_Kfold
number_Kfold = 2:10; % An array of k-fold-cross-validation
```

userChosen_Kfold_forTableCreation

In the screenshot below, userChosen_Kfold_forTableCreation = 10;

```
% User chosen Kfold for Table creation
userChosen_Kfold_forTableCreation = 10;
```

In the below excerpt from the MATLAB code, the directory name to export the machine learning results Tables to excel spreadsheet(s) is the local path of my computer, with data located in OneDrive.

```
%% Folders for saving spreadsheets
foldername_MLresultsExport_K_for_Kfold = '\Output\';
% If no 'Output' folder ==> make it
if ~isfolder(strcat( dirPath, foldername_MLresultsExport_K_for_Kfold) )
    mkdir(strcat(dirPath,foldername_MLresultsExport_K_for_Kfold));
end

%% Input spreadsheet name to save classifier results
filename_MLresultsExport_K_for_Kfold = strcat( '\Output\Table_for_MLclassi-
fierResults_K_equals_',num2str(userChosen_Kfold_forTableCreation),'.xlsx'
);
if isfile(strcat(dirPath,filename_MLresultsExport_K_for_Kfold))
    % File exists ==> delete existing file
    delete(strcat(dirPath,filename_MLresultsExport_K_for_Kfold));
end
```

S3.2.3. Output from the MATLAB code (*.m)

Name of a file generated by the MATLAB code:
Table_for_MLclassifierResults_K_equals_10 .xlsx

Location of file:

3_MachineLearning\Output

S3.3 Step 3. Matlab code (.m), its input, output and its location*

S3.3.1 Matlab code (*.m) and its location

MATLAB file:
Step3_Figure_A3_A4_A5_accuracyPlots_3x3_layout_RFandSVM.m

Folder name where the MATLAB file is located:
3_MachineLearning

S3.3.2 Input data to the MATLAB code

S3.3.2.1 Input data (*.mat file) to the MATLAB code

Name of files:
pathoSplit_GroupKFold_1st10Max_2Fet_3DFD_NewClassMetrics.mat

pathoSplit_GroupKFold_1st10Max_2Fet_FixDur_NewClassMetrics.mat
pathoSplit_GroupKFold_1st10Max_4Fet_3DFD_FixDur_NewClassMetrics.mat

Folder name where the MATLAB data files are located:

3_MachineLearning

S3.3.2.2 User input to the MATLAB code

```
%% Choose "first_N_max"
first_N_max = 10;

%% Stimulus grouping for PATHOLOGICAL stimuli
% Pathological stimuli group codes
grp2_Pathology_names_codes = ["A", "B", "C", "D", "E", "All"];

% Pathological stimuli names
grp2_Pathology_names = ["Pathology: tumors", "Pathology: cerebrovas-
cular pathologies", "Pathology: others",...
                        "Pathology: inflammatory", "Pathology: mal-
fomations","Pathology: all"];

%% Stimulus grouping for NORMAL stimuli
% Normal stimuli group codes
grp1_Normal_names_codes = "N";

% Normal stimuli names
grp1_Normal_names = "Normal";
```

S3.3.3. Output from the MATLAB code (*.m)

Figures A3, A4, A5 used the Appendix B.3.1 of the paper.

S4. 3DFD maps calculation – Python and MATLAB data processing pipeline

S4.1. Python code for 3DFD calculation of stimuli images

VisualTracking_PK_NeuroPath.py
main_3DFDmap_Neuropath.py

Folder name where the Python files are located:
4_3DFDmapsCalculation

Output of Python code: `*.csv files`

Example: `3DFDs_BRAIN_1_PATH_Meningioma ax.png_Heatmap_100_10.csv`

Location of *.csv files:

4_3DFDmapsCalculation\3DFD_Stimuli_BRN40

S4.2. Converting output of Python code into a two-dimensional 3DFD images

Name of files:

`func_create3DFDMatrix.m`

Folder name where the MATLAB file is located:

4_3DFDmapsCalculation
