# Supplementary figures and images for: Characterizing Visual Neurosurgical Expertise in Brain MRI Visualization Using Eye-Tracking and 3D Fractal Dimension Analysis
Source: J Eye Mov Res. 2026 Jun 2;19(3):62. doi: 10.3390/jemr19030062 (PMC13301434; doi:10.3390/jemr19030062)

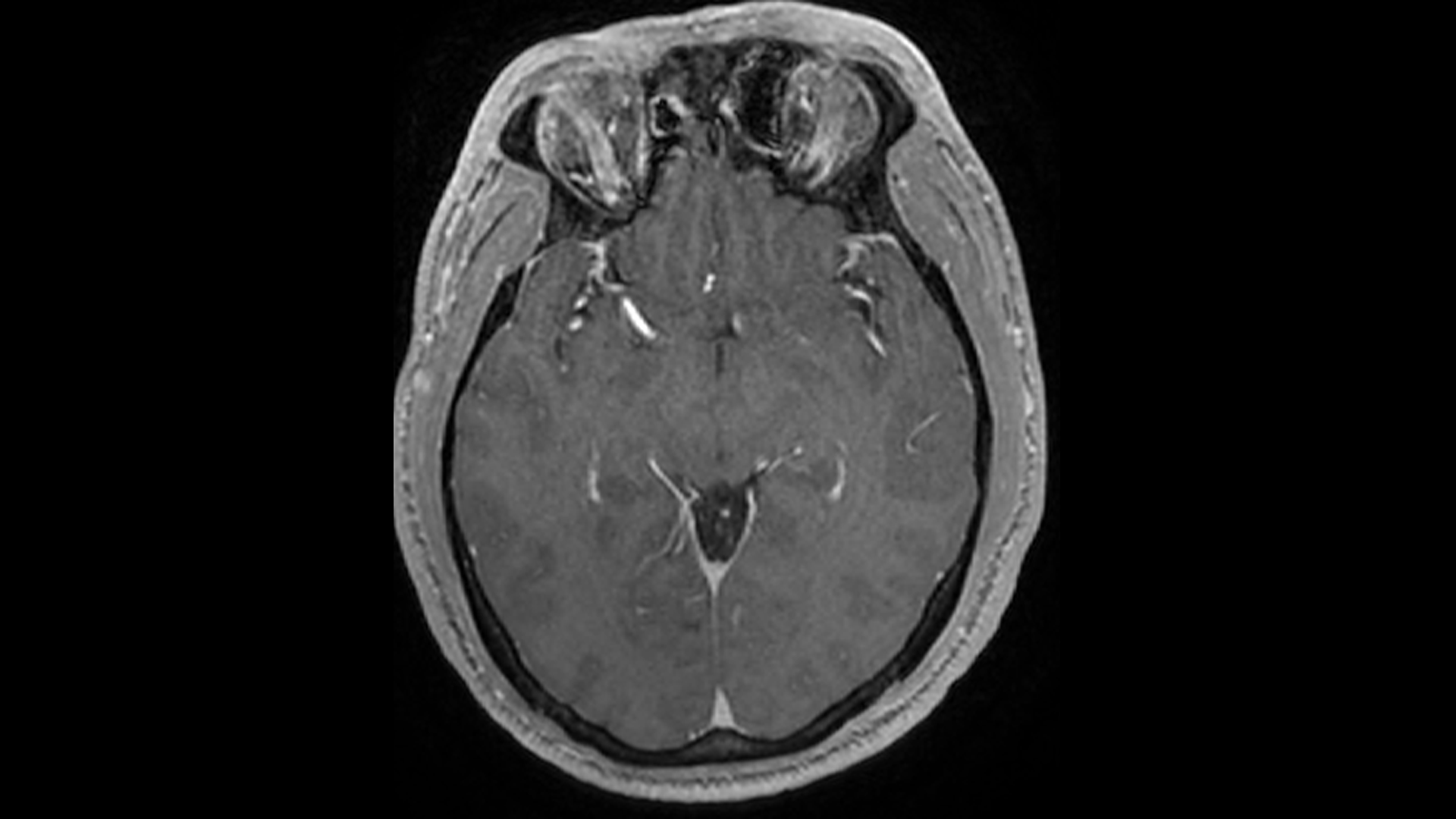

Supplement: Supplementary file 1 [file jemr-19-00062-s001.zip › jemr-4253847-supplementary/Supplementary files/1_StimuliImages/Brain_stimuli/BRAIN_10_NORMAL_Normal Ax2.png]

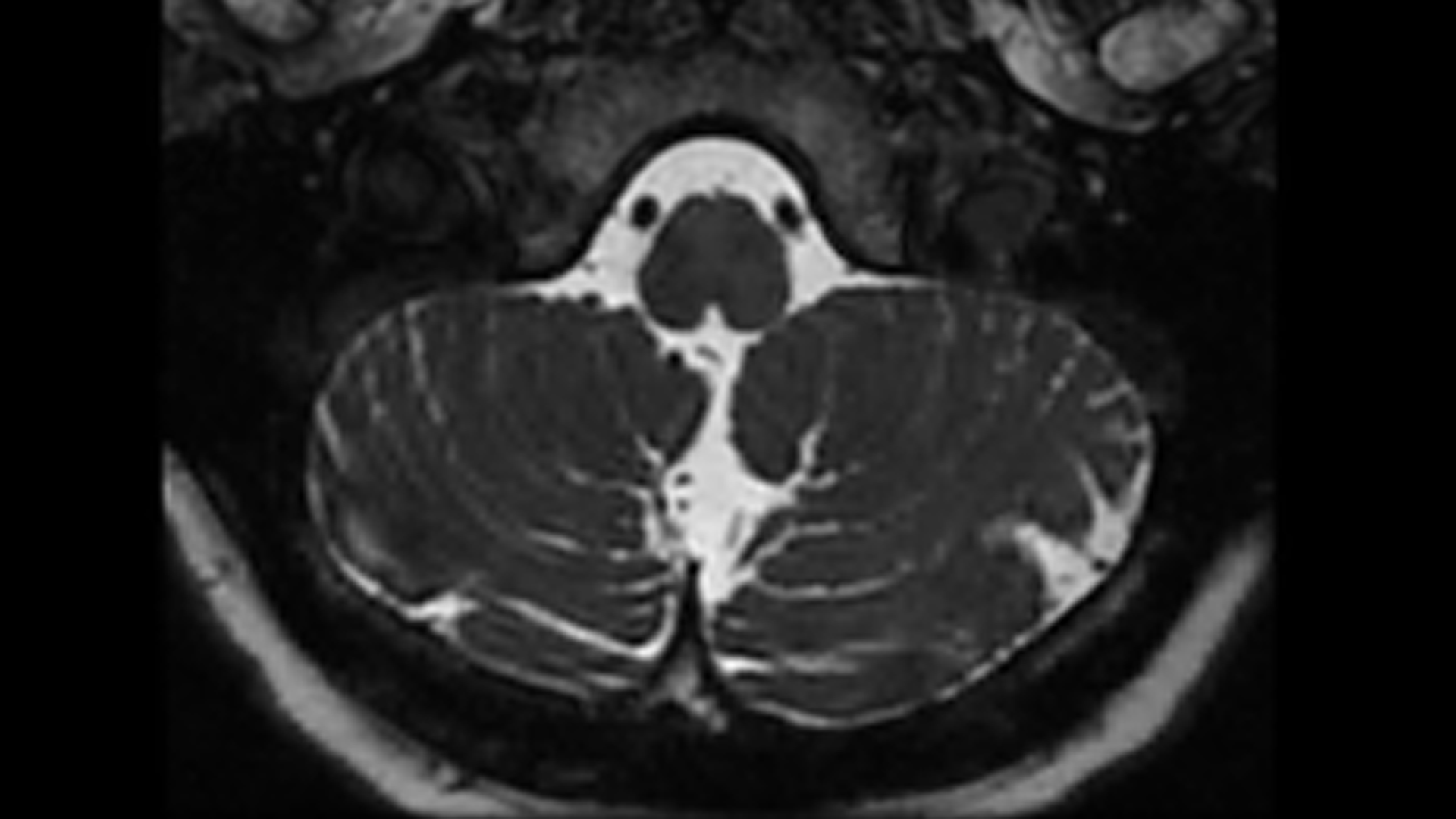

Supplement: Supplementary file 1 [file jemr-19-00062-s001.zip › jemr-4253847-supplementary/Supplementary files/1_StimuliImages/Brain_stimuli/BRAIN_11_NORMAL_Normal T2 post fossa.png]

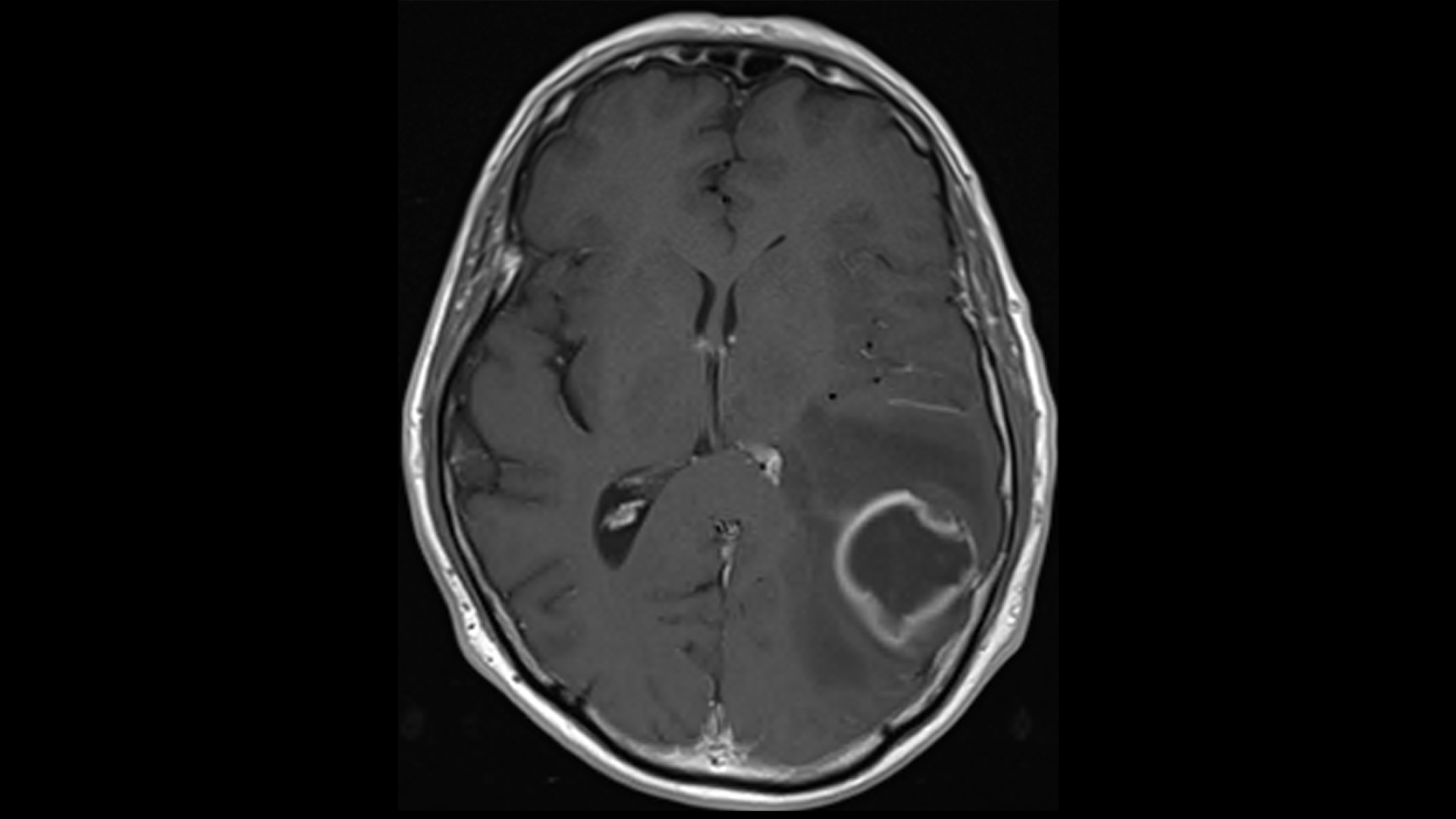

Supplement: Supplementary file 1 [file jemr-19-00062-s001.zip › jemr-4253847-supplementary/Supplementary files/1_StimuliImages/Brain_stimuli/BRAIN_12_PATH_GBM ax.png]

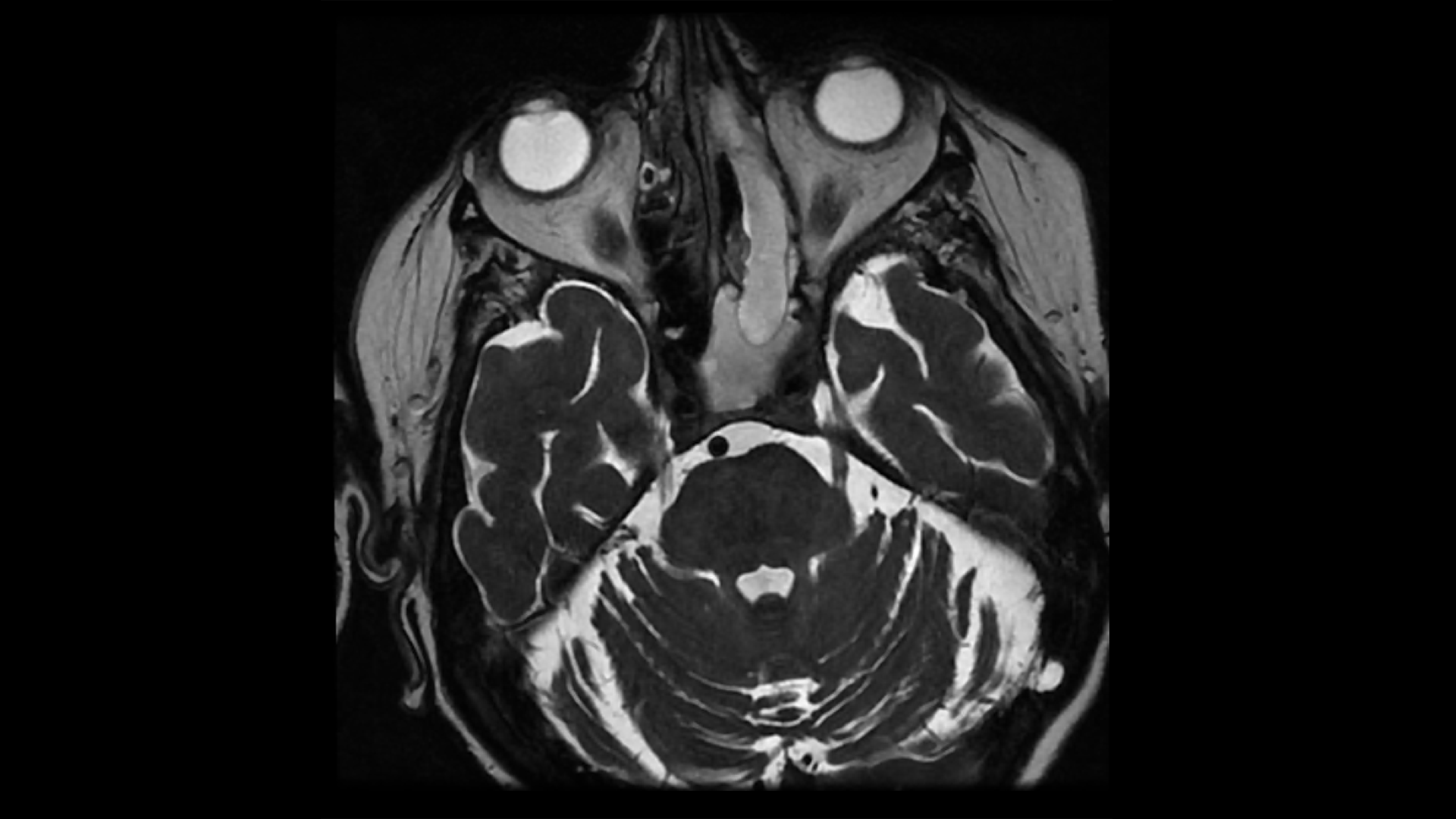

Supplement: Supplementary file 1 [file jemr-19-00062-s001.zip › jemr-4253847-supplementary/Supplementary files/1_StimuliImages/Brain_stimuli/BRAIN_13_NORMAL_T2 CPA.png]

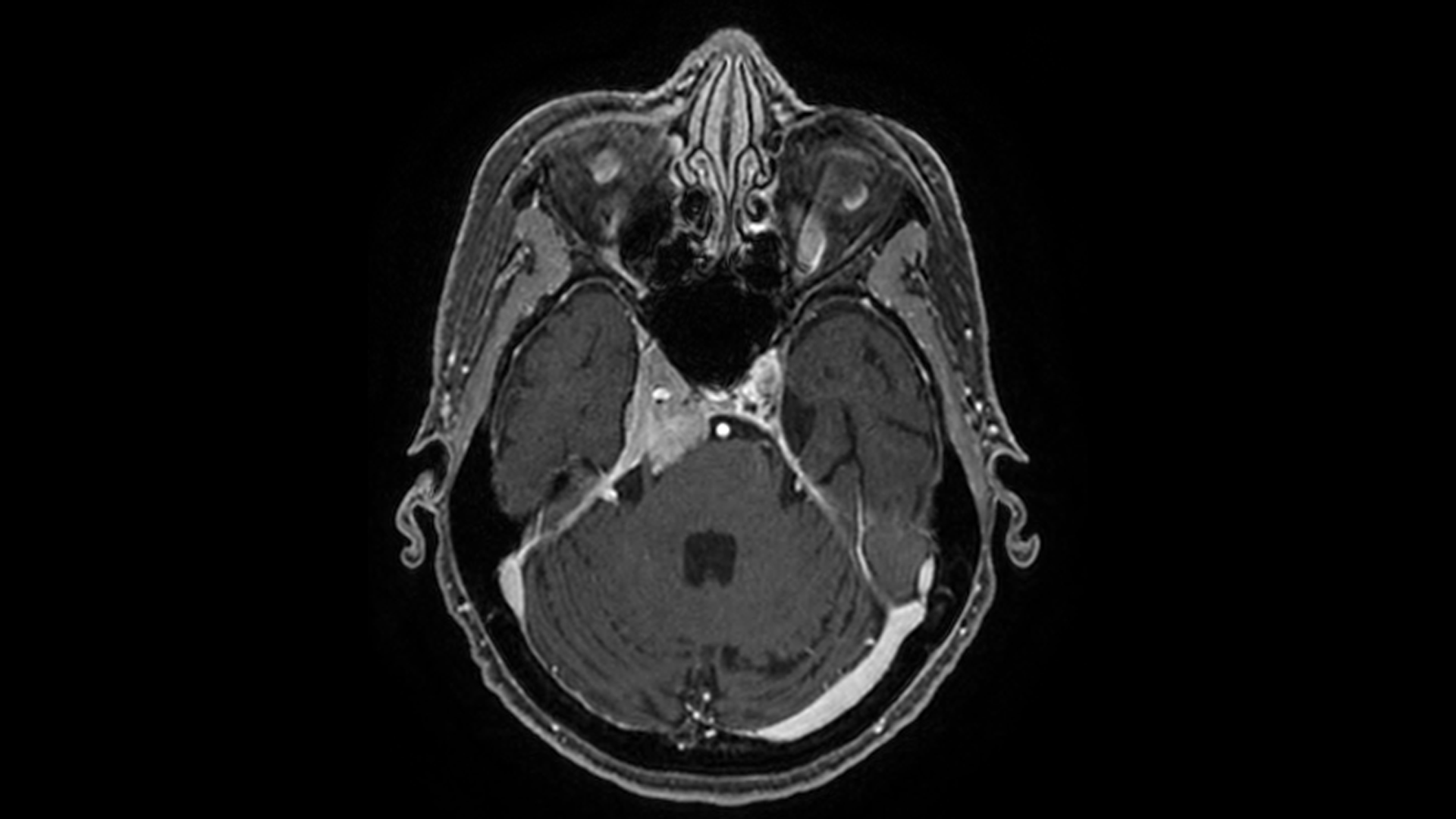

Supplement: Supplementary file 1 [file jemr-19-00062-s001.zip › jemr-4253847-supplementary/Supplementary files/1_StimuliImages/Brain_stimuli/BRAIN_14_PATH_CS meningioma.png]

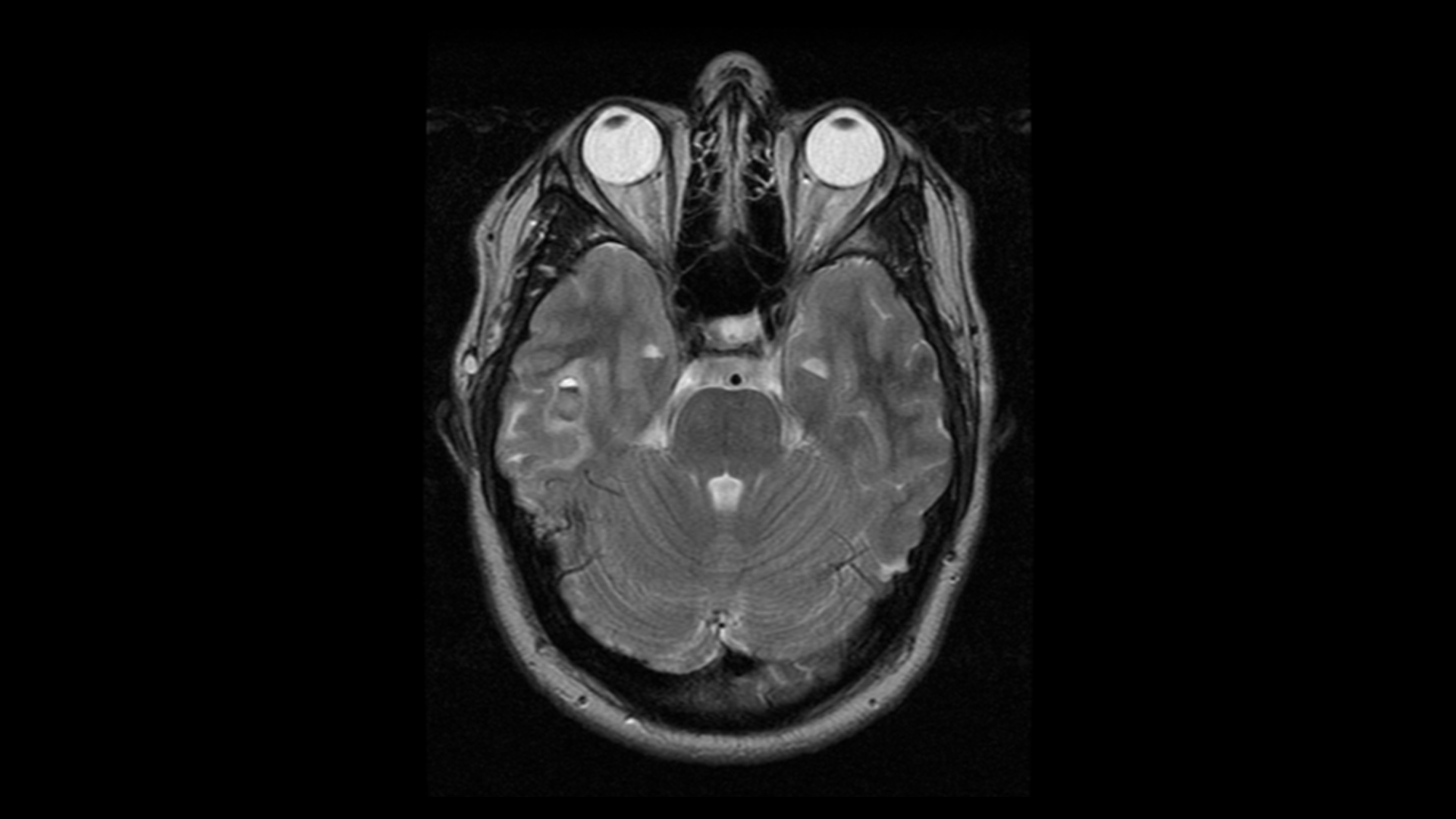

Supplement: Supplementary file 1 [file jemr-19-00062-s001.zip › jemr-4253847-supplementary/Supplementary files/1_StimuliImages/Brain_stimuli/BRAIN_15_PATH_Temporal heamatoma T2.png]

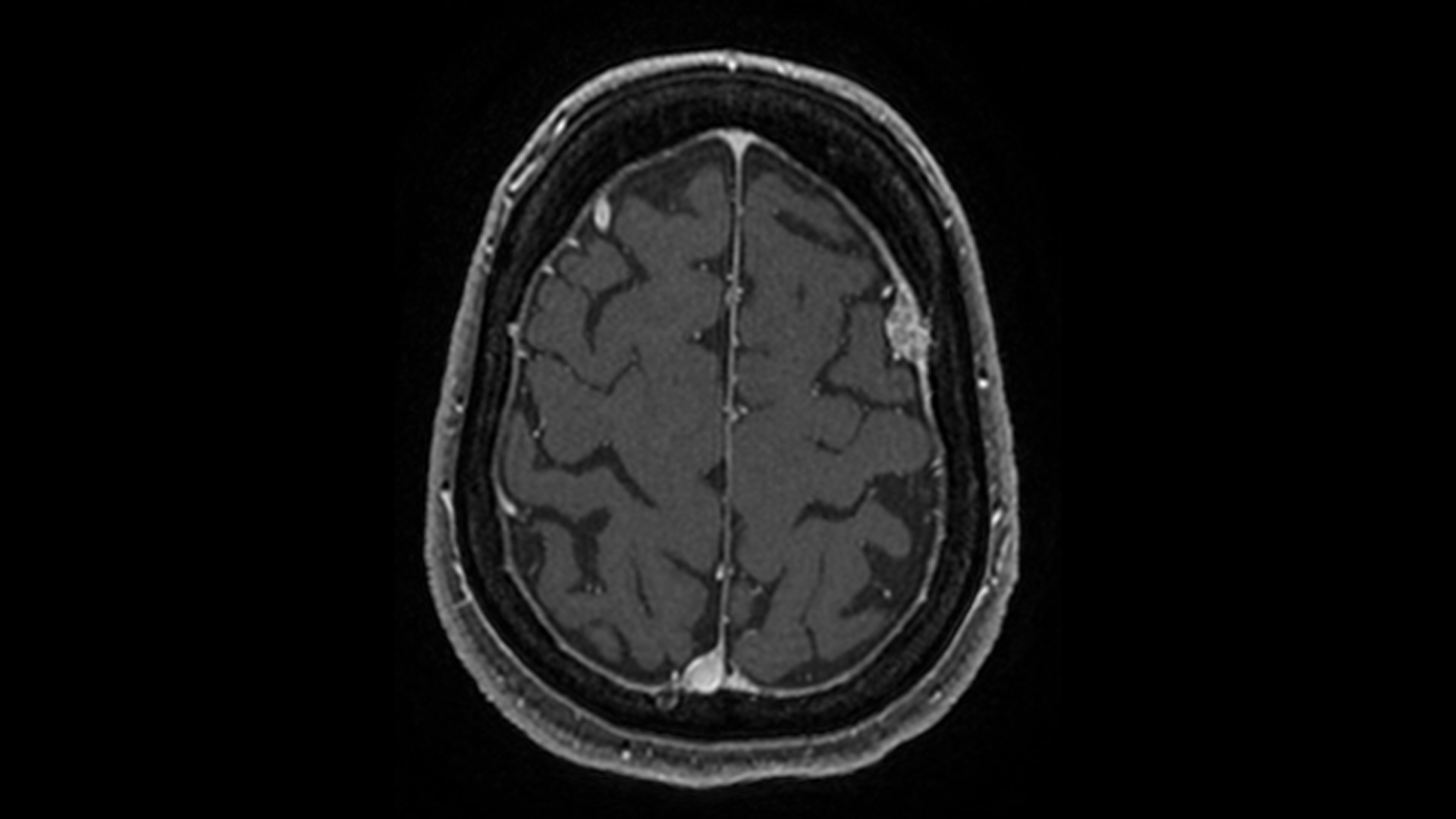

Supplement: Supplementary file 1 [file jemr-19-00062-s001.zip › jemr-4253847-supplementary/Supplementary files/1_StimuliImages/Brain_stimuli/BRAIN_16_PATH_Convexity Meningioma.png]

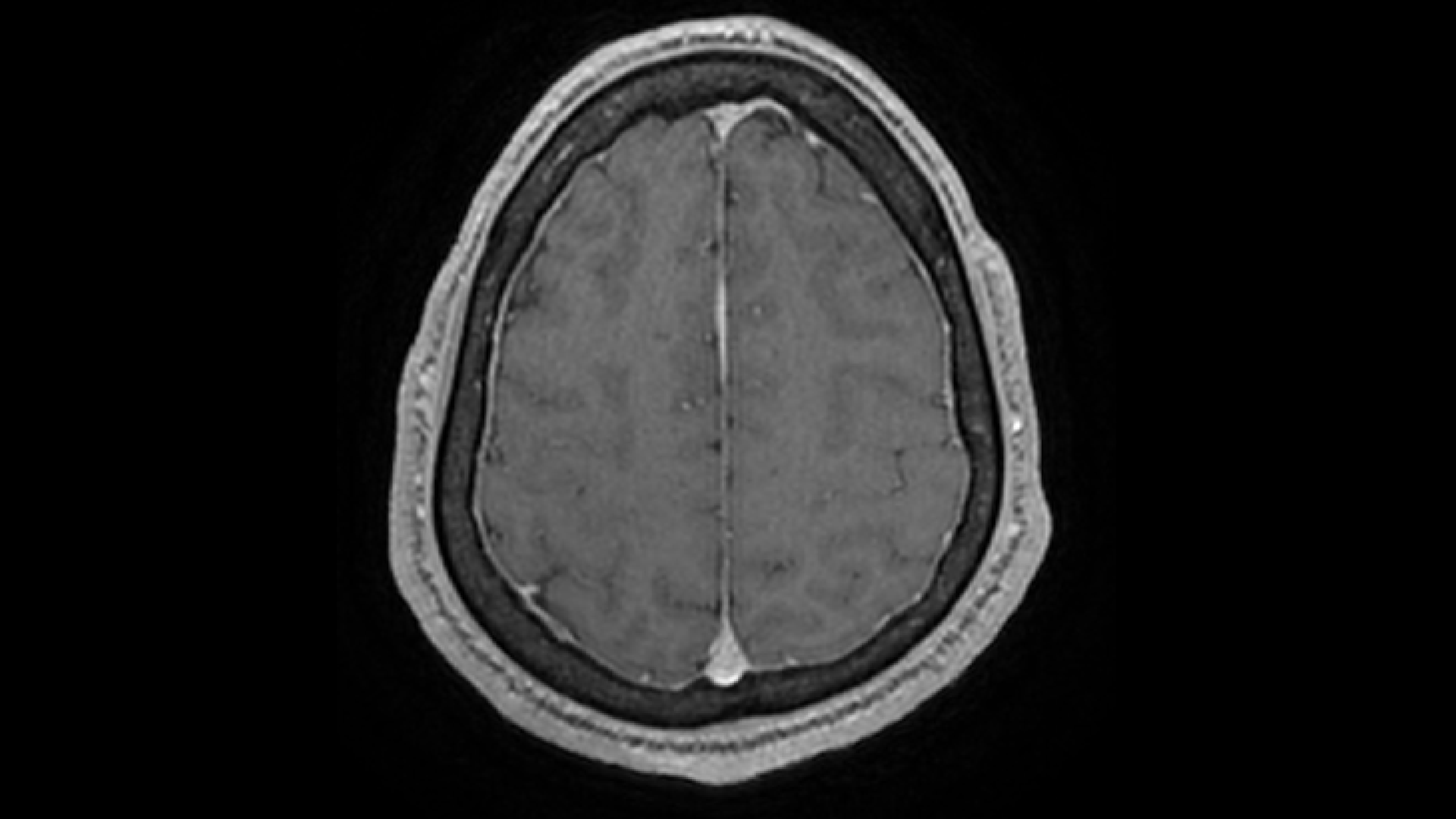

Supplement: Supplementary file 1 [file jemr-19-00062-s001.zip › jemr-4253847-supplementary/Supplementary files/1_StimuliImages/Brain_stimuli/BRAIN_17_NORMAL_Normal ax.png]

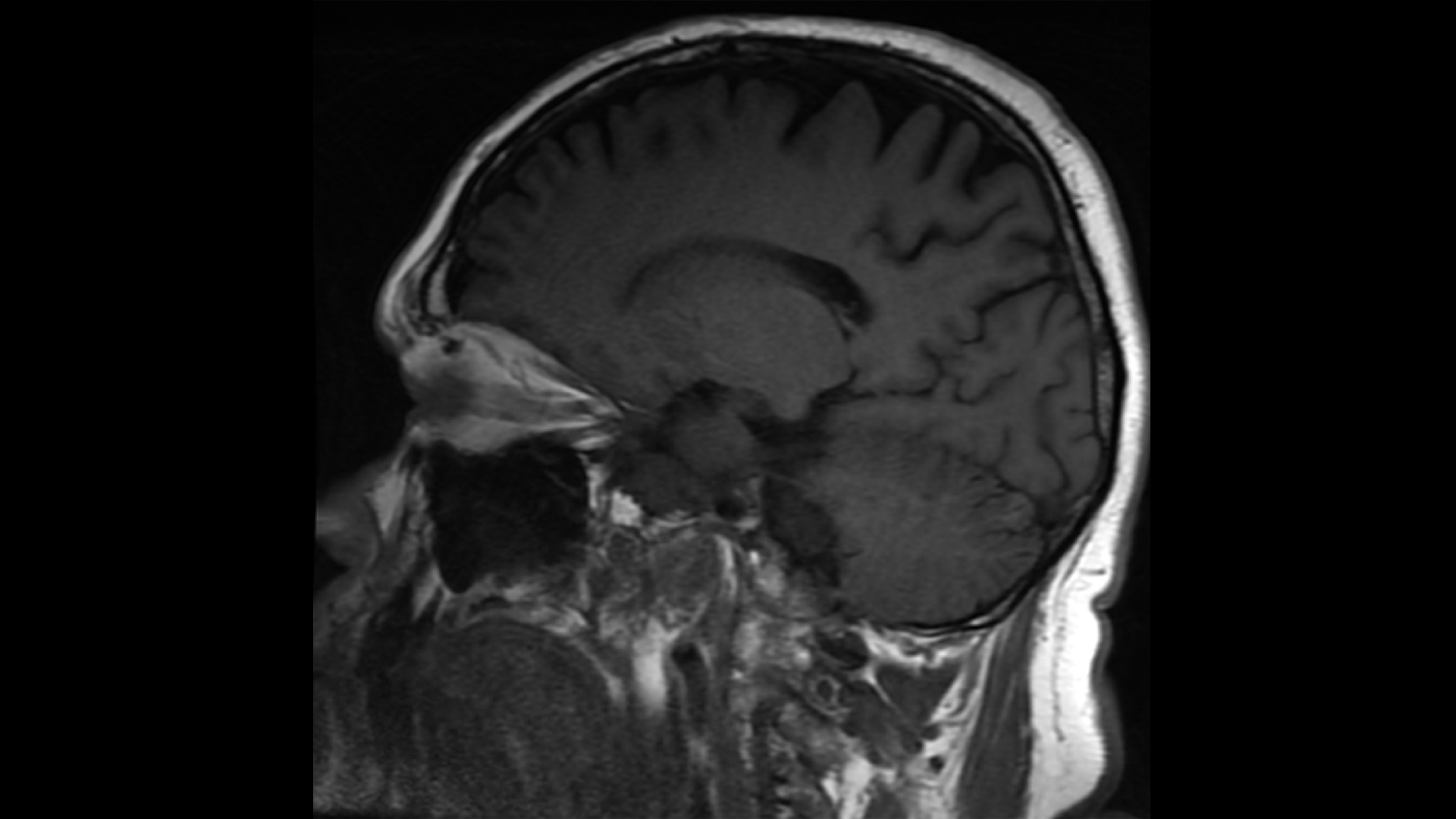

Supplement: Supplementary file 1 [file jemr-19-00062-s001.zip › jemr-4253847-supplementary/Supplementary files/1_StimuliImages/Brain_stimuli/BRAIN_18_PATH_Sag schwannoma.png]

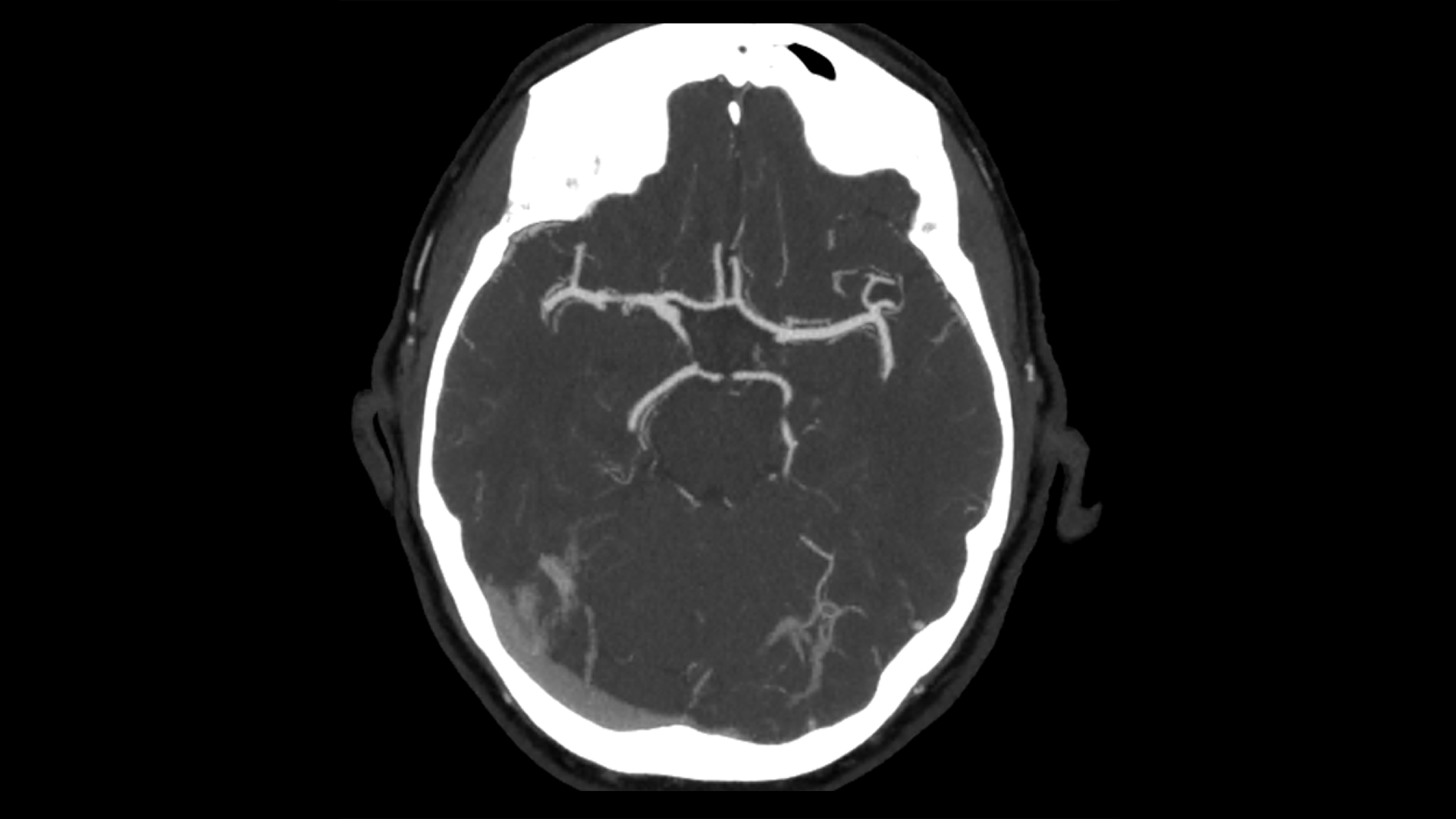

Supplement: Supplementary file 1 [file jemr-19-00062-s001.zip › jemr-4253847-supplementary/Supplementary files/1_StimuliImages/Brain_stimuli/BRAIN_19_NORMAL_Normal CTA.png]

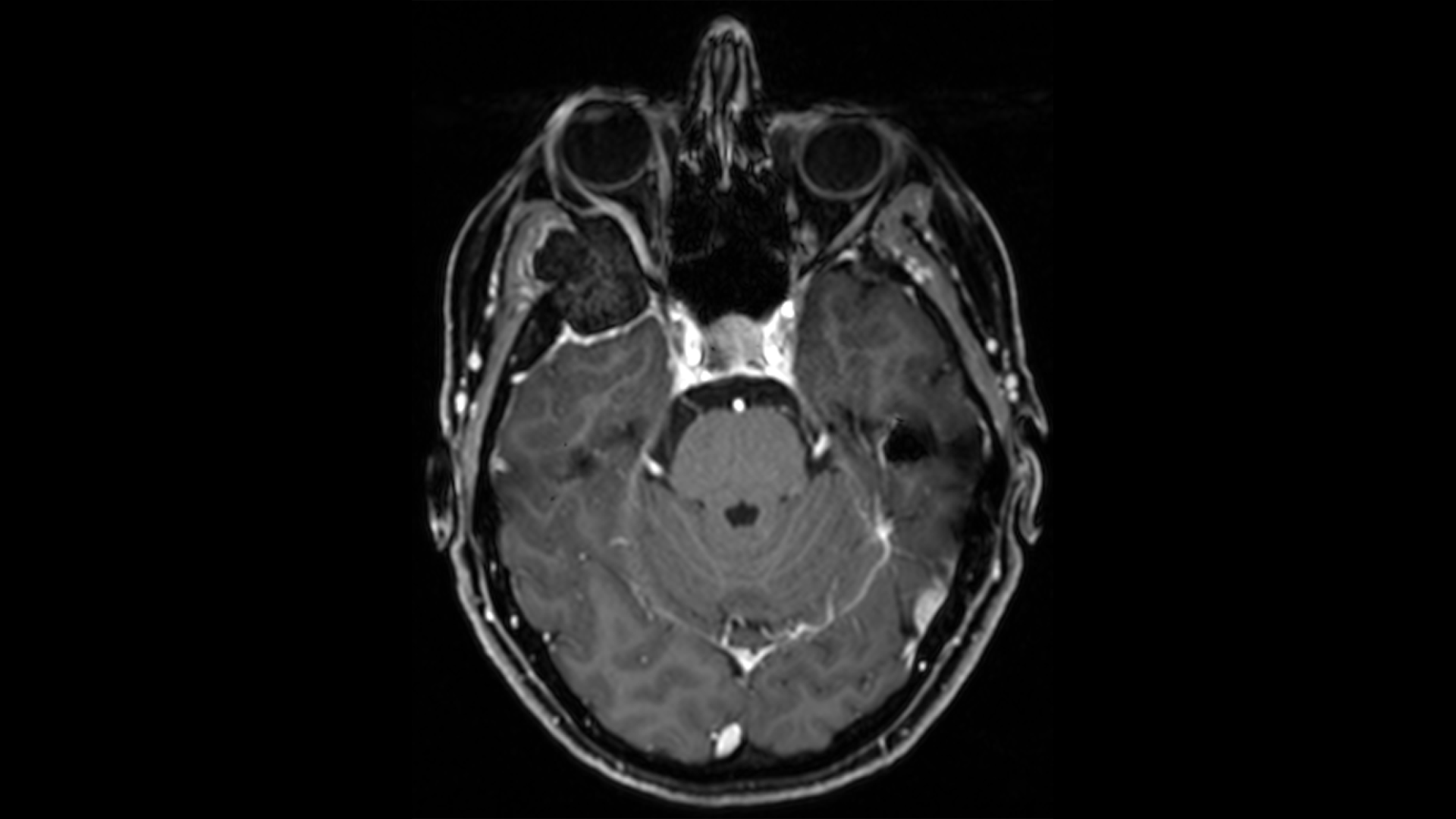

Supplement: Supplementary file 1 [file jemr-19-00062-s001.zip › jemr-4253847-supplementary/Supplementary files/1_StimuliImages/Brain_stimuli/BRAIN_1_PATH_Meningioma ax.png]

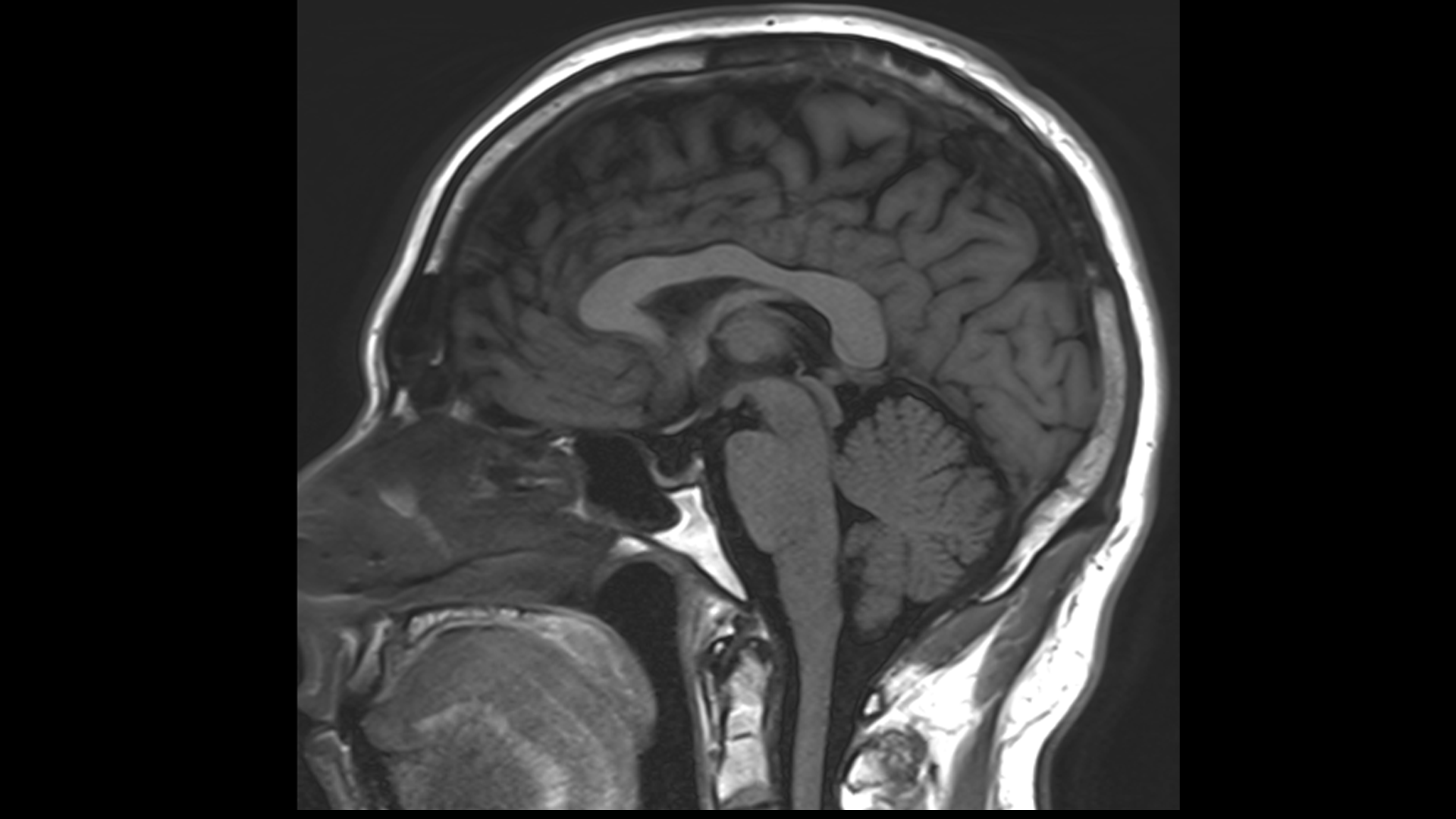

Supplement: Supplementary file 1 [file jemr-19-00062-s001.zip › jemr-4253847-supplementary/Supplementary files/1_StimuliImages/Brain_stimuli/BRAIN_20_PATH_Empty Sella.png]

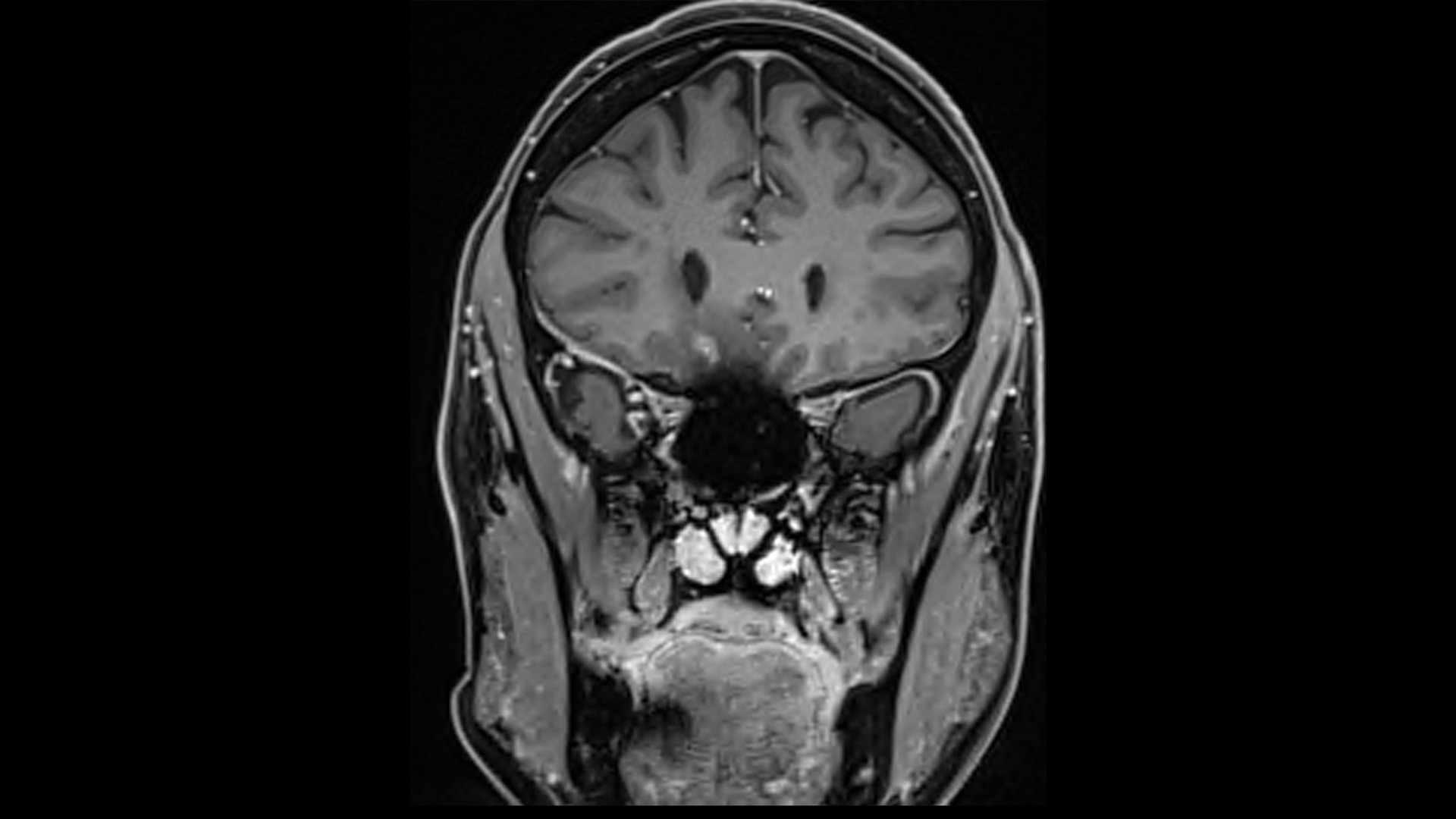

Supplement: Supplementary file 1 [file jemr-19-00062-s001.zip › jemr-4253847-supplementary/Supplementary files/1_StimuliImages/Brain_stimuli/BRAIN_21_PATH_Cor met.png]

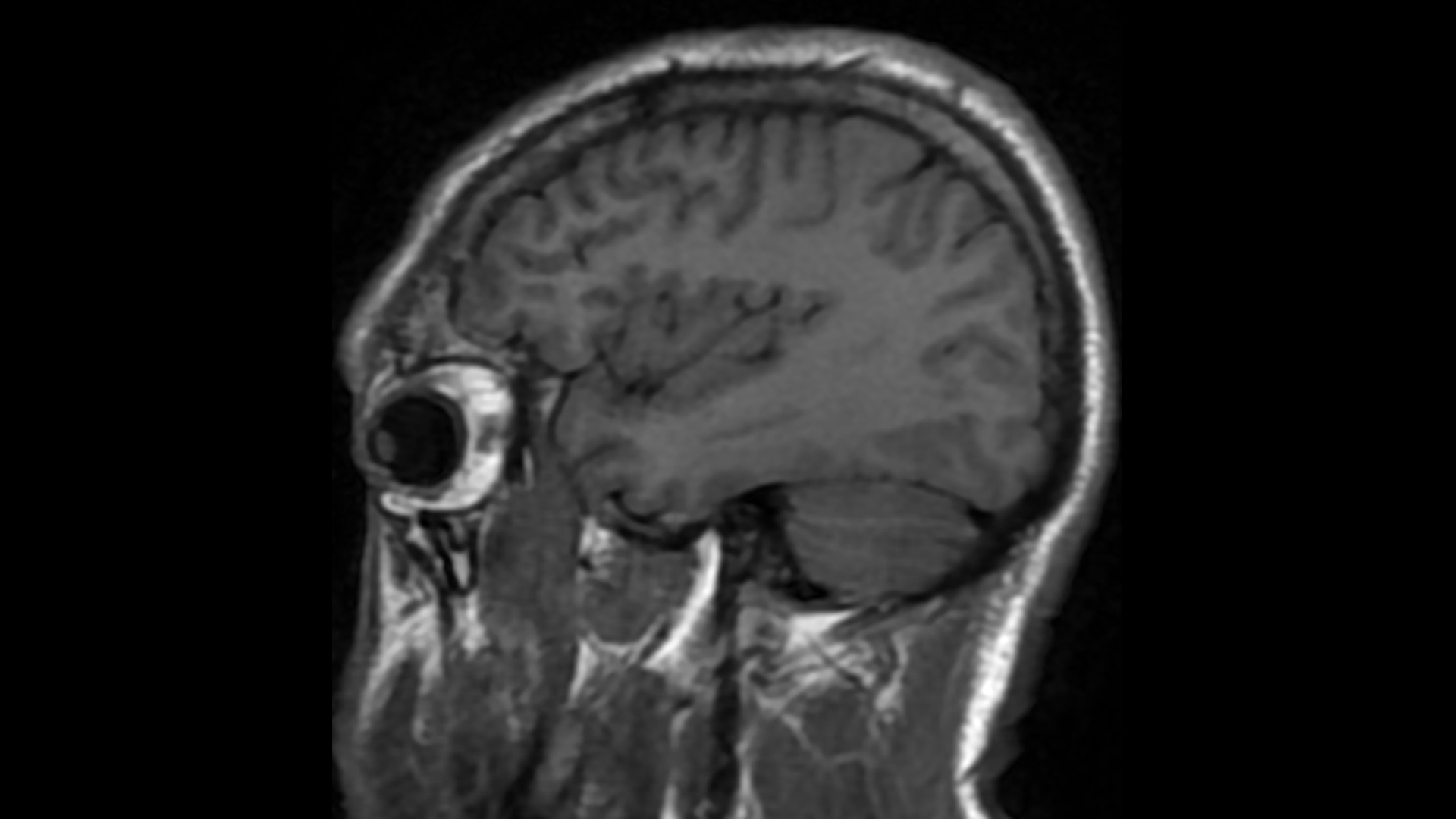

Supplement: Supplementary file 1 [file jemr-19-00062-s001.zip › jemr-4253847-supplementary/Supplementary files/1_StimuliImages/Brain_stimuli/BRAIN_22_NORMAL_Normal Insula.png]

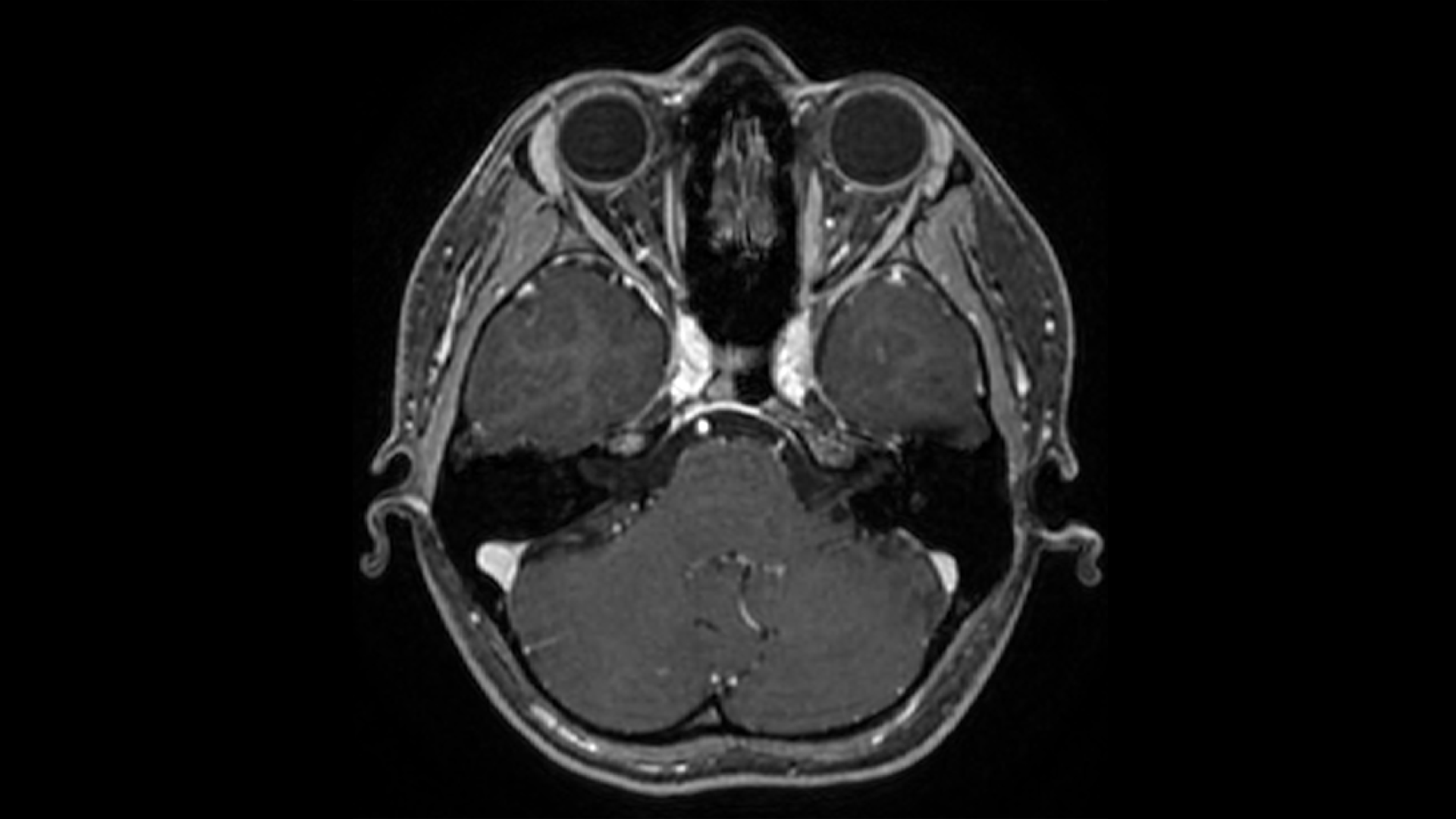

Supplement: Supplementary file 1 [file jemr-19-00062-s001.zip › jemr-4253847-supplementary/Supplementary files/1_StimuliImages/Brain_stimuli/BRAIN_23_NORMAL_norm T1+.png]

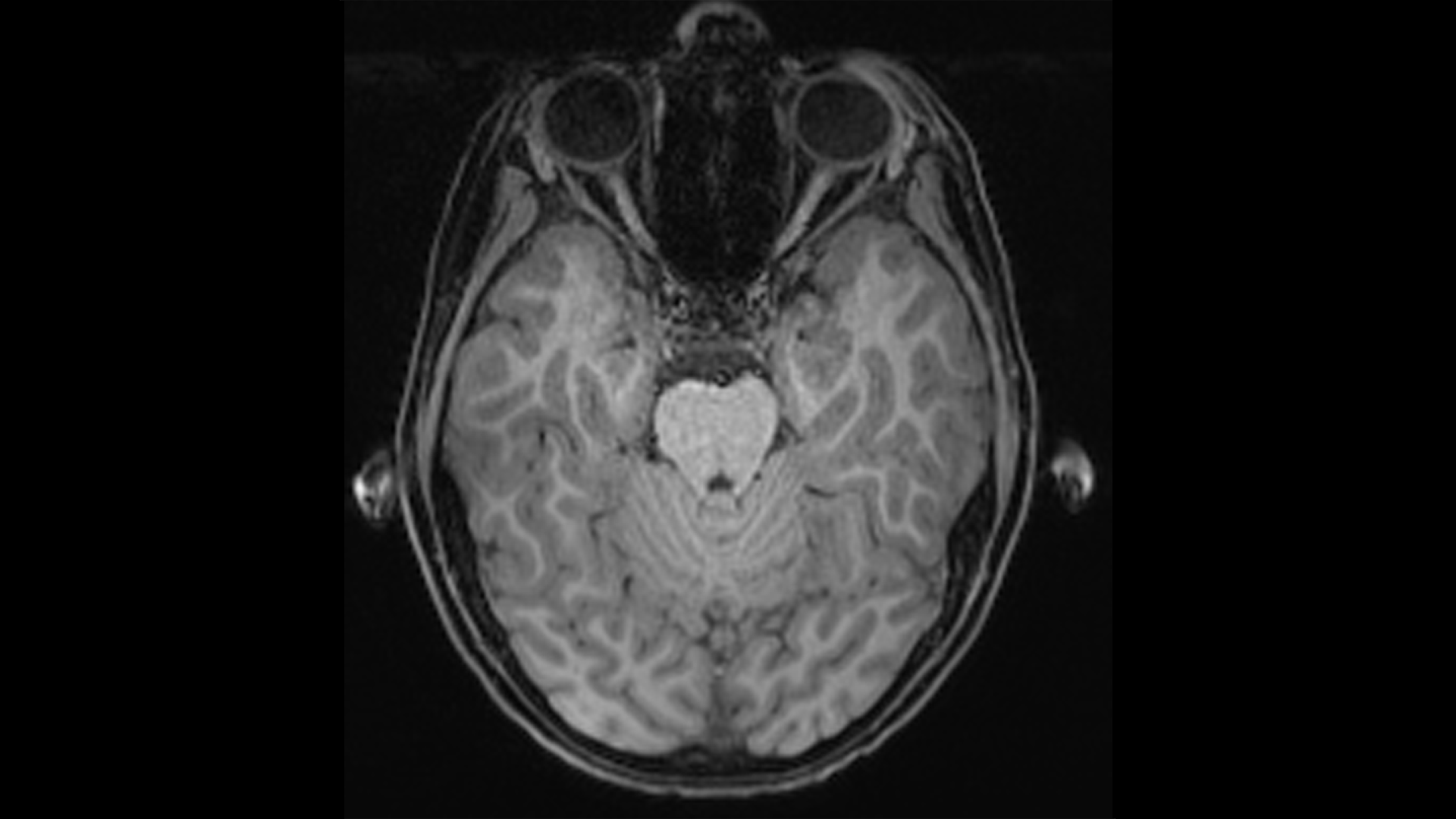

Supplement: Supplementary file 1 [file jemr-19-00062-s001.zip › jemr-4253847-supplementary/Supplementary files/1_StimuliImages/Brain_stimuli/BRAIN_24_NORMAL_Ax.png]

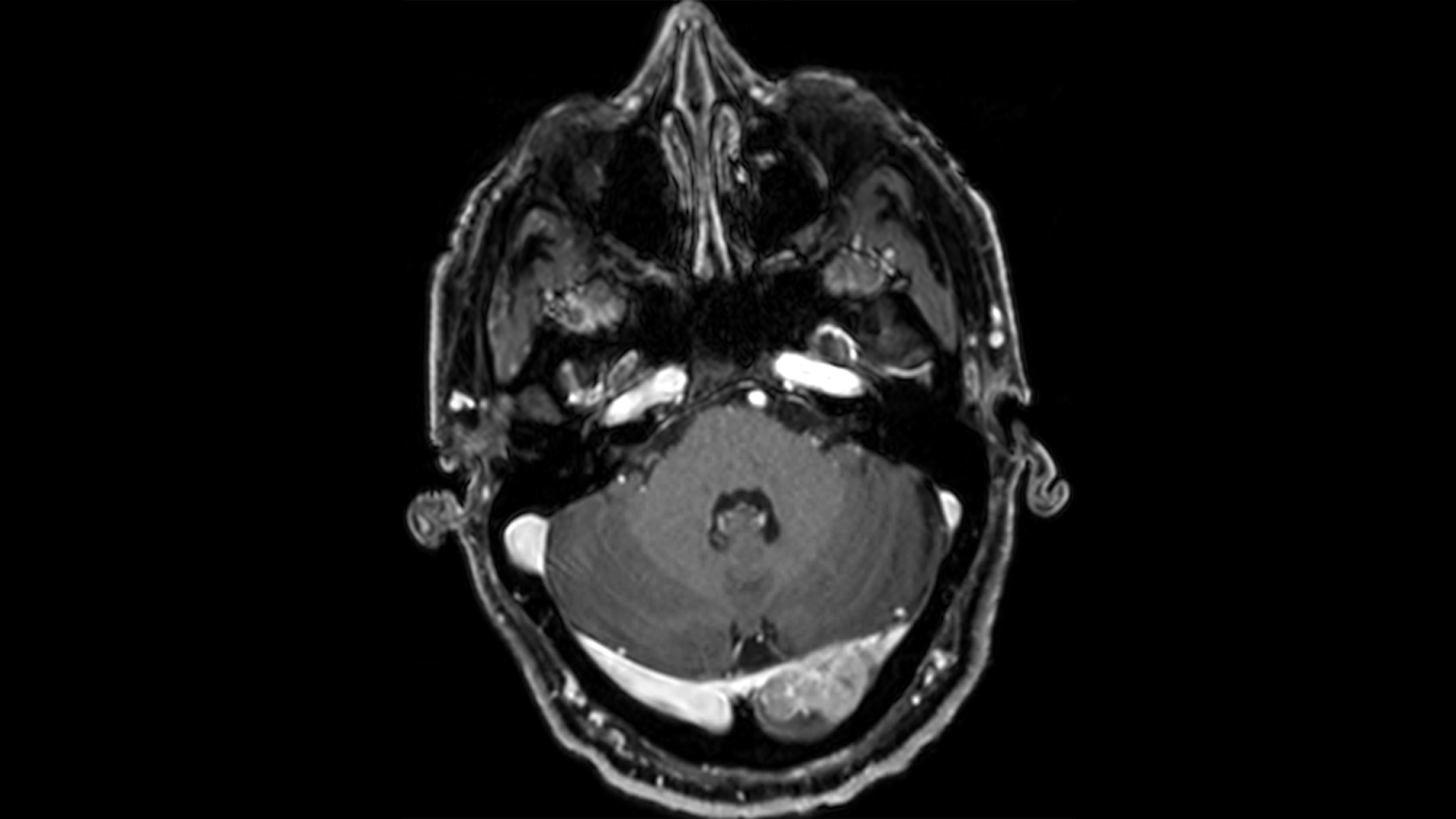

Supplement: Supplementary file 1 [file jemr-19-00062-s001.zip › jemr-4253847-supplementary/Supplementary files/1_StimuliImages/Brain_stimuli/BRAIN_25_PATH_Transv sinus meningioma.png]

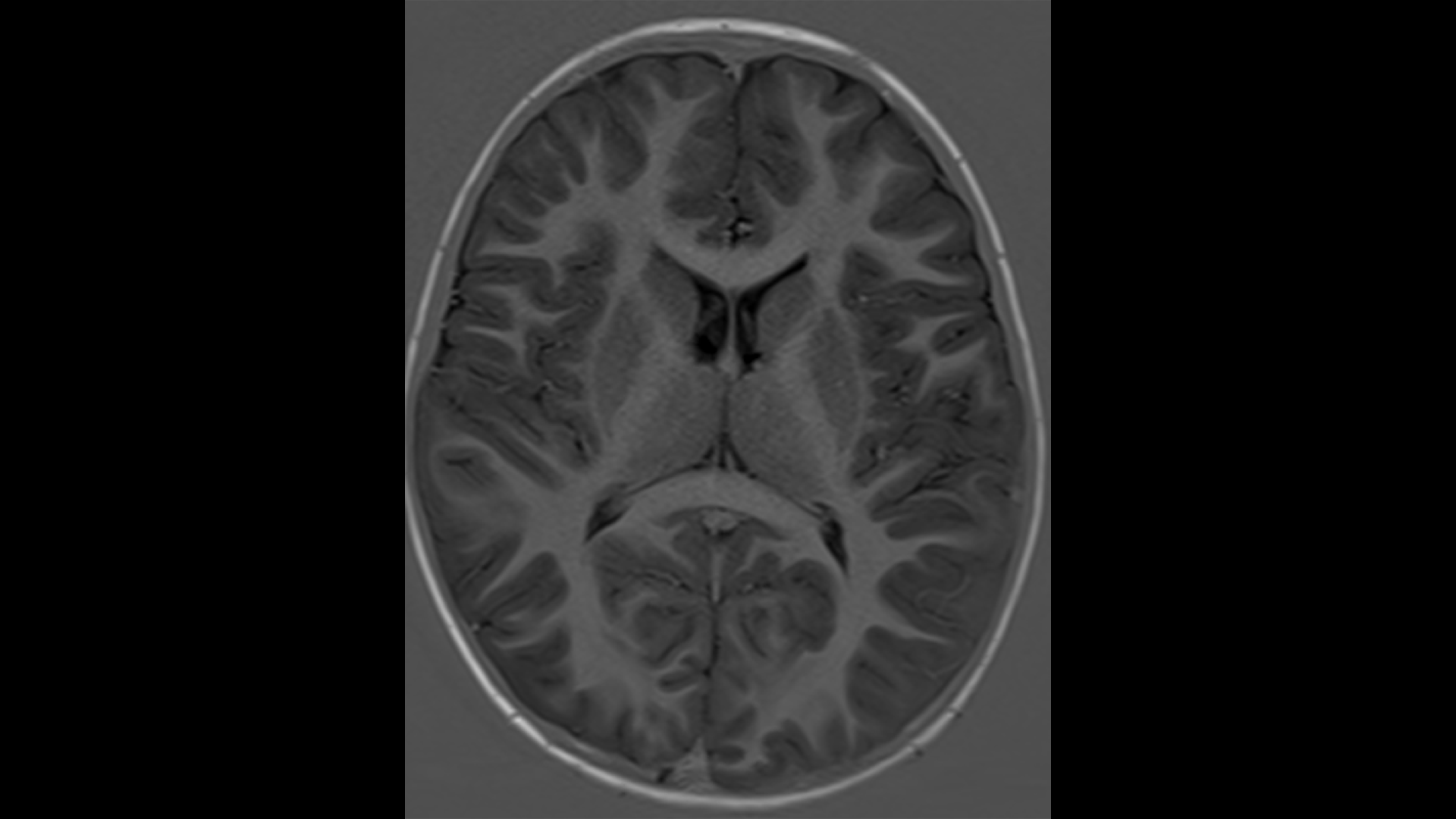

Supplement: Supplementary file 1 [file jemr-19-00062-s001.zip › jemr-4253847-supplementary/Supplementary files/1_StimuliImages/Brain_stimuli/BRAIN_26_NORMAL_BG.png]

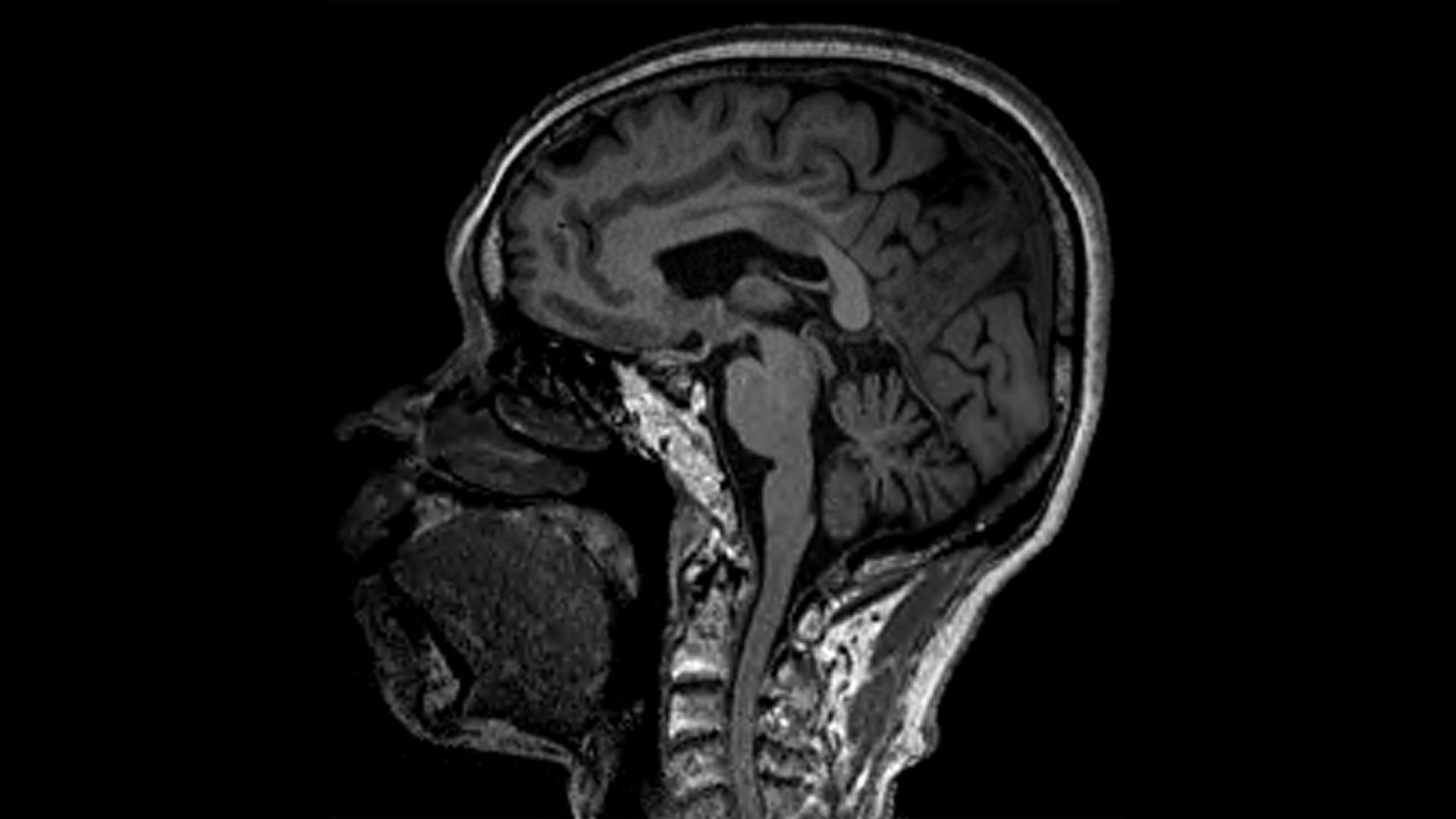

Supplement: Supplementary file 1 [file jemr-19-00062-s001.zip › jemr-4253847-supplementary/Supplementary files/1_StimuliImages/Brain_stimuli/BRAIN_27_PATH_Sagittal GBM.png]

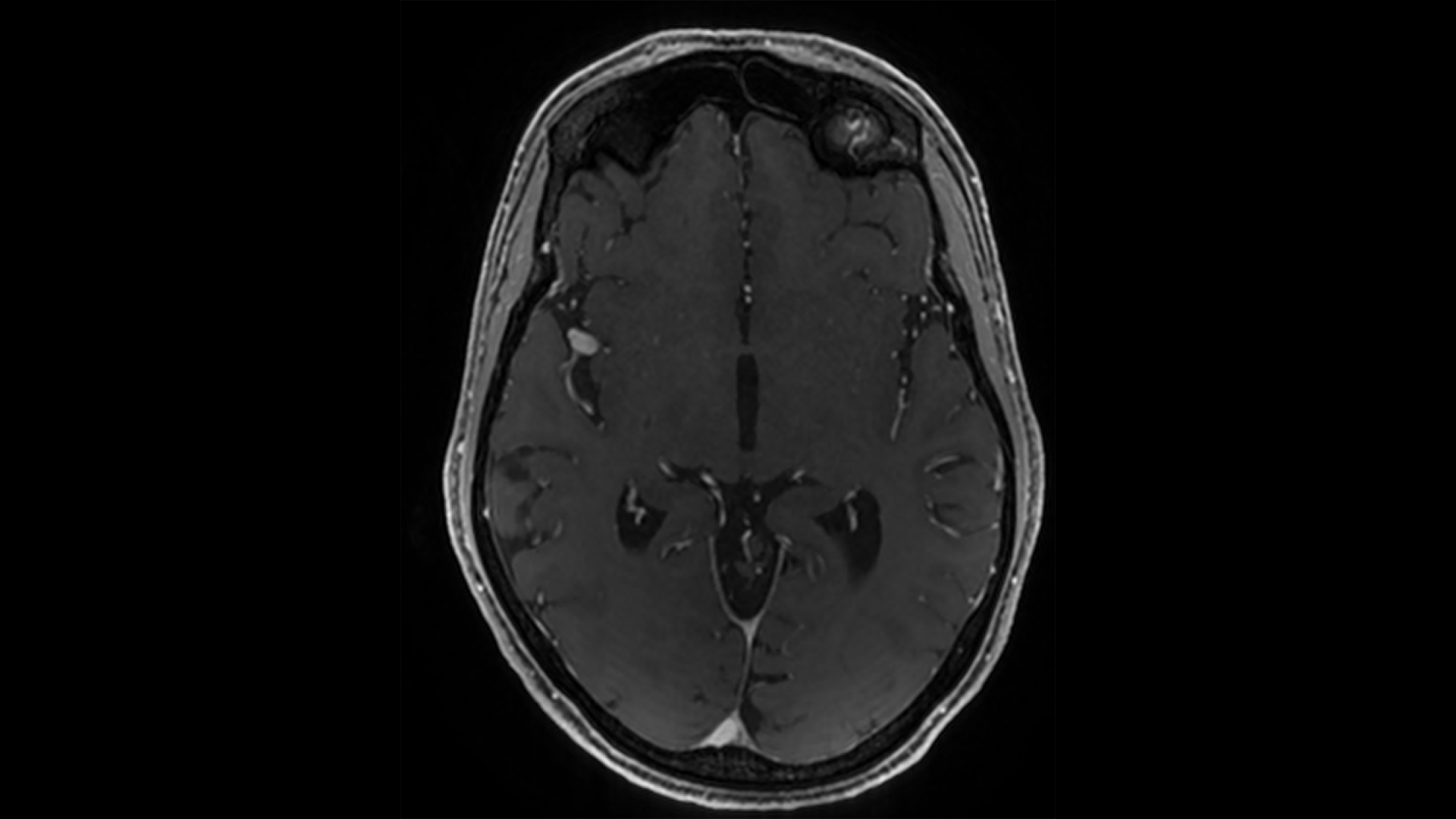

Supplement: Supplementary file 1 [file jemr-19-00062-s001.zip › jemr-4253847-supplementary/Supplementary files/1_StimuliImages/Brain_stimuli/BRAIN_28_PATH_Aneurysm.png]

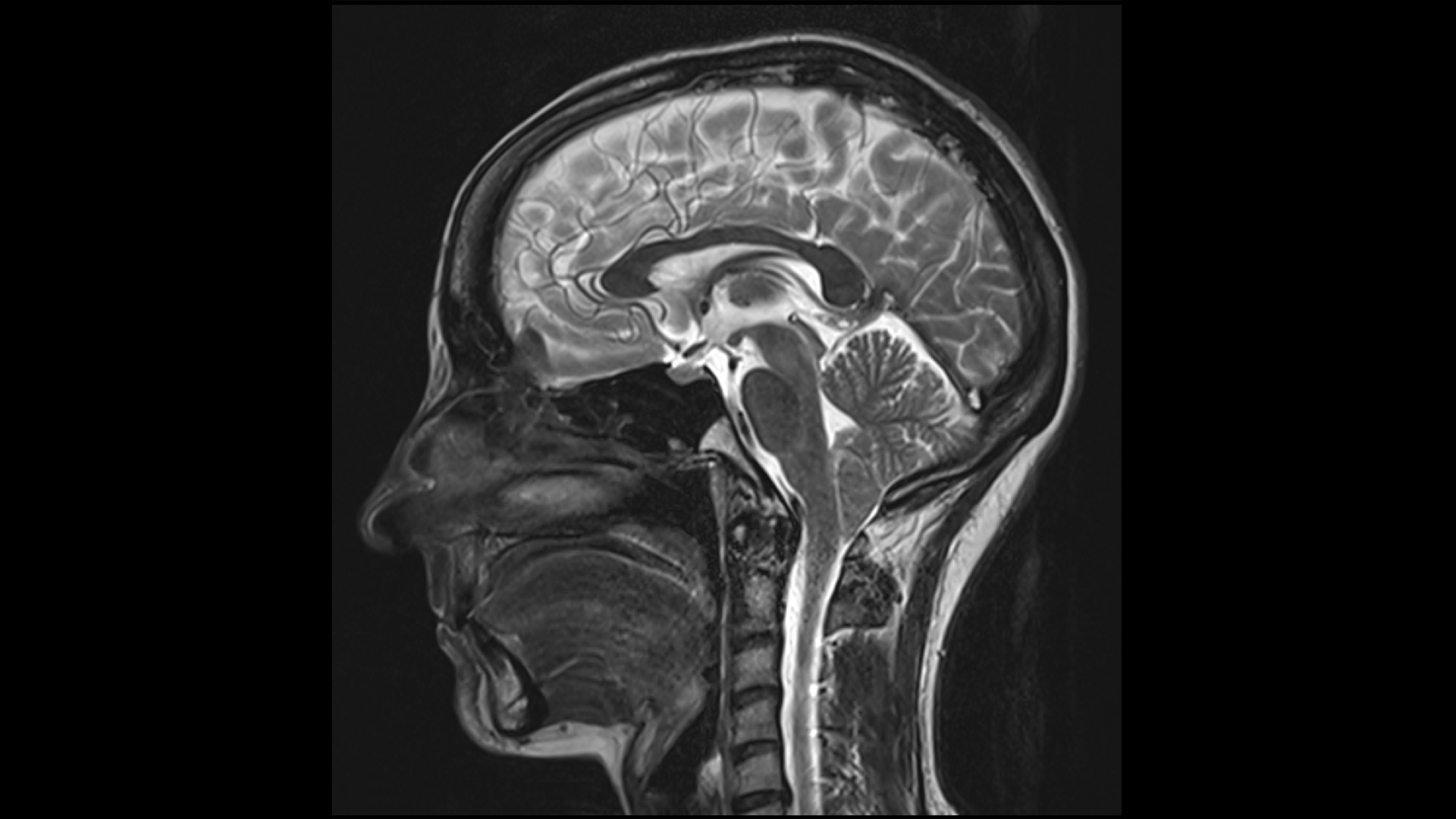

Supplement: Supplementary file 1 [file jemr-19-00062-s001.zip › jemr-4253847-supplementary/Supplementary files/1_StimuliImages/Brain_stimuli/BRAIN_29_PATH_Chiari.png]

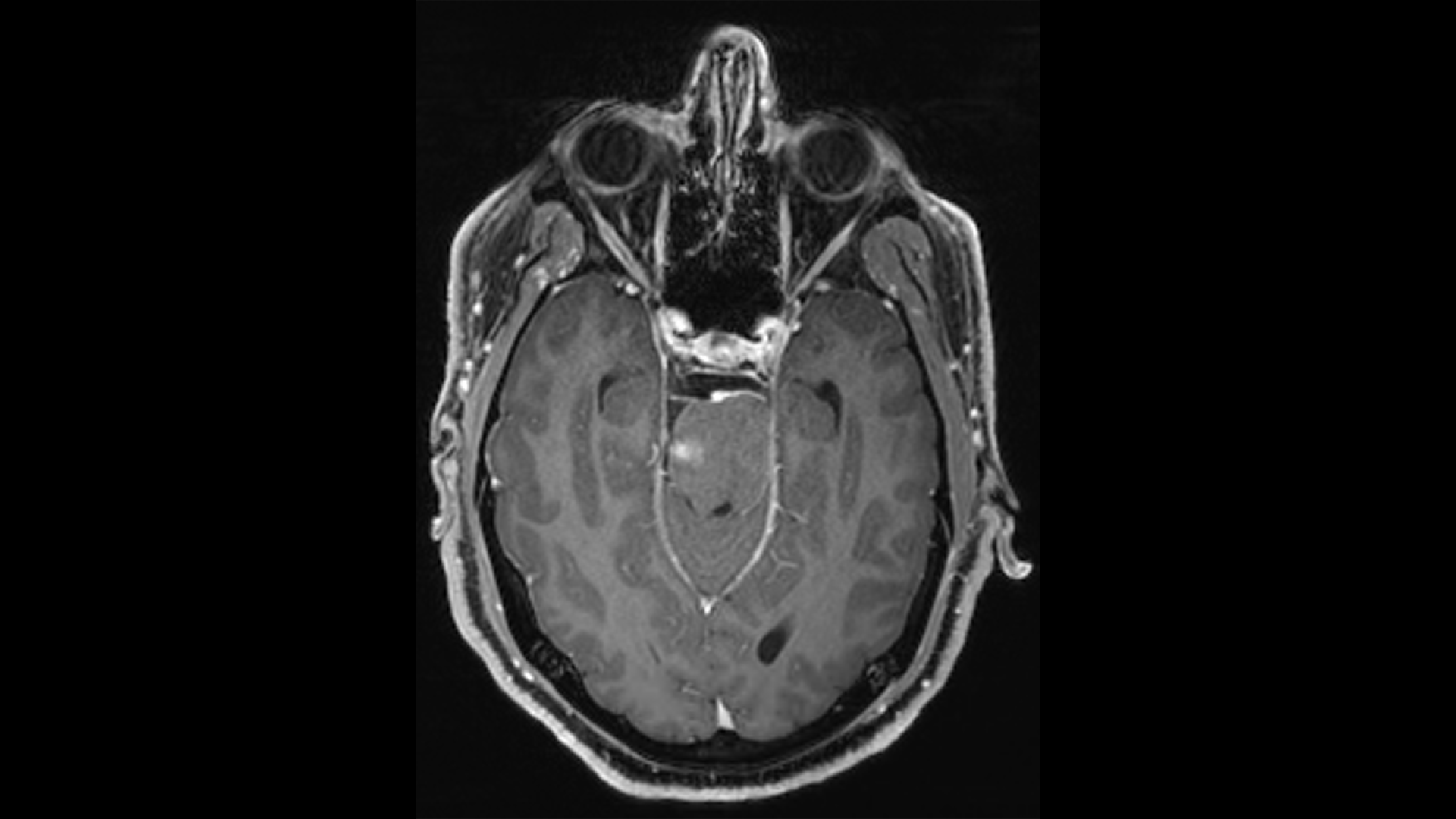

Supplement: Supplementary file 1 [file jemr-19-00062-s001.zip › jemr-4253847-supplementary/Supplementary files/1_StimuliImages/Brain_stimuli/BRAIN_2_PATH_Mes tumour.png]

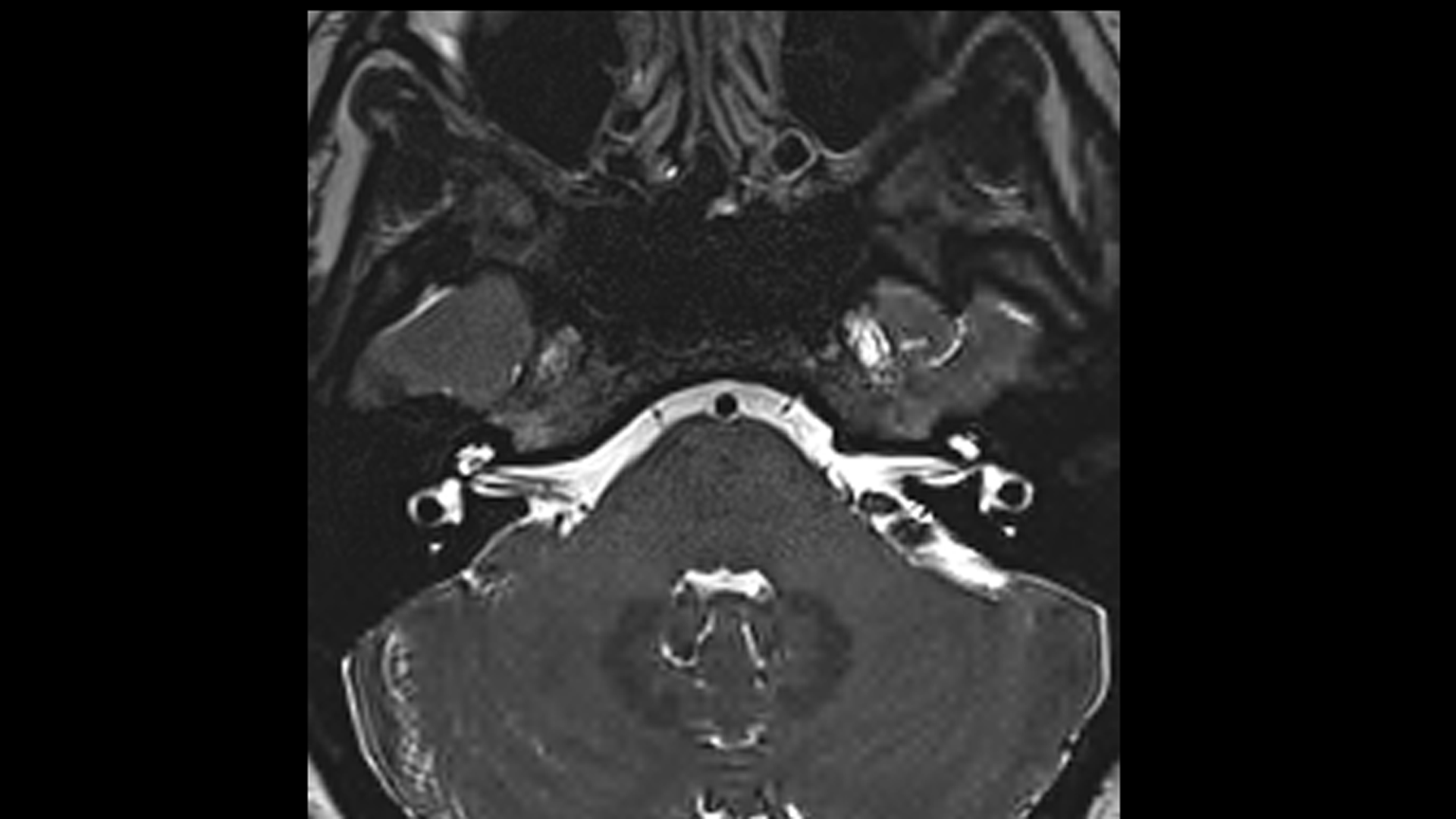

Supplement: Supplementary file 1 [file jemr-19-00062-s001.zip › jemr-4253847-supplementary/Supplementary files/1_StimuliImages/Brain_stimuli/BRAIN_30_NORMAL_Normal IAM.png]

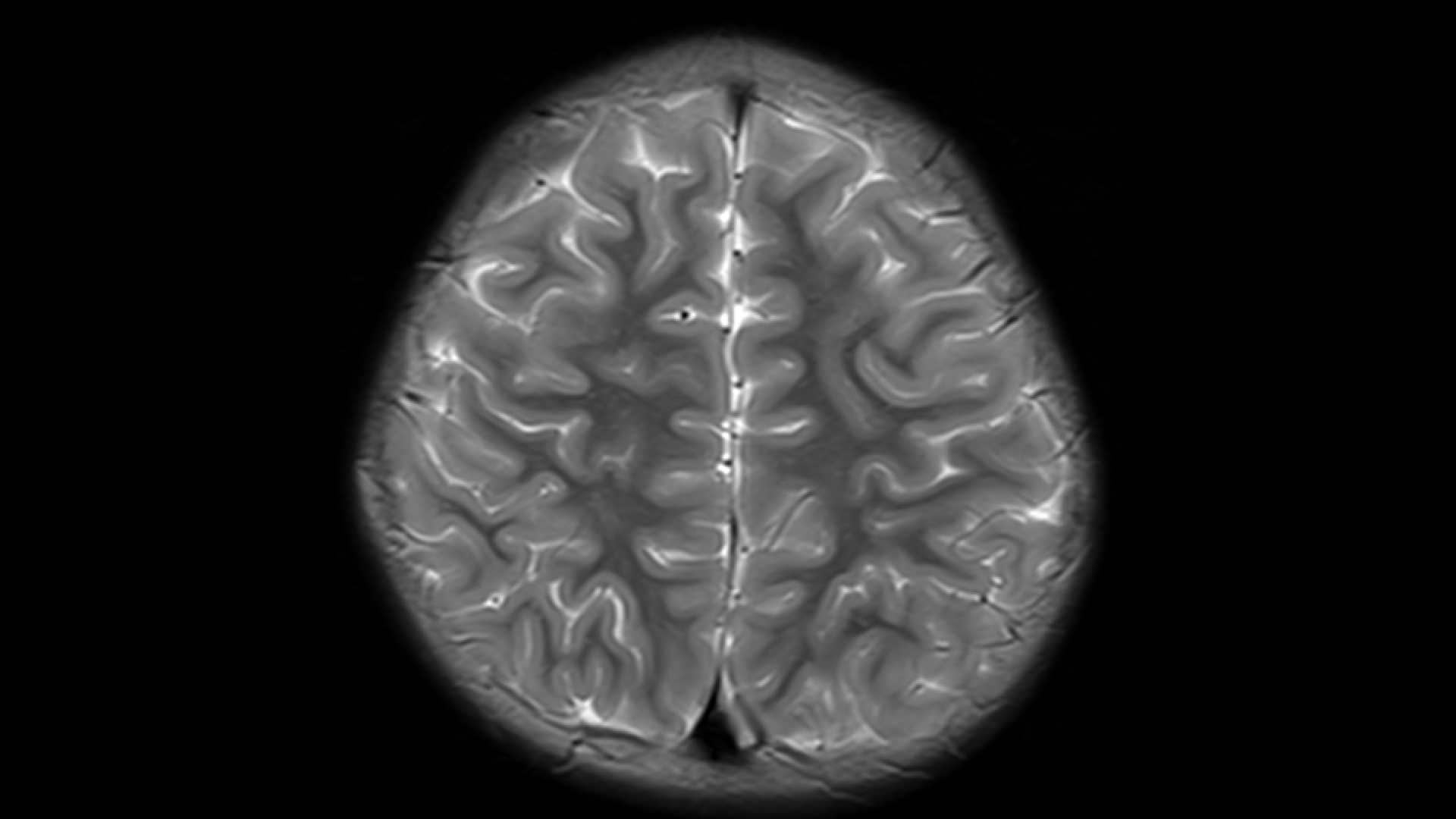

Supplement: Supplementary file 1 [file jemr-19-00062-s001.zip › jemr-4253847-supplementary/Supplementary files/1_StimuliImages/Brain_stimuli/BRAIN_31_NORMAL_Sulci.png]

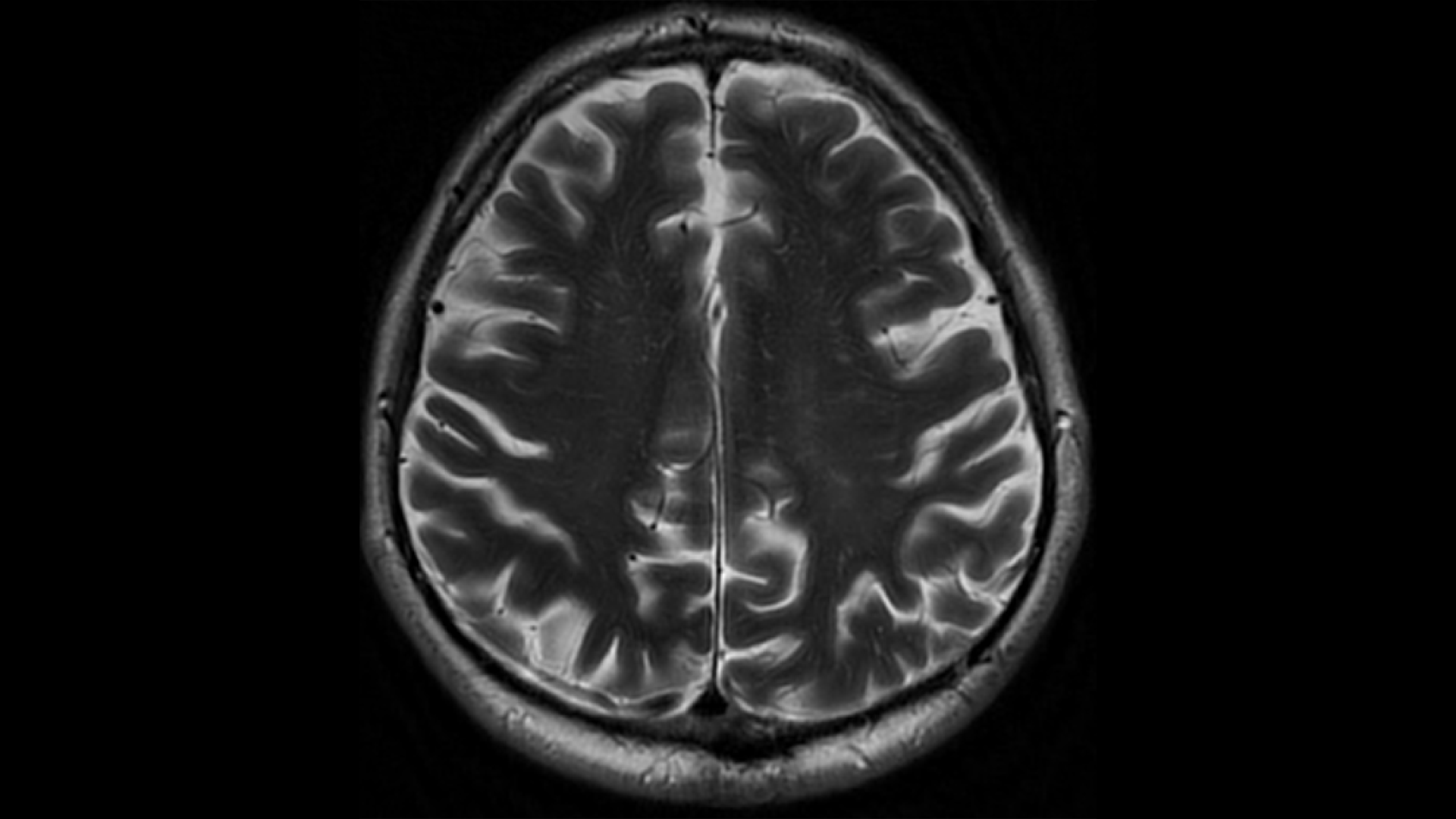

Supplement: Supplementary file 1 [file jemr-19-00062-s001.zip › jemr-4253847-supplementary/Supplementary files/1_StimuliImages/Brain_stimuli/BRAIN_32_NORMAL_Axial T2.png]

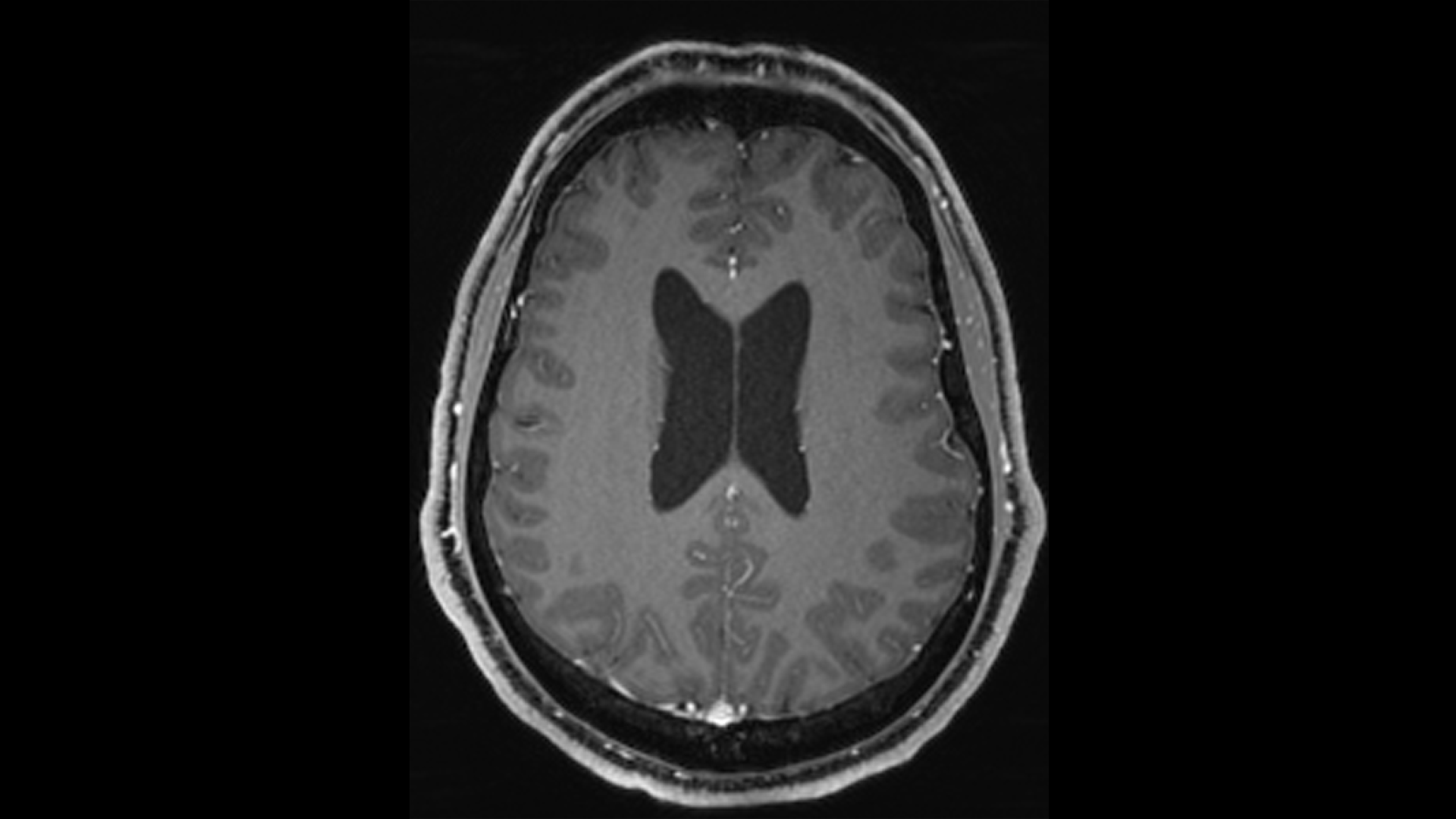

Supplement: Supplementary file 1 [file jemr-19-00062-s001.zip › jemr-4253847-supplementary/Supplementary files/1_StimuliImages/Brain_stimuli/BRAIN_33_NORMAL_T1C ventricles.png]

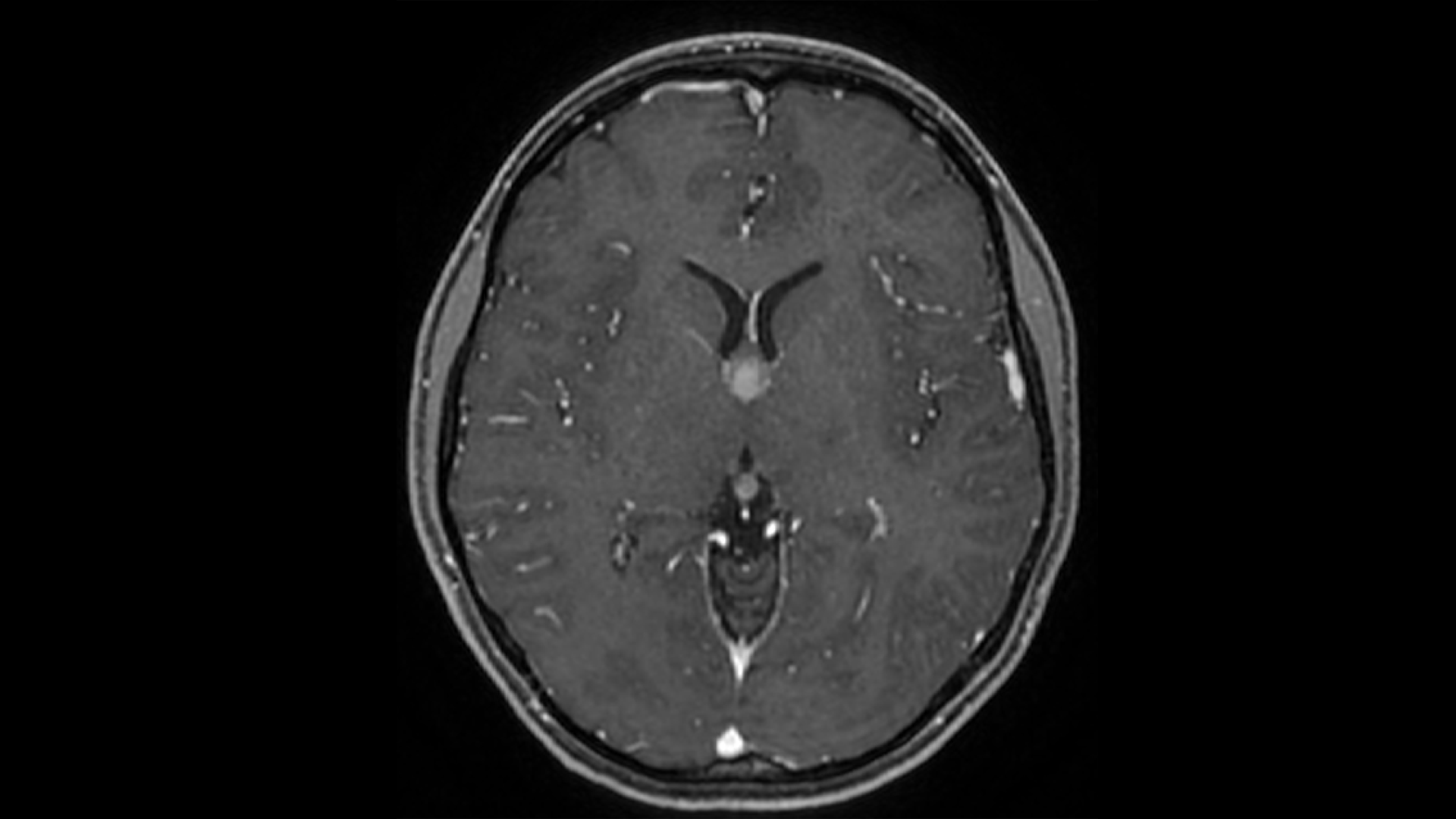

Supplement: Supplementary file 1 [file jemr-19-00062-s001.zip › jemr-4253847-supplementary/Supplementary files/1_StimuliImages/Brain_stimuli/BRAIN_34_PATH_Colloid and pineal cysts.png]

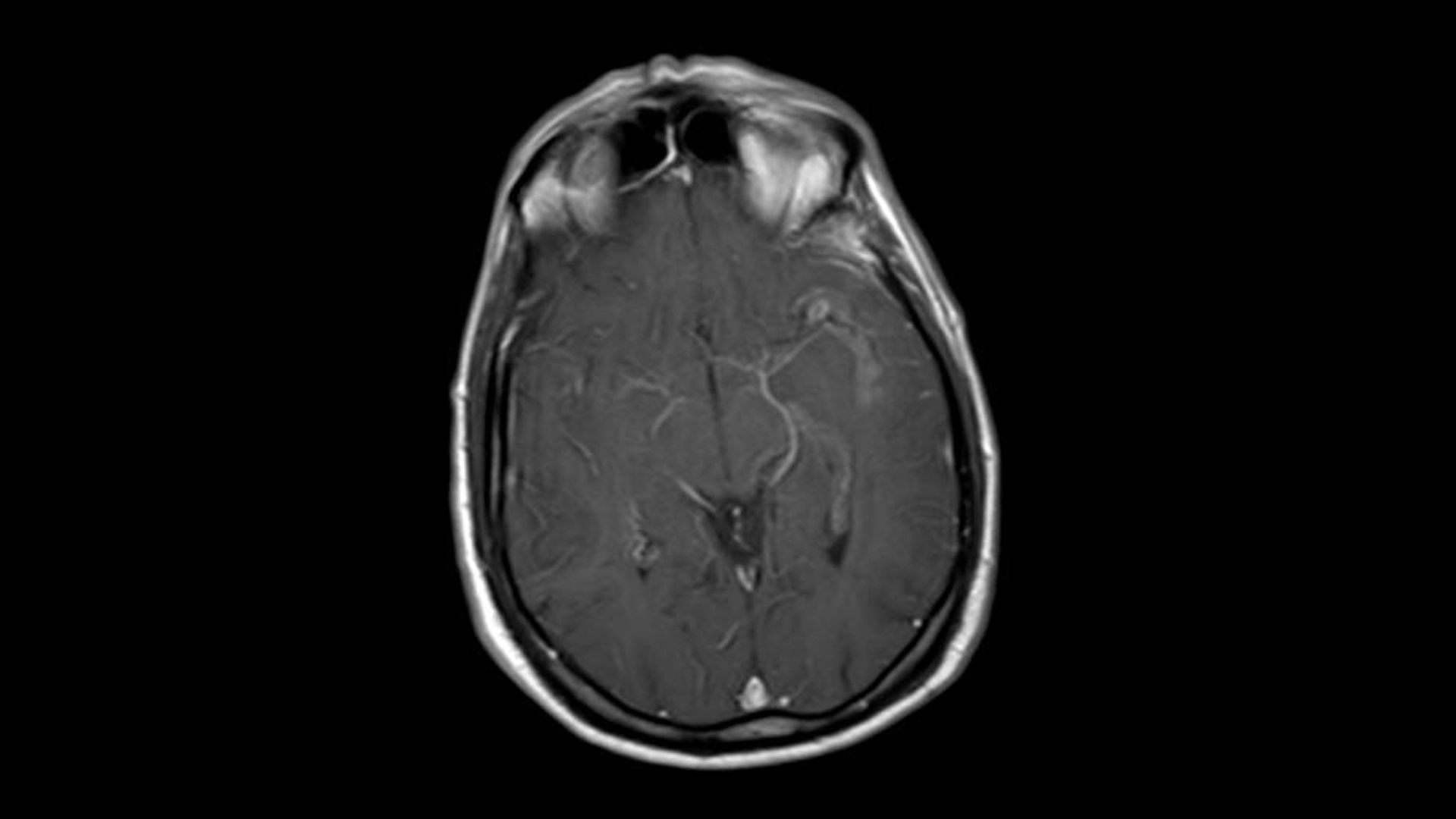

Supplement: Supplementary file 1 [file jemr-19-00062-s001.zip › jemr-4253847-supplementary/Supplementary files/1_StimuliImages/Brain_stimuli/BRAIN_35_PATH_SAH.png]

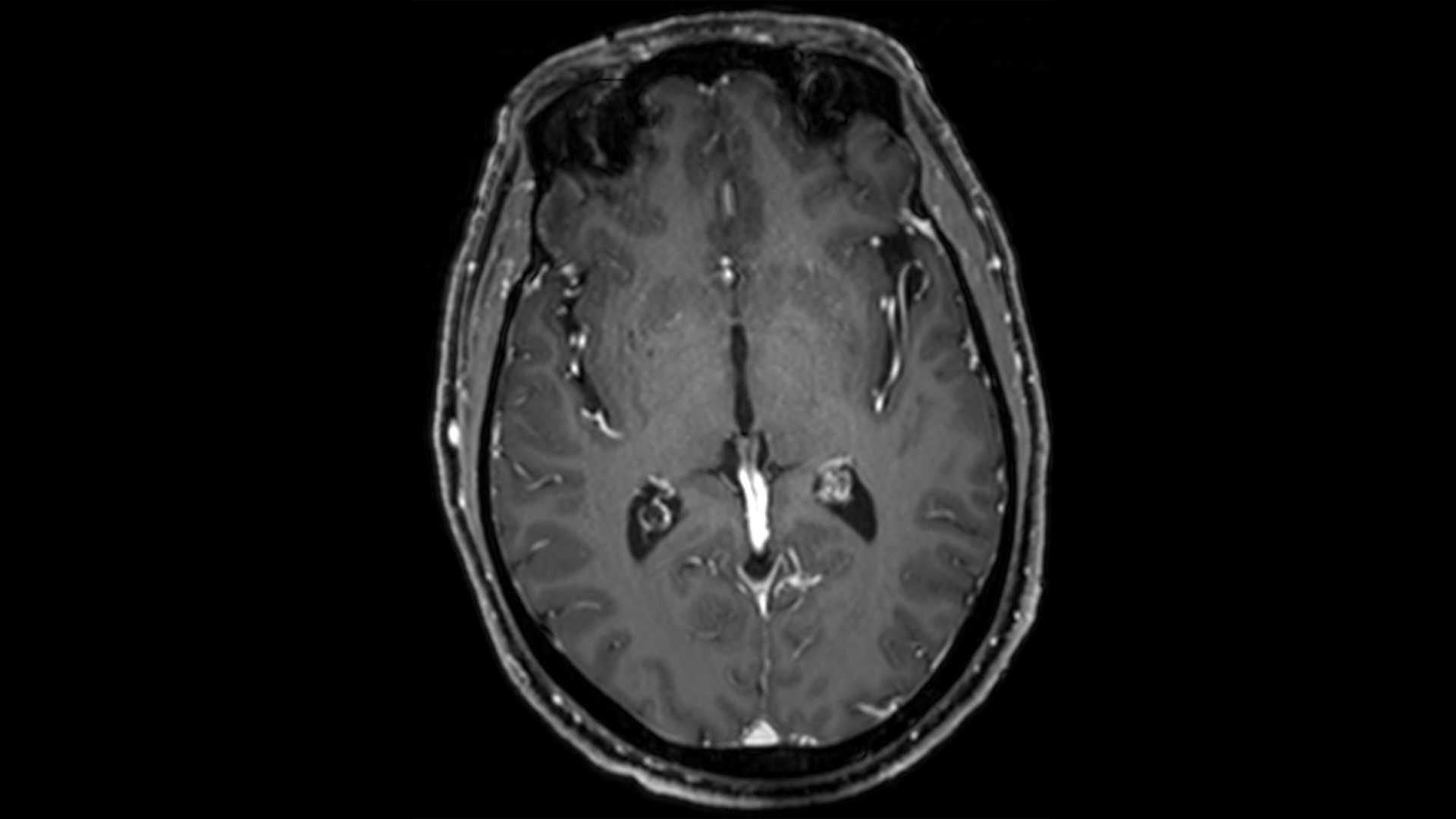

Supplement: Supplementary file 1 [file jemr-19-00062-s001.zip › jemr-4253847-supplementary/Supplementary files/1_StimuliImages/Brain_stimuli/BRAIN_36_NORMAL_T1C a.png]

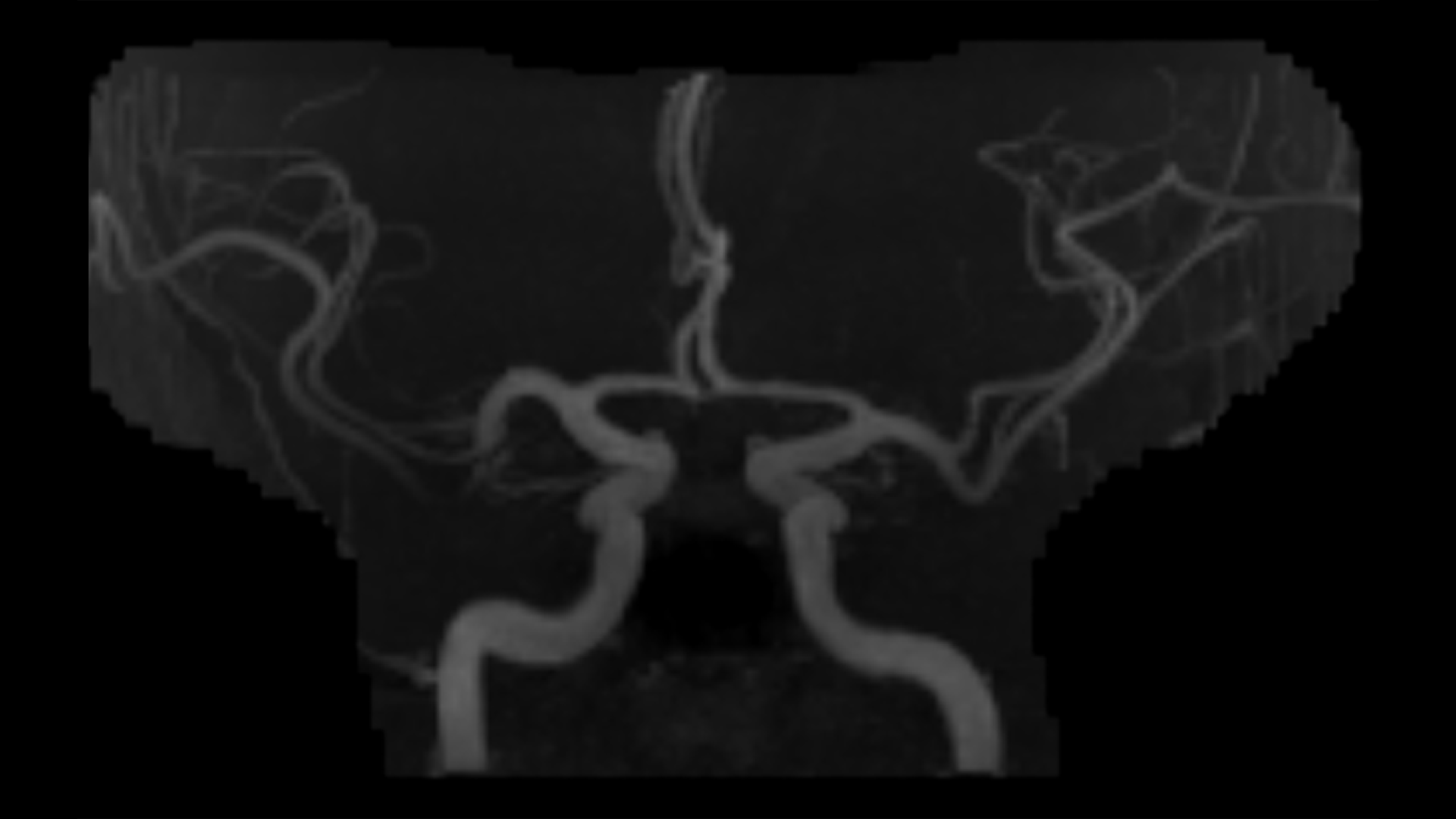

Supplement: Supplementary file 1 [file jemr-19-00062-s001.zip › jemr-4253847-supplementary/Supplementary files/1_StimuliImages/Brain_stimuli/BRAIN_37_NORMAL_Normal MRA.png]

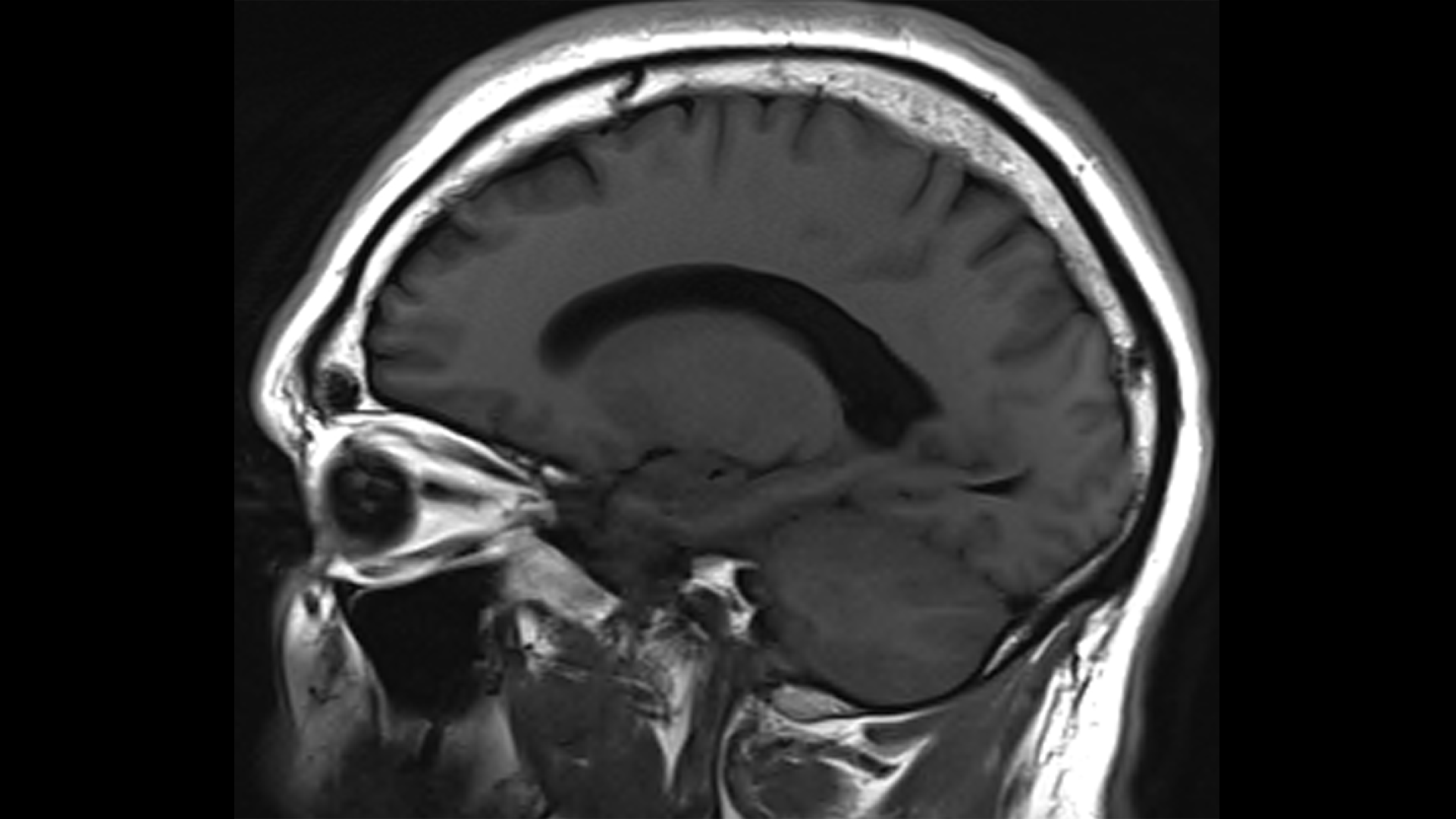

Supplement: Supplementary file 1 [file jemr-19-00062-s001.zip › jemr-4253847-supplementary/Supplementary files/1_StimuliImages/Brain_stimuli/BRAIN_38_NORMAL_Sagittal T1.png]

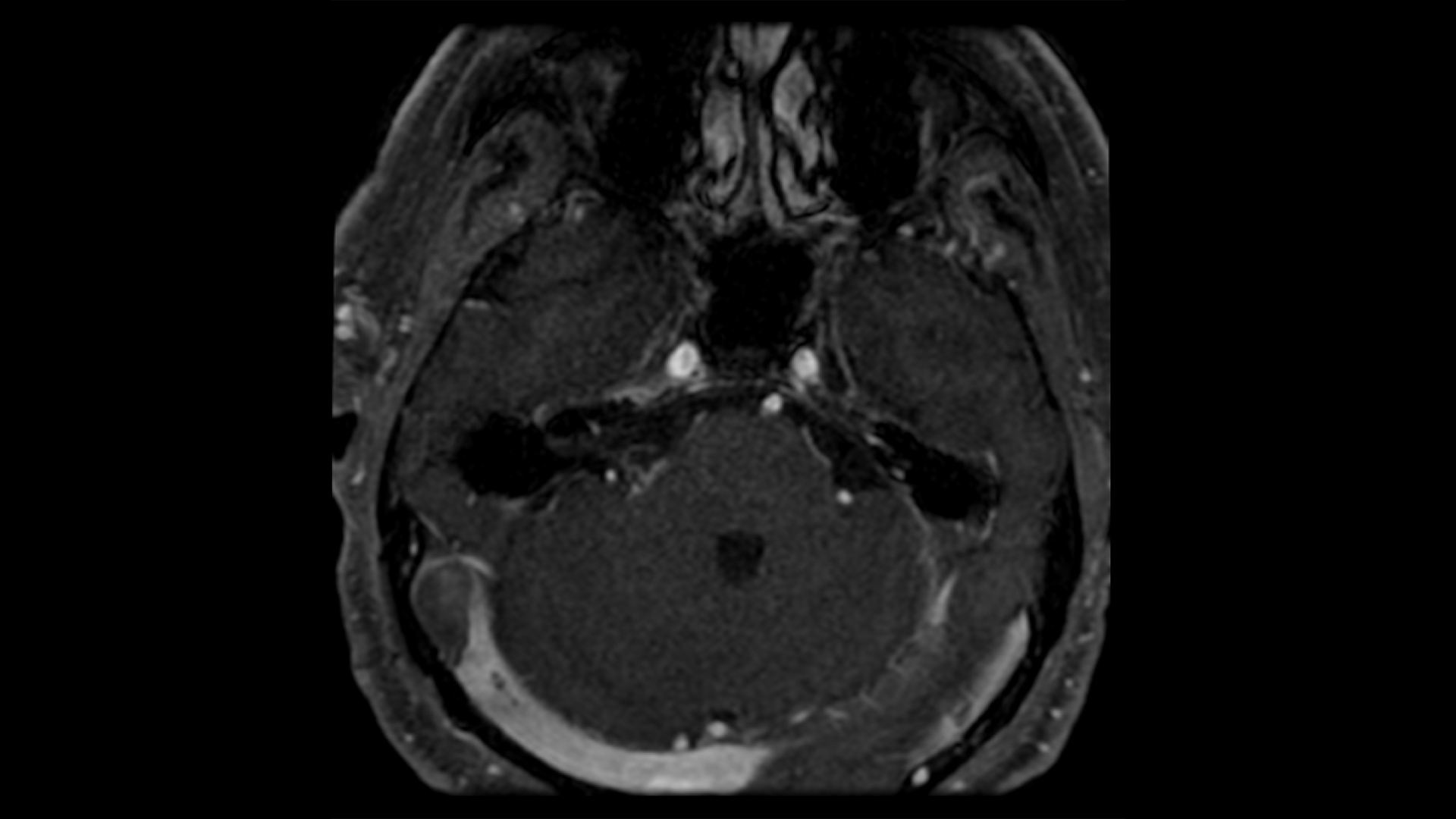

Supplement: Supplementary file 1 [file jemr-19-00062-s001.zip › jemr-4253847-supplementary/Supplementary files/1_StimuliImages/Brain_stimuli/BRAIN_39_PATH_Thrombosis.png]

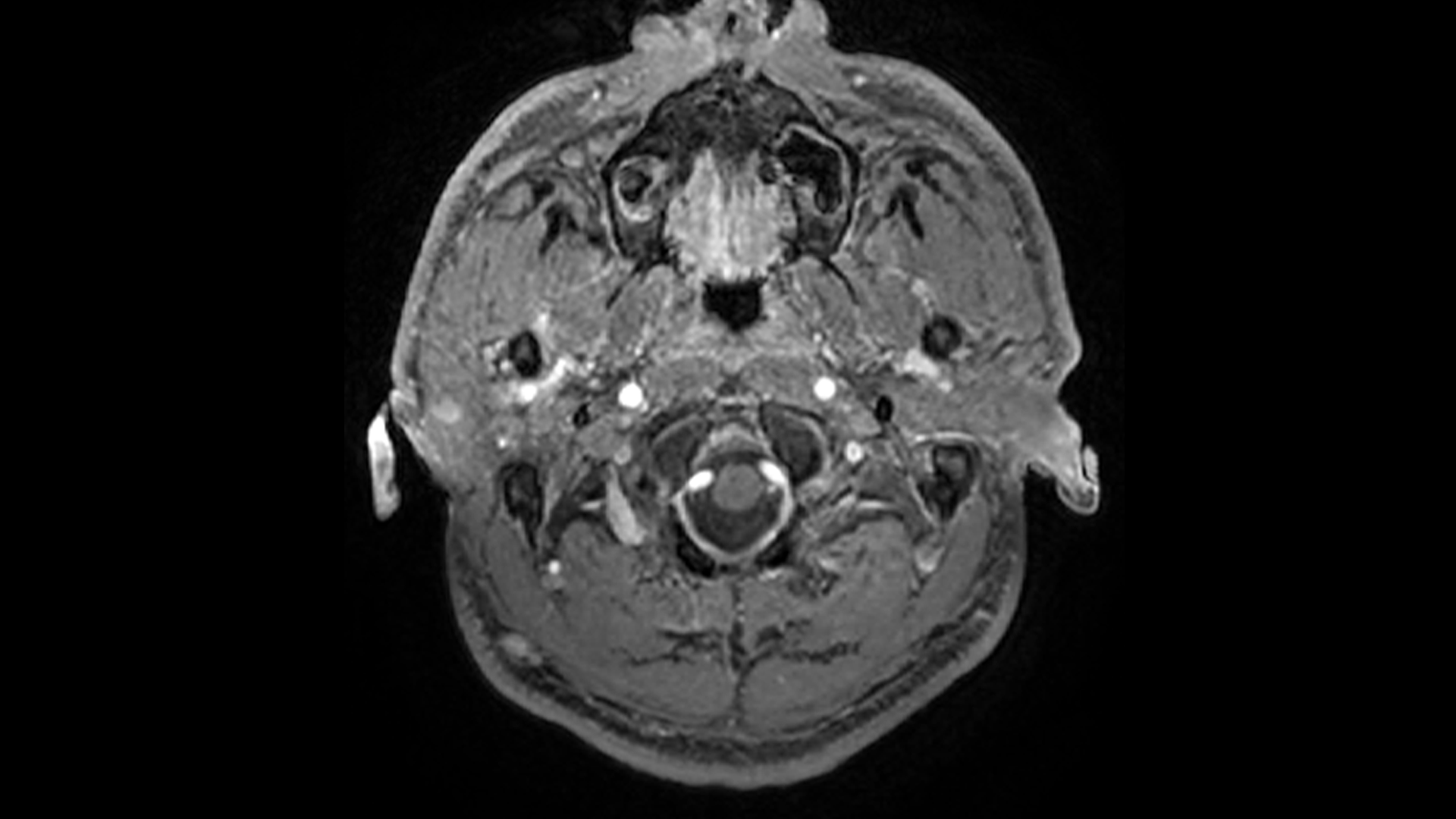

Supplement: Supplementary file 1 [file jemr-19-00062-s001.zip › jemr-4253847-supplementary/Supplementary files/1_StimuliImages/Brain_stimuli/BRAIN_3_NORMAL_Normal 4 vessels.png]

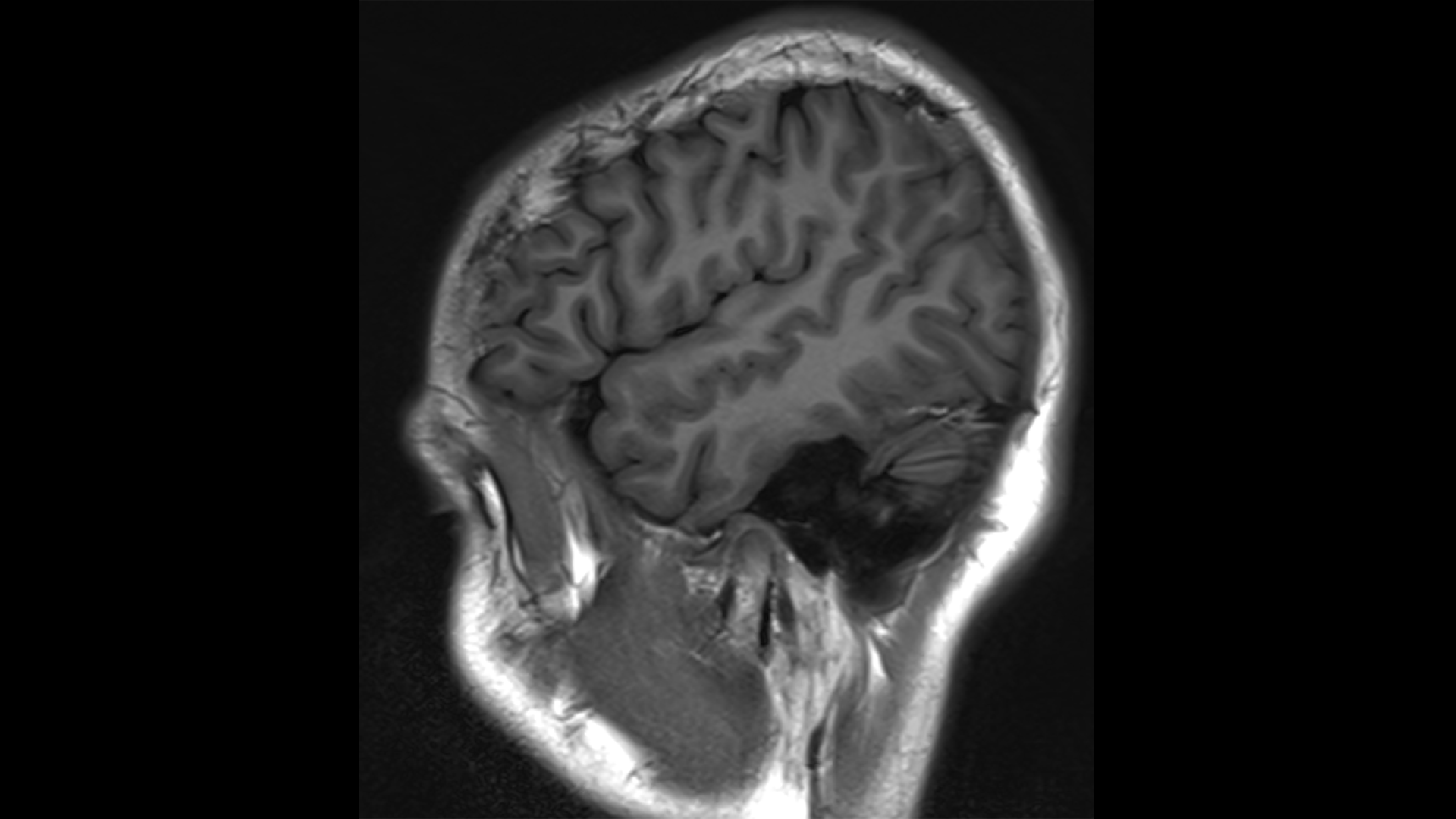

Supplement: Supplementary file 1 [file jemr-19-00062-s001.zip › jemr-4253847-supplementary/Supplementary files/1_StimuliImages/Brain_stimuli/BRAIN_40_NORMAL_Sylvian.png]

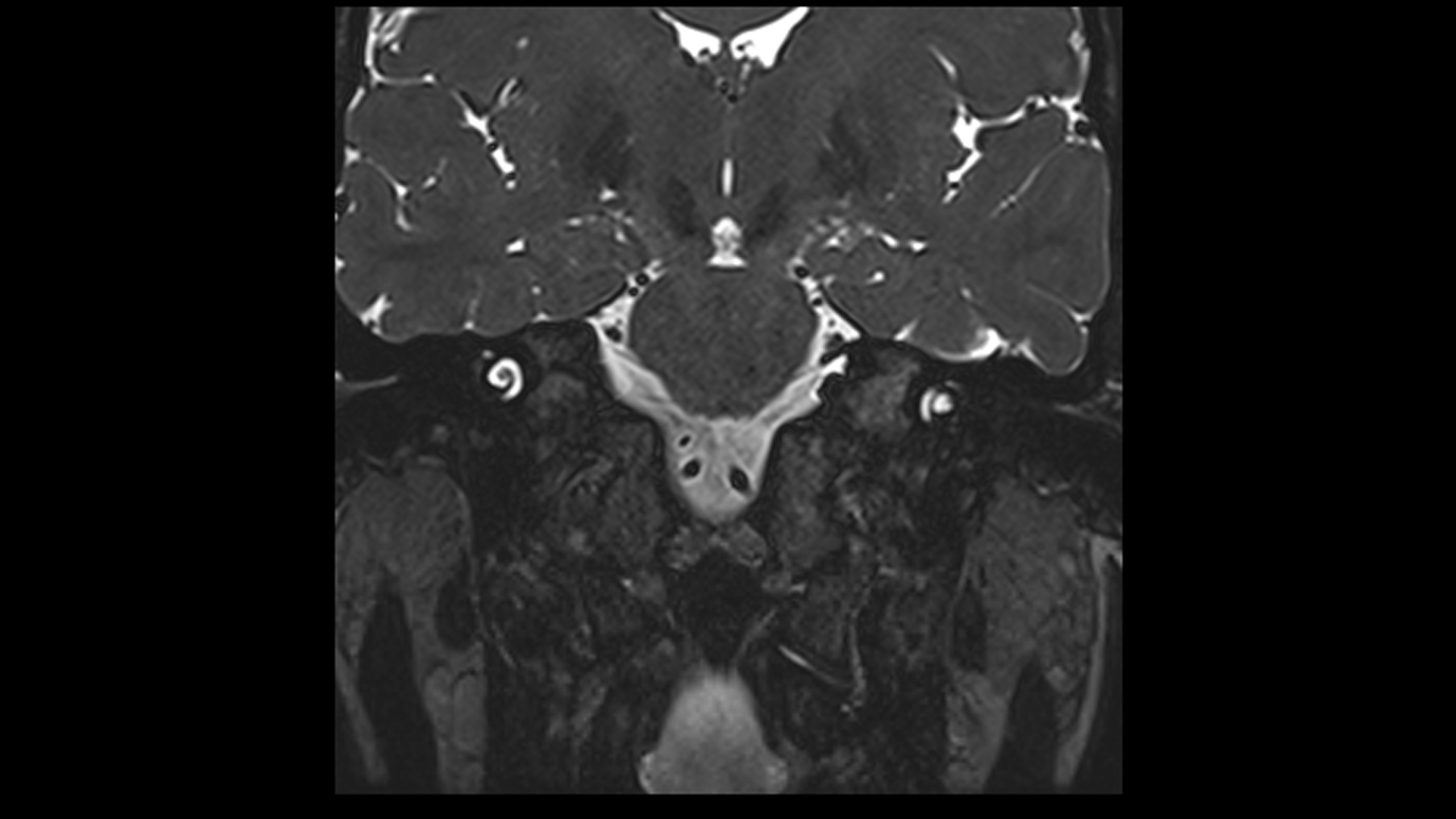

Supplement: Supplementary file 1 [file jemr-19-00062-s001.zip › jemr-4253847-supplementary/Supplementary files/1_StimuliImages/Brain_stimuli/BRAIN_4_NORMAL_Normal Coronal T2.png]

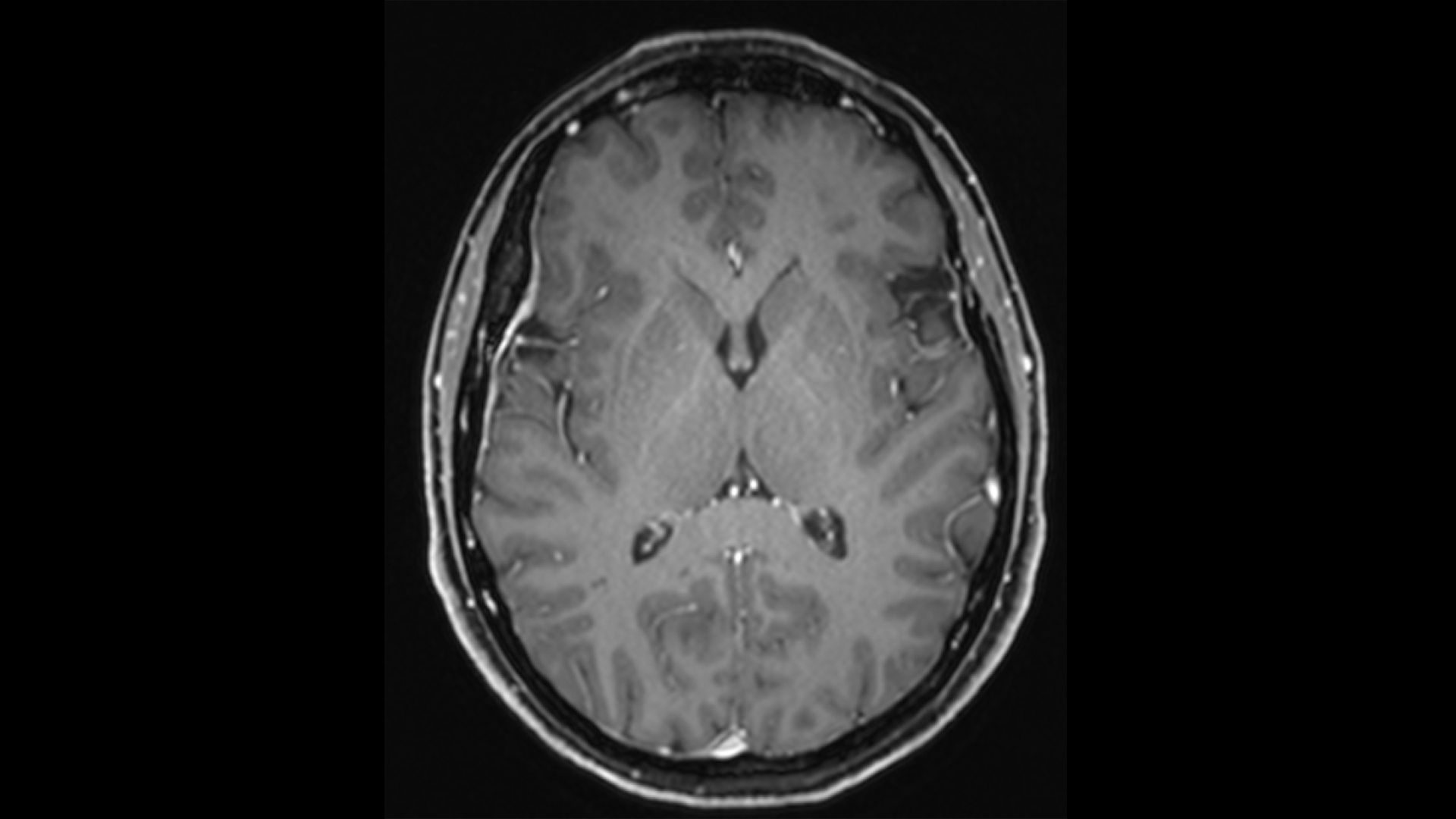

Supplement: Supplementary file 1 [file jemr-19-00062-s001.zip › jemr-4253847-supplementary/Supplementary files/1_StimuliImages/Brain_stimuli/BRAIN_5_PATH_Dural ring.png]

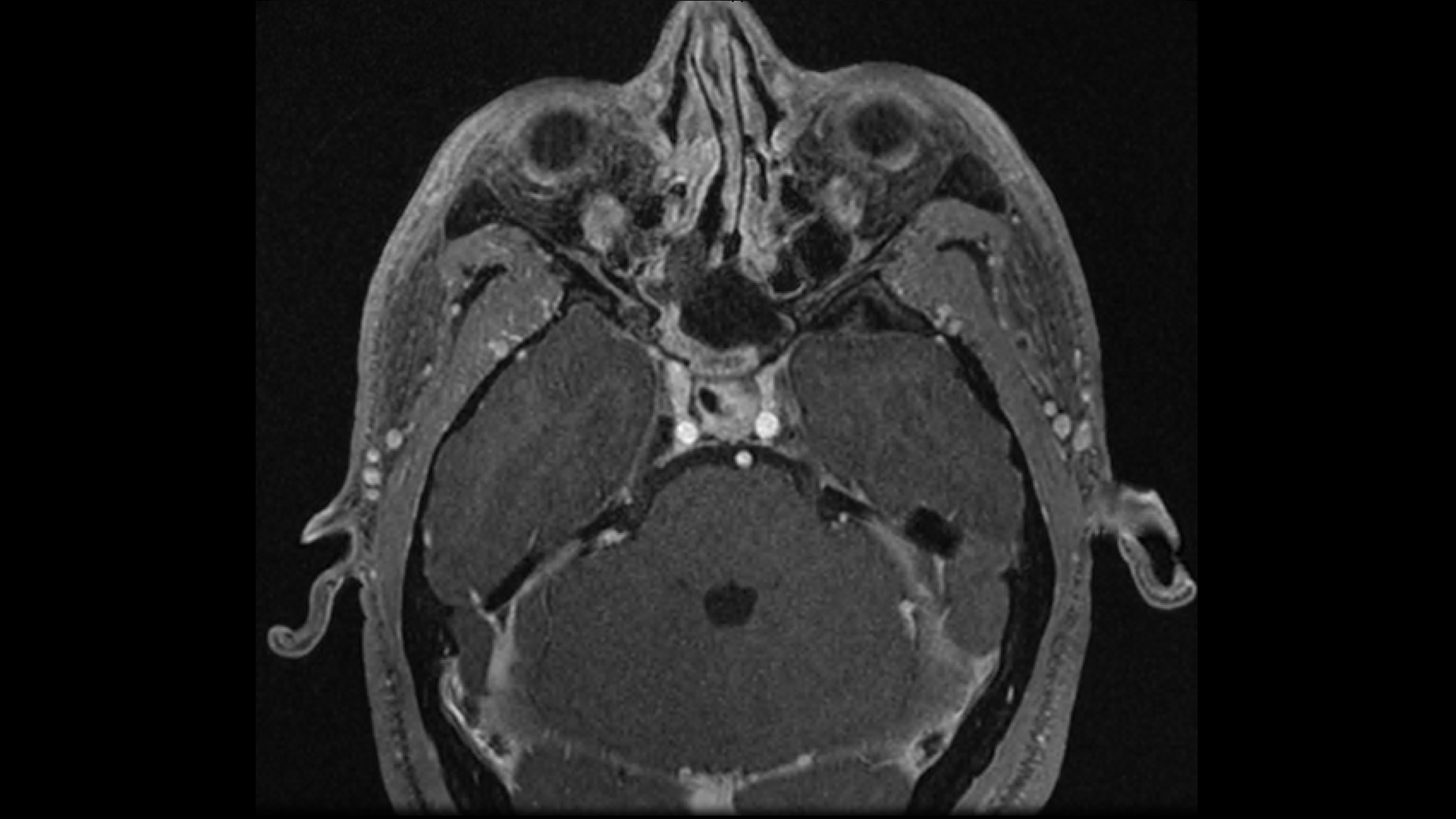

Supplement: Supplementary file 1 [file jemr-19-00062-s001.zip › jemr-4253847-supplementary/Supplementary files/1_StimuliImages/Brain_stimuli/BRAIN_6_PATH_Post surgical pit adenoma.png]

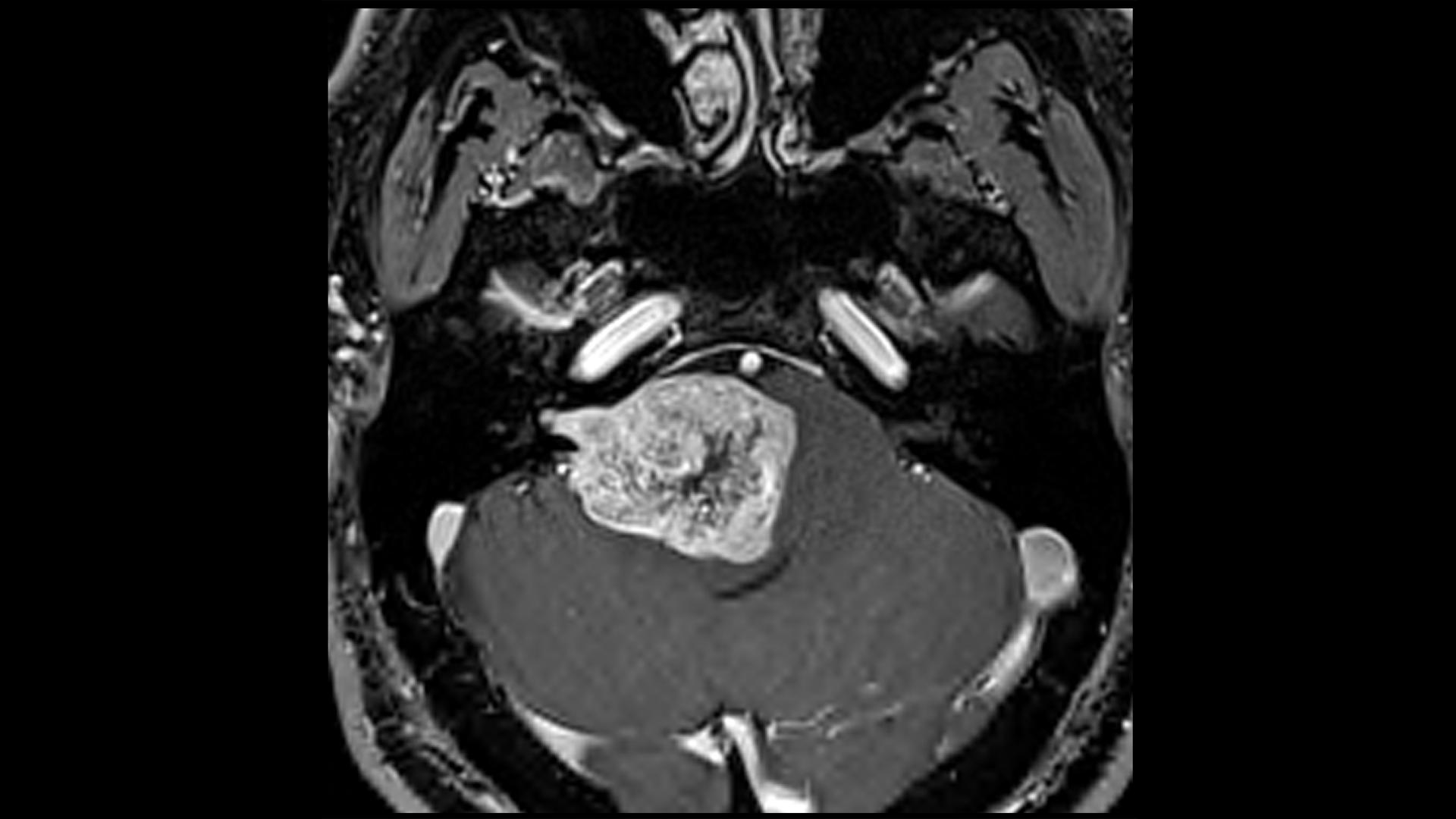

Supplement: Supplementary file 1 [file jemr-19-00062-s001.zip › jemr-4253847-supplementary/Supplementary files/1_StimuliImages/Brain_stimuli/BRAIN_7_PATH_Ax schwannoma.png]

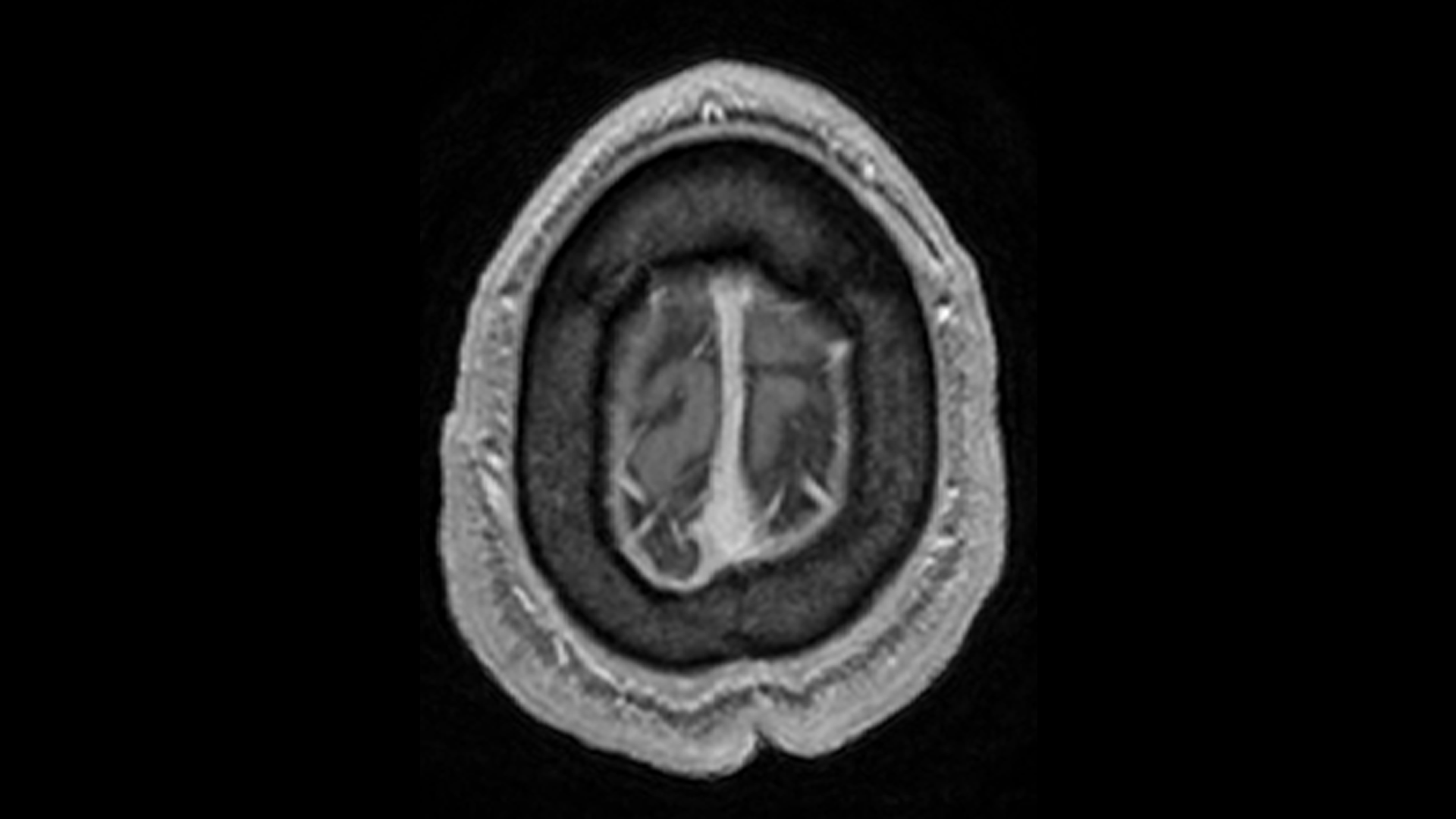

Supplement: Supplementary file 1 [file jemr-19-00062-s001.zip › jemr-4253847-supplementary/Supplementary files/1_StimuliImages/Brain_stimuli/BRAIN_9_NORMAL_Normal SSS.png]
